# Atualização de Tópicos Emergentes da Diretriz Brasileira de Insuficiência Cardíaca – 2021

**DOI:** 10.36660/abc.20210367

**Published:** 2021-06-08

**Authors:** Fabiana G. Marcondes-Braga, Lídia Ana Zytynski Moura, Victor Sarli Issa, Jefferson Luis Vieira, Luis Eduardo Rohde, Marcus Vinícius Simões, Miguel Morita Fernandes-Silva, Salvador Rassi, Silvia Marinho Martins Alves, Denilson Campos de Albuquerque, Dirceu Rodrigues de Almeida, Edimar Alcides Bocchi, Felix José Alvarez Ramires, Fernando Bacal, João Manoel Rossi, Luiz Claudio Danzmann, Marcelo Westerlund Montera, Mucio Tavares de Oliveira, Nadine Clausell, Odilson Marcos Silvestre, Reinaldo Bulgarelli Bestetti, Sabrina Bernadez-Pereira, Aguinaldo F. Freitas, Andréia Biolo, Antonio Carlos Pereira Barretto, Antônio José Lagoeiro Jorge, Bruno Biselli, Carlos Eduardo Lucena Montenegro, Edval Gomes dos Santos, Estêvão Lanna Figueiredo, Fábio Fernandes, Fabio Serra Silveira, Fernando Antibas Atik, Flávio de Souza Brito, Germano Emílio Conceição Souza, Gustavo Calado de Aguiar Ribeiro, Humberto Villacorta, João David de Souza, Livia Adams Goldraich, Luís Beck-da-Silva, Manoel Fernandes Canesin, Marcelo Imbroinise Bittencourt, Marcely Gimenes Bonatto, Maria da Consolação Vieira Moreira, Mônica Samuel Avila, Otavio Rizzi Coelho, Pedro Vellosa Schwartzmann, Ricardo Mourilhe-Rocha, Sandrigo Mangini, Silvia Moreira Ayub Ferreira, José Albuquerque de Figueiredo, Evandro Tinoco Mesquita

**Affiliations:** 1 Hospital das Clínicas da Faculdade de Medicina da Universidade de São Paulo Instituto do Coração São PauloSP Brasil Instituto do Coração (InCor) do Hospital das Clínicas da Faculdade de Medicina da Universidade de São Paulo (HCFMUSP), São Paulo, SP – Brasil.; 2 Pontifícia Universidade Católica de Curitiba CuritibaPR Brasil Pontifícia Universidade Católica de Curitiba, Curitiba, PR – Brasil.; 3 Universidade da Antuérpia Bélgica Universidade da Antuérpia, – Bélgica; 4 Hospital do Coração de Messejana FortalezaCE Brasil Hospital do Coração de Messejana Dr. Carlos Alberto Studart Gomes, Fortaleza, CE – Brasil.; 5 Hospital de Clínicas de Porto Alegre Porto AlegeRS Brasil Hospital de Clínicas de Porto Alegre, Porto Alege, RS – Brasil.; 6 Hospital Moinhos de Vento Porto AlegreRS Brasil Hospital Moinhos de Vento, Porto Alegre, RS – Brasil.; 7 Universidade Federal do Rio Grande do Sul Porto AlegreRS Brasil Universidade Federal do Rio Grande do Sul (UFRGS), Porto Alegre, RS – Brasil.; 8 Universidade de São Paulo Faculdade de Medicina de Ribeirão Preto São PauloSP Brasil Faculdade de Medicina de Ribeirão Preto da Universidade de São Paulo, São Paulo, SP – Brasil.; 9 Universidade Federal do Paraná CuritibaPR Brasil Universidade Federal do Paraná (UFPR), Curitiba, PR – Brasil.; 10 Quanta Diagnóstico por Imagem CuritibaPR Brasil Quanta Diagnóstico por Imagem, Curitiba, PR – Brasil.; 11 Universidade Federal de Goiás Hospital das Clínicas GoiâniaGO Brasil Hospital das Clínicas da Universidade Federal de Goiás (UFGO), Goiânia, GO – Brasil.; 12 Pronto Socorro Cardiológico de Pernambuco RecifePE Brasil Pronto Socorro Cardiológico de Pernambuco (PROCAPE), Recife, PE – Brasil.; 13 Universidade de Pernambuco RecifePE Brasil Universidade de Pernambuco (UPE), Recife, PE – Brasil.; 14 Universidade do Estado do Rio de Janeiro Rio de JaneiroRJ Brasil Universidade do Estado do Rio de Janeiro (UERJ), Rio de Janeiro, RJ – Brasil.; 15 Universidade Federal de São Paulo São PauloSP Brasil Universidade Federal de São Paulo (UNIFESP), São Paulo, SP – Brasil.; 16 Hospital de Coração São PauloSP Brasil Hospital de Coração (HCor), São Paulo, SP – Brasil.; 17 Hospital Israelita Albert Einstein São PauloSP Brasil Hospital Israelita Albert Einstein, São Paulo, SP – Brasil.; 18 Instituto Dante Pazzanese de Cardiologia São PauloSP Brasil Instituto Dante Pazzanese de Cardiologia, São Paulo, SP – Brasil.; 19 Universidade Luterana do Brasil CanoasRS Brasil Universidade Luterana do Brasil, Canoas, RS – Brasil.; 20 Hospital São Lucas da PUC-RS Porto AlegreRS Brasil Hospital São Lucas da PUC-RS, Porto Alegre, RS – Brasil.; 21 Hospital Pró-Cardíaco Rio de JaneiroRJ Brasil Hospital Pró-Cardíaco, Rio de Janeiro, RJ – Brasil.; 22 Universidade Federal do Acre Rio BrancoAC Brasil Universidade Federal do Acre, Rio Branco, AC – Brasil.; 23 Universidade de Ribeirão Preto Departamento de Medicina Ribeirão PretoSP Brasil Departamento de Medicina da Universidade de Ribeirão Preto (UNAERP), Ribeirão Preto, SP – Brasil.; 24 Universidade Federal Fluminense Faculdade de Medicina NiteróiRJ Brasil Faculdade de Medicina da Universidade Federal Fluminense (UFF), Niterói, RJ – Brasil.; 25 Universidade Estadual de Feira de Santana Feira de SantanaBA Brasil Universidade Estadual de Feira de Santana, Feira de Santana, BA – Brasil.; 26 Santa Casa de Misericórdia de Feira de Santana Feira de SantanaBA Brasil Santa Casa de Misericórdia de Feira de Santana, Feira de Santana, BA – Brasil.; 27 Instituto Orizonti Belo HorizonteMG Brasil Instituto Orizonti, Belo Horizonte, MG – Brasil.; 28 Hospital Vera Cruz Belo HorizonteMG Brasil Hospital Vera Cruz, Belo Horizonte, MG – Brasil.; 29 Fundação Beneficência Hospital de Cirurgia AracajuSE Brasil Fundação Beneficência Hospital de Cirurgia (FBHC-Ebserh), Aracaju, SE – Brasil.; 30 Centro de Pesquisa Clínica do Coração AracajuSE Brasil Centro de Pesquisa Clínica do Coração, Aracaju, SE – Brasil.; 31 Universidade de Brasília BrasíliaDF Brasil Universidade de Brasília (UnB), Brasília, DF – Brasil.; 32 Universidade Estadual Paulista Júlio de Mesquita Filho São PauloSP Brasil Universidade Estadual Paulista Júlio de Mesquita Filho (UNESP), São Paulo, SP – Brasil.; 33 Hospital Alemão Oswaldo Cruz São PauloSP Brasil Hospital Alemão Oswaldo Cruz, São Paulo, SP – Brasil.; 34 Hospital Regional de São José dos Campos São PauloSP Brasil Hospital Regional de São José dos Campos, São Paulo, SP – Brasil.; 35 Pontifícia Universidade Católica de Campinas CampinasSP Brasil Pontifícia Universidade Católica de Campinas (PUCC), Campinas, SP – Brasil.; 36 Universidade Estadual de Londrina Hospital Universitário LondrinaPR Brasil Hospital Universitário da Universidade Estadual de Londrina, Londrina, PR – Brasil.; 37 Hospital Universitário Pedro Ernesto Rio de JaneiroRJ Brasil Hospital Universitário Pedro Ernesto, Rio de Janeiro, RJ – Brasil.; 38 Hospital Santa Casa de Misericórdia de Curitiba CuritibaPR Brasil Hospital Santa Casa de Misericórdia de Curitiba, Curitiba, PR – Brasil.; 39 Universidade Federal de Minas Gerais Belo HorizonteMG Brasil Universidade Federal de Minas Gerais (UFMG), Belo Horizonte, MG – Brasil.; 40 Universidade Estadual de Campinas Faculdade de Ciências Médicas CampinasSP Brasil Faculdade de Ciências Médicas da Universidade Estadual de Campinas (UNICAMP), Campinas, SP – Brasil.; 41 Hospital Unimed Ribeirão Preto Ribeirão PretoSP Brasil Hospital Unimed Ribeirão Preto, Ribeirão Preto, SP – Brasil.; 42 Centro Avançado de Pesquisa Ensino e Diagnóstico (CAPED) Ribeirão PretoSP Brasil Centro Avançado de Pesquisa, Ensino e Diagnóstico (CAPED), Ribeirão Preto, SP – Brasil.; 43 Universidade Federal do Maranhão São LuísMA Brasil Universidade Federal do Maranhão (UFMA), São Luís, MA – Brasil.; 44 Treinamento Edson de Godoy Bueno / UHG Centro de Ensino Rio de JaneiroRJ Brasil Centro de Ensino e Treinamento Edson de Godoy Bueno / UHG, Rio de Janeiro, RJ – Brasil.

## Introdução

A última Diretriz de Insuficiência Cardíaca do Departamento de Insuficiência Cardíaca da Sociedade Brasileira de Cardiologia (DEIC/SBC) foi finalizada em março de 2018. A partir de então, houve um importante número de intervenções terapêuticas e abordagens diagnósticas que surgiram ou se consolidaram na prática clínica internacional e na pesquisa clínica. Ao lado disso, a pandemia da COVID-19 trouxe-nos conhecimento sobre o modelo fisiopatológico da agressão miocárdica e muitas dúvidas acerca da continuidade e da segurança dos medicamentos nos pacientes com IC crônica que apresentaram quadro agudo dessa complexa e nova entidade clínica.

Nos últimos 6 meses, trabalhamos de forma rápida e colaborativa utilizando pela primeira vez em 20 anos do DEIC as plataformas digitais para discutir, deliberar e redigir esse importante documento, optando por realizar uma revisão focada em vez de uma ampla atualização da Diretriz ainda muito recente.

Inspiramo-nos no modelo de atualização da Diretriz Canadense de Insuficiência Cardíaca de 2020,^1^ porém tivemos a nosso favor a oportunidade de observar os impactos na prática clínica e da consolidação daquelas novidades, além de ter publicado novos resultados de ensaios clínicos dos últimos 12 meses. Para apresentar esses avanços, realizamos um pioneiro encontro científico em 19 de setembro de 2020, o *I Heart Failure Summit Brazil* 2020 (digital), com cerca de 900 participantes, muitos destes associados do DEIC.

A liderança da Diretoria Científica foi fundamental para a organização de diferentes grupos de trabalhos e elaboração de uma forma prática e segura de discussão e votação. Garantindo o distanciamento social e empregando a tecnologia digital, o encontro permitiu amplos debates sobre os diferentes pontos de vista alicerçados nas melhores evidências científicas.

No presente documento, o DEIC/SBC apresenta uma revisão e uma atualização detalhadas de sua Diretriz de Insuficiência Cardíaca Crônica. Os trabalhos tiveram início no mês de julho de 2020, com a definição do Comitê Editor, que estabeleceu prioridades, dividiu os 52 participantes em grupos de trabalho e definiu o cronograma das atividades. Estes grupos, compostos por cinco a sete participantes cada, deram início a intensas discussões virtuais que culminaram com a redação de tabelas preliminares, sendo posteriormente amplamente divulgadas e revisadas pelo Comitê Revisor composto por 11 membros. As discussões finais foram realizadas em plenária virtual em 4 de dezembro de 2020, com a participação de todos os colaboradores, nas quais as principais recomendações foram votadas. As decisões quanto às classes de recomendação foram definidas com a concordância de mais de 75% dos participantes.

As definições de Classes de Recomendação e Nível de Evidência respeitam as mesmas normas da última diretriz, conforme preconiza o SBC/CONDir para elaboração de diretrizes e são assim descritas:

**Table:** 

**Classes de Recomendação**	
Classe I	Condições para as quais há evidências conclusivas ou, em sua falta, consenso geral de que o procedimento é seguro e útil/eficaz
Classe II	Condições para as quais há evidências conflitantes e/ou divergência de opinião sobre segurança, e utilidade/eficácia do procedimento
Classe IIA	Peso ou evidência/opinião a favor do procedimento. A maioria aprova
Classe IIB	Segurança e utilidade/eficácia menos bem estabelecida, não havendo predomínio de opiniões a favor
Classe III	Condições para as quais há evidências e/ou consenso de que o procedimento não é útil/eficaz e, em alguns casos, pode ser prejudicial
**Níveis de Evidência**	
Nível A	Dados obtidos a partir de múltiplos estudos randomizados de bom porte, concordantes e/ou de metanálise robusta de estudos clínicos randomizados
Nível B	Dados obtidos a partir de metanálise menos robusta, a partir de um único estudo randomizado ou de estudos não randomizados (observacionais)
Nível C	Dados obtidos de opiniões consensuais de especialistas

As recomendações terapêuticas propostas no presente documento embasam-se nas evidências científicas mais atuais, considerando não apenas os aspectos de eficácia clínica demonstrados em grandes ensaios clínicos. Buscamos sumarizar as principais recomendações em fluxogramas e algoritmos de fácil entendimento e grande aplicabilidade clínica, propondo abordagens para o diagnóstico e o tratamento da insuficiência cardíaca.

Nosso compromisso com a comunidade científica, ligado à pesquisa e à assistência aos pacientes com insuficiência cardíaca, gestores públicos e privados e também formuladores de políticas públicas, certamente contará com um documento que buscou apresentar as intervenções científicas de forma didática e, assim, facilitar sua implantação nas diferentes esferas de atendimento do paciente com insuficiência cardíaca.

**Dr. Evandro Tinoco Mesquita**

## 1. Inovações em Insuficiência Cardíaca com Fração de Ejeção Preservada (ICFEp), Levemente Reduzida (ICFElr) e Melhorada (ICFEm)

### 1.1. Diagnóstico de Insuficiência Cardíaca com Fração de Ejeção Preservada (ICFEp)

**Figura 1.1:**
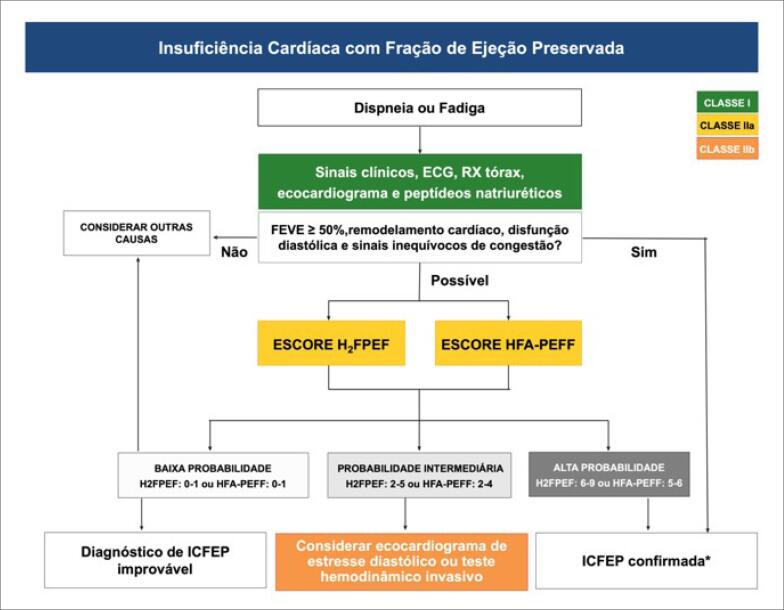
Fluxograma diagnóstico de insuficiência cardíaca com fração de ejeção preservada (ICFEp)

No paciente com dispneia ou fadiga inexplicada, a avaliação da probabilidade pré-teste para insuficiência cardíaca (IC) deve ser efetuada com dados clínicos, eletrocardiográficos, ecocardiográfico e laboratorial. A seguir, na figura 1.1, os dois sistemas de escores desenvolvidos para confirmação deste diagnóstico: tanto a pontuação H_2_FPEF (esquerda) quanto a HFA-PEFF (direita) podem ser utilizadas (Tabelas 1.1 e 1.2). Nesses modelos, os pacientes considerados de alta e baixa probabilidade são considerados como tendo ou não insuficiência cardíaca com fração de ejeção preservada (ICFEp), respectivamente. Nos pacientes com probabilidade intermediária, a avaliação da função diastólica durante estresse, que pode ser realizado por meio de teste hemodinâmico invasivo ou ecocardiografia de estresse diastólico, é capaz de auxiliar no diagnóstico de ICFEp. Nos pacientes com baixa probabilidade para a ICFEp, recomenda-se a investigação de outras causas de dispneia e fadiga^2^ (Figura 1.1 e Tabela 1.3).

*Adaptado de: Borlaug BA.*^*2*^*Nat Rev Cardiol. 2020; 17:559-573. ECG: eletrocardiograma; FEVE: fração de ejeção preservada; IC: insuficiência cardíaca.*

**Tabela 1.1:** Escore H_2_FPEF para o diagnóstico da insuficiência cardíaca com fração de ejeção preservada (ICFEp)

	Variável Clínica	Características	Pontos
H_2_	Obesidade *(****H****eavy)*	IMC > 30 Kg/m^2^	2
	**H**ipertensão	2 ou mais anti-hipertensivos	1
F	**F**ibrilação atrial	Paroxística ou persistente	3
P	Hipertensão **P**ulmonar	PSAP > 35 mmHg (ecocardiograma)	1
E	Idade avançada (***E****lderly*)	Idade > 60 anos	1
F	Pressões de enchimento (***F****illing pressures*)	E/e´> 9	1

*Adaptado de Reddy YNV et al.*^*5*^*Circulation. 2018; 138:861-870. IMC: índice de massa corpórea; PSAP: pressão sistólica da artéria pulmonar.*

**Tabela 1.2:** Escore HFA PEFF para diagnóstico de insuficiência cardíaca com fração de ejeção preservada (ICFEp)

CRITÉRIOS	MAIOR (2 Pontos)	MENOR (1 ponto)
FUNCIONAL	e’ septal < 7 ou e’ lateral < 10 ou E/e’ > 15 ou Velocidade RT > 2,8 m/s (PSAP > 35 mmHg)	E/e’ 9-14 ou GLS < 16%
MORFOLÓGICO	VAEI > 34 mL/m^2^ ou Massa VE . 149/122 g/m^2^ (H/M) e ERP > 0,42	VAEi 29 - 34 mL/m^2^ ou Massa VE > 115/95 g/m^2^ (H/M) ou ERP > 0,42 ou Septo ou PP ≥ 12 mm
BIOMARCADOR (Ritmo sinusal) BIOMARCADOR (Fibrilação atrial)	NT-proBNP > 220 pg/mL ou BNP > 80 pg/mL NT-proBNP > 660 pg/mL ou BNP > 240 pg/mL	NT-proBNP 125 - 220 pg/mL ou BNP 35 - 80 pg/mL NT-proBNP 365 - 660 pg/mL ou BNP 105 - 240 pg/mL

*Adaptado de Pieske B et al.*^*7*^*Heart Failure Association (HFA) of the European Society of Cardiology (ESC). Eur J Heart Fail. 2020; 22:391-412.*

*Velocidade RT: velocidade do fluxo de regurgitação da valva tricúspide; GLS: strain global longitudinal; VAEi: índice de volume atrial esquerdo; BNP: peptídeo natriurético do tipo B; NT-proBNP: peptídeo natriurético N-terminal pró-tipo B; VE: ventricular esquerda; H: homens / M:mulheres; ERP: espessura relativa da parede; PP: parede posterior.*

**Tabela 1.3:** Recomendações para o diagnóstico de insuficiência cardíaca com fração de ejeção preservada (ICFEp)

Recomendações	Classe	NE	Comentários	Tabela 2018	Ref.
Peptídeos natriuréticos para rastreamento de ICFEp.	I	B	**NOVA:** Devem ser consideradas a ampla variação do nível sérico dos peptídeos natriuréticos nessa população e as condições que modificam sua acurácia, como a fibrilação atrial e a obesidade.	Nova	3, 4
Ecocardiograma completo para confirmação do diagnóstico.	I	B	**NOVA:** Exame com apresentação de índices de Doppler para estimativa de pressões diastólica e pulmonar, bem como índices de volume e massa cardíaca indexados à superfície corporal.	Nova	4, 5
Escore diagnósticos H_2_FPF ou HFA PEFF para melhorar a acurácia diagnóstica para ICFEp em pacientes com suspeita clínica.	IIa	B	**NOVA:** Escores com validação em coortes retrospectivas.	Nova	6–8
Avaliação durante estresse da função diastólica por ecocardiografia ou avaliação hemodinâmica invasiva em caso de dúvida diagnóstica após a aplicação dos escores H_2_FPF ou HFA PEFF.	IIb	B	**NOVA:** Escores com validação em coortes retrospectivas.	Nova	9,10
A estratégia inicial para o diagnóstico de ICFEp é a determinação da probabilidade pré-teste para IC, através do uso de achados clínicos associados a exames complementares como: eletrocardiograma, radiografia do tórax, ecocardiograma e peptídeos natriuréticos, se disponíveis. Para a interpretação do resultado dos peptídeos natriuréticos, é importante considerar que há grande variação dos níveis séricos nessa população e que, na vigência de fibrilação atrial (FA), devemos considerar pontos de corte mais elevados.^3^^,^^4^ Na presença de plausibilidade para IC, é razoável a aplicação dos escores H_2_FPEF^5^^,^^6^ (com dados clínicos e ecocardiográficos) e HFA PEFF^7^ (com dados ecocardiográficos completos e de peptídeos natriuréticos), os quais já foram validados em populações externas.^6^^,^^8^ para determinar a probabilidade alta, intermediária e baixa. Em pacientes com baixa probabilidade de ICFEp, sugere-se a busca objetiva para outras etiologias para dispneia. Nos indivíduos com probabilidade intermediária, estudos recentes demonstram que a observação dos dados diastólicos sob estresse físico pode revelar pacientes com resposta anormal, constituindo assim uma estratégia diagnóstica que pode ser não invasiva (observação diastólica por ecocardiograma)^9^ ou invasiva (cateterização da artéria pulmonar).^10^ Para a realização dos escores supracitados, faz-se necessária a realização do ecocardiograma completo, ou seja, exame com a extração dos diâmetros, índices de volume do átrio esquerdo e Doppler de fluxo, Doppler tecidual (e’ septal e ou lateral) e, se possível, com dados da deformação miocárdica (*strain* e *strain rate*).^5^					

*FA: fibrilação atrial; IC: insuficiência cardíaca; ICFEp: insuficiência cardíaca com fração de ejeção preservada.*

### 1.2. Tratamento da Insuficiência Cardíaca com Fração de Ejeção Preservada (ICFEp)

Até o momento, ainda não há intervenção específica que reduza eventos cardiovasculares de pacientes com ICFEp. Os ensaios clínicos que avaliaram o uso de inibidores da enzima de conversão de angiotensina II (iECA), bloqueadores dos receptores de angiotensina II (BRA), inibidores da neprilisina e antagonistas dos receptores de angiotensina II (INRA) e espironolactona foram neutros quanto à redução do risco de eventos comparado ao placebo para pacientes com ICFEp.^11^^–^^14^ A análise de subgrupo, de acordo com a fração de ejeção, mostrou de maneira consistente a ausência de benefício nos subgrupos com fração de ejeção mais elevada (acima de 50%). Achado semelhante foi encontrado para betabloqueadores em metanálise de ensaios clínicos randomizados.^13^ Por isso, as recomendações da diretriz de 2018 para o tratamento farmacológico da ICFEp continuam mantidas, incluindo o uso de diuréticos para aliviar congestão e o tratamento de comorbidades como a isquemia miocírdica, a fibrilação atrial e a hipertensão, para diminuir sintomas e potencialmente reduzir a progressão da ICFEp.^15^ Por isso, é fundamental que se investiguem condições potencialmente reversíveis e associadas à ICFEp “secundária”, como as cardiomiopatias infiltrativas e restritivas, além de considerar causas alternativas de intolerância ao esforço.

### 1.3. Tratamento da Insuficiência Cardíaca com Fração de Ejeção Levemente Reduzida (ICFElr) (Tabela 1.4)

**Tabela 1.4:** Recomendações para o tratamento de insuficiência cardíaca com fração de ejeção levemente reduzida (ICFElr)

Recomendações	Classe	NE	Comentários	Tabela 2018	Ref.
Bisoprolol, carvedilol ou succinato de metoprolol para pacientes com ICFElr em ritmo sinusal para reduzir morbidade e mortalidade.	IIa	A	**NOVA:** Os dados atualmente disponíveis indicam que a resposta de pacientes com ICFElr ao tratamento da IC é semelhante à de pacientes com ICFEr.	Nova	13
iECA ou BRA para reduzir morbidade e mortalidade.	IIa	B		Nova	11
Espironolactona para reduzir morbidade e mortalidade	IIa	B		Nova	12
Sacubitril-valsartana em substituição ao iECA (ou BRA), para pacientes sintomáticos já em uso de terapêutica otimizada com terapia tripla para reduzir hospitalização.	IIa	B		Nova	14
Em que pese a inexistência de estudos que tenham avaliado intervenções terapêuticas dirigidas especificamente a pacientes com insuficiência cardíaca com fração de ejeção levemente reduzida (ICFElr), as análises secundárias de ensaios clínicos em pacientes com ICFEr e ICFEp indicam que pacientes com ICFElr (fração de ejeção ventricular esquerda, FEVE 41-49%) podem se beneficiar das intervenções correntemente indicadas a pacientes com ICFEr (FEVE≤40%). Em metanálise de 11 estudos controlados e randomizados observou-se que os betabloqueadores se associaram a menor mortalidade em pacientes com ICFEi e ritmo sinusal.^13^ Em subanálise do estudo *Topcat*, foi identificado o efeito benéfico da espironolactona na mortalidade cardiovascular de pacientes com FEVE entre 44-50%;^12^ em subanálise do CHARM houve benefício com candesartan no desfecho combinado de mortalidade cardiovascular e hospitalizações nos pacientes com FEVE de 40% a 49%.^11^ A análise combinada dos estudos *PARAGON-HF* e *PARADIGM-HF* sugeriu que sacubitril-valsartana está associada à redução de hospitalizações em níveis intermediários de FEVE, sendo este efeito mais intenso entre os pacientes do sexo feminino com valores mais elevados de FEVE.^14^					

Esta diretriz utiliza as denominações e definições de acordo com a nova classificaçao universal de IC. ICFEp: insuficiência cardíaca com fração de ejeção preservada; ICFElr: insuficiência cardíaca com fração de ejeção levemente reduzida; ICFEr: insuficiência cardíaca com fração de ejeção reduzida; FEVE: fração de ejeção ventricular esquerda; iECA: inibidores da enzima de conversão de angiotensina II; BRA: bloqueadores dos receptores de angiotensina II.

### 1.4. Tratamento da Insuficiência Cardíaca com fração de Ejeção Melhorada (ICFEm) (Tabela 1.5)

**Tabela 1.5:** Recomendações para o tratamento de insuficiência cardíaca com fração de ejeção melhorada (ICFEm)

Recomendações	Classe	NE	Comentários	Tabela 2018	Ref.
Manutenção da terapêutica modificadora de prognóstico utilizada no tratamento da ICFEr por cardiomiopatia dilatada melhorada.	I	B	**NOVA:** Indicação respaldada por estudo randomizado multicêntrico com amostra limitada e com desfechos substitutos.	Nova	16
O avanço no tratamento da IC com fração de ejeção reduzida (ICFEr) tem determinado a melhora na FEVE e a redução no tamanho do ventrículo esquerdo em cerca de 40% dos pacientes, dependendo da etiologia.^17^ Recentemente, foi publicada a nova definição e classificação universal de IC, que recomenda o termo IC com fração de ejeção melhorada (ICFEm) para pacientes com FEVE prévia < 40% e tiveram um aumento de 10 pontos percentuais atingindo taxas acima de 40%. Esta classificação universal recomenda que seja utilizado o termo “melhorada” ao invés de “recuperada”.^18^ Halliday BP et al.^16^ testaram a segurança de retirar a medicação para IC em um grupo pequeno de pacientes com cardiomiopatia dilatada recuperada em um ensaio clínico piloto, sem cegamento, mas conduzido de modo randomizado e multicêntrico. Os critérios de inclusão foram: diagnóstico prévio de cardiomiopatia dilatada com FEVE menor ou igual a 40%; ausência de sintomas de insuficiência cardíaca; tratamento com diurético de alça e medicações modificadoras de prognóstico; FEVE atual maior ou igual que 50%; volume diastólico final do ventrículo esquerdo indexado à superfície corporal normal e NT-proBNP menor que 250 pg/mL. Os pacientes foram randomizados para a retirada das medicações por 6 meses e o desfecho primário foi considerado uma combinação de redução da FEVE, dilatação do VE e retorno dos sintomas de IC. Após 6 meses de seguimento, 44% dos pacientes que tiveram sua medicação retirada apresentaram algum dos critérios para desfecho primário, comparados a nenhum participante do grupo de tratamento mantido, registrando-se uma taxa de eventos estimada em 45,7% (IC 95% 28,5–67,2; p = 0,0001). Este estudo, apesar de pequeno e com desenho aquém do ideal, é a melhor evidência até o momento sobre essa população, sugerindo que a manutenção dos fármacos nesse contexto seja a melhor estratégia, pelo menos até a publicação de estudo mais robusto.					

*Esta diretriz utiliza as denominações e definições de acordo com a nova classificaçao universal de IC.*^*18*^*FEVE: fração de ejeção ventricular esquerda; ICFEr: insuficiência cardíaca com fração de ejeção reduzida; NT-proBNP: fração N-terminal do peptídeo natriurético atrial do tipo B.*

## 2. Inovações em Amiloidose Cardíaca

Assistimos recentemente a grandes avanços no conhecimento da amiloidose cardíaca, o que acarretou na profunda transformação do seu significado clínico, epidemiológico e no surgimento de tratamentos específicos. Várias evidências sugerem que a amiloidose cardíaca não seja uma doença rara, mas uma condição amplamente subdiagnosticada, considerada hoje uma causa relativamente comum e tratável de insuficiência cardíaca com fração de ejeção preservada (ICFEp), particularmente a amiloidose cardíaca ligada à transtirretina (ATTR) na sua forma selvagem ou sistêmica senil (ATTR-wt), cujo diagnóstico tem aumentado de forma expressiva.^19^^–^^22^

Trata-se de uma doença multisistêmica causada pela deposição tecidual de proteínas fibrilares insolúveis que perdem a sua conformação, o que leva à disfunção orgânica, inclusive do coração. Mais de 30 tipos de proteínas amiloidogênicas são descritas,^23^ sendo duas delas responsáveis por 95% dos casos de acometimento cardíaco: a amiloidose por cadeia leve (AL), esta relacionada com a produção monoclonal de imunoglobulinas devido à discrasia de plasmócitos; e amiloidose pela transtiretina (ATTR), a proteína transportadora de retinol e tiroxina produzida pelo fígado, que pode ter caráter secundário à sua mutação (ATTRm) ou ser selvagem (ATTRwt), causada por alterações pós-transcricionais e as proteínas de chaperonas, ligadas ao envelhecimento.

A AL apresenta incidência de 6 a 10 milhões de pessoas por ano e era considerada a principal causa de amiloidose cardíaca.^24^ No entanto, com o desenvolvimento de técnicas não invasivas de diagnóstico e o surgimento de tratamentos efetivos, o diagnóstico da ATTR, especialmente da ATTRwt, tem aumentado significativamente.^19^ Estudos demonstram ATTR em até 13% dos pacientes com ICFEp e espessamento da parede ventricular esquerda maior que 12 mm,^20^ sendo que até 25% das necropsias de muito idosos apresentam TTR no coração.^22^ A ATTRm apresenta um caráter autossômico dominante, com mais de 130 mutações descritas, que causam variações nos fenótipos de acometimento neurológico e cardíaco.

### 2.1. Quando Suspeitar de Amiloidose

Tendo em vista que a ATTR, particularmente a ATTRwt, é uma condição mais prevalente do que se antecipava, é importante suspeitar dessa condição na presença de pistas clínicas para posterior investigação diagnóstica (Tabela 2.1). Por se tratar de uma forma de cardiomiopatia restritiva infiltrativa, o padrão típico é espessamento da parede ventricular, da disfunção diastólica e dos distúrbios de condução. Em certos contextos clínicos, é necessário o diagnóstico diferencial com cardiomiopatia hipertrófica, ICFEp,^25^ bloqueios atrioventriculares avançados e arritmias atriais sem causa aparente. A concomitância de ATTRwt e estenose aórtica cálcica pode causar hipertrofia ventricular acentuada e se apresentar como estenose aórtica de baixo fluxo e baixo gradiente.

**Tabela 2.1:** Pistas clínicas para o diagnóstico de amiloidose

**História e Exame Físico**
ICFEp, particularmente em homens idosos (maiores de 65 anos)
Intolerância ao iECA/BRA/INRA e ou betabloqueadores
Síndrome do túnel do carpo bilateral
Estenose do canal vertebral
Ruptura do tendão do bíceps
Polineuropatia periférica não explicada e/ou disfunção autonômica (hipotensão postural; diarreia alternada com constipação; disfunção erétil)
Equimose periorbitária
Macroglossia
**Pistas Originadas dos Exames de Imagem**
Espessamento concêntrico das paredes do VE com amplitude do QRS reduzida ou não aumentada proporcionalmente ao grau de aumento da espessura das paredes do VE
Fenótipo infiltrativo ao ecocardiograma (SIV>12mm), hiperrefringência miocárdica, hipertrofia biventricular, derrame pericárdico, espessamento valvar, espessamento de septo interatrial
Redução do *strain* longitudinal que poupa a região apical *(apical sparing)*
Enchimento ventricular esquerdo de padrão restritivo, com espessamento das paredes do ventrículo direito
Realce tardio de contraste na ressonância magnética cardíaca de padrão subendocárdico ou transmural, difuso ou aumento do volume extracelular
**Pistas Combinadas**
Insuficiência cardíaca exibindo cavidade ventricular esquerda não dilatada e com espessamento das paredes (septo interventricular maior que 12 mm), especialmente em pacientes sem hipertensão arterial sistêmica pregressa.
Apresentação clínica de cardiomiopatia hipertrófica iniciada tardiamente (acima de 60 anos)
Estenose aórtica com espessamento das paredes do ventrículo direito, particularmente nos casos paradoxais com baixo fluxo/baixo gradiente

*ICFEp: insuficiência cardíaca de fração com ejeção preservada; FEVE: fração de ejeção ventricular esquerda; iECA: inibidores da enzima de conversão de angiotensina II; BRA: bloqueadores dos receptores de angiotensina II; INRA: inibidores da neprilisina e antagonistas dos receptores de angiotensina II.*

Além disso, certas manifestações multisistêmicas podem levantar suspeita de ATTR: síndrome de túnel do carpo bilateral, ruptura do tendão do bíceps, hipotensão ortostática, estenose do canal vertebral, alterações digestivas e intolerância a medicações anti-hipertensivas.^26^ A história familiar é muito importante nas formas hereditárias da amiloidose, que apresentam prognóstico pior do que nos pacientes acometidos com a forma selvagem da doença.

### 2.2. Diagnóstico de Amiloidose Cardíaca (Tabela 2.1)

Diante da suspeita da doença, o primeiro passo é investigar a presença de cadeias leves de imunoglobulinas para o diagnóstico da AL, uma vez que essa forma da amiloidose cardíaca exige tratamento específico com quimioterápicos e o prognóstico piora muito com o retardo no início do tratamento. A confirmação da AL depende da detecção da proteína amiloide em tecidos envolvidos (biopsia), mas a forma ATTR pode ser confirmada não invasivamente, mediante emprego de cintilografia cardíaca com radiotraçadores ósseos. No Brasil, é usado o Tc-99m-pirofosfato.

### 2.3. Métodos Diagnósticos

#### 2.3.1. Eletrocardiograma

A baixa voltagem no complexo QRS é achado comum na AL, sendo menos prevalente na ATTR (aproximadamente 30% dos casos), sendo mais comum a discrepância entre a magnitude da hipertrofia ao ecocardiograma e a amplitude dos complexos QRS. Fibrilação atrial e o padrão de “pseudoinfarto” também podem ser encontrados.

#### 2.3.2. Ecocardiograma

É um dos principais exames para levantar a suspeita. Entre os achados sugestivos se destacam o espessamento da parede ventricular esquerda maior que 12 mm, especialmente na ausência de hipertensão arterial, aumento bi-atrial e desproporcional ao tamanho dos ventrículos, espessamento das valvas atrioventriculares e do septo interatrial, e o aumento da ecogenicidade do miocárdio com aparência granular. O índice de deformação sistólica longitudinal do miocárdio ou *strain* sistólico longitudinal pode mostrar a preservação da contratilidade do ápice do ventrículo esquerdo com relação aos demais segmentos (*apical sparing* ou imagem de “cereja de bolo”).^27^

#### 2.3.3. Cintilografia Cardíaca com Radiotraçadores Ósseos

Cintilografia cardíaca com radiotraçadores ósseos, como Tc99m-pirofosfato usado no Brasil, pode ser utilizada para o diagnóstico diferencial entre a amiloidose AL e ATTR, esta última mostrando captação miocárdica anômala com intensidade maior ou equivalente à óssea. No entanto, a captação cardíaca pode ocorrer, ainda que mais discreta, em até 30% dos casos de AL. A captação cardíaca intensa (grau 2 ou 3), em conjunto com ausência de cadeias leves nos exames bioquímicos, tem especificidade de 100% para ATTR, podendo dispensar a biopsia cardíaca para o diagnóstico da doença.^19^

#### 2.3.4. Ressonância Magnética Cardíaca

A Ressonância Magnética Cardíaca possui alta sensibilidade e especificidade para o diagnóstico, sendo útil também para diferenciar a amiloidose cardíaca de outras miocardiopatias. A deposição amiloide no miocárdio causa aumento no volume de distribuição do contraste paramagnético nas regiões do miocárdio em que os cardiomiócitos são substituídos ou deslocados por fibrose ou inflamação, cursando com padrão de realce tardio (RT) mais comumente subendocárdico difuso e circunferencial do ventrículo esquerdo, ainda que realces tardios transmural e difuso também possam ser encontrados.^27^

### 2.4. Tratamento de Amiloidose Cardíaca por Transtirretina (AC-ATTR) (Tabela 2.2)

**Tabela 2.2:** Recomendações para o tratamento específico da amiloidose cardíaca por transtirretina (AC-ATTR)

Recomendação	Classe	NE	Comentário	Tabela 2018	Ref.
Tafamidis 80 mg/dia, para o tratamento de pacientes com amiloidose cardíaca por transtirretina para redução da mortalidade e de hospitalizações cardiovasculares.	I	B	**NOVA:** Estudo clínico randomizado multicêntrico respalda esta recomendação.	Nova	28
Várias etapas do processo de formação das fibrilas amiloides constituem alvos terapêuticos na amiloidose por transtirretina (ATTR). A primeira terapia modificadora da doença que demonstrou evidência de benefício em pacientes com cardiomiopatia amiloide é um estabilizador dos tetrâmeros da TTR, o tafamidis. Esse fármaco foi testado em um estudo clínico multicêntrico e randomizado contra o placebo, envolvendo 441 pacientes com AC e no qual 264 deles receberam o medicamento em doses de 20 mg ou 80 mg ao dia *(estudo ATTR-ACT [Tafamidis Treatment for Patients with Transthyretin Amyloid Cardiomyopathy])*.^28^ Os principais resultados mostraram que o uso de tafamidis se associou à redução de 30% no desfecho primário de mortalidade por qualquer causa (RR = 0,70 [IC 95%: 0,51-0,96]), além de reduzir as internações por causa cardiovascular em 32% (RR = 0,68 [IC95%: 0,56 −0,81]) e a piora da capacidade funcional e da qualidade de vida. Esses resultados embasaram, no Brasil, a aprovação pela Anvisa do uso do fármaco para o tratamento da amiloidose cardíaca por TTR na dose de 80 mg/dia.^28^					

*ATTR: amiloidose por transtirretina; RR: risco relativo.*

Frente à importância clínica e epidemiológica, além de novas terapias emergentes para esta doença, um Posicionamento sobre Diagnóstico e Tratamento da Amiloidose Cardíaca será publicado em breve, abordando de forma mais ampla os diferentes aspectos da doença.

## 3. Inovações em Telemonitoramento na Insuficiência Cardíaca (Tabela 3.1)

**Tabela 3.1:** Recomendações de telemonitoramento, *werables*, inteligência artificial e *machine learning* na insuficiência cardíaca

Recomendações	Classe	NE	Comentário	Tabela 2018	Ref.
Uso de monitoramento e suporte à distância (telemonitoramento) para manejo de pacientes com insuficiência cardíaca crônica.	IIa	A	**NOVA:** Existem metanálises mostrando diminuição na mortalidade e na internação por IC.	Nova	29–32
*Wearables* como ferramentas auxiliares no manejo diagnóstico, terapêutico e de reabilitação em pacientes com insuficiência cardíaca crônica ou aguda.	IIa	B	**NOVA:** Vários estudos observacionais mostram o benefício do uso de *wearables* em paciente com IC.	Nova	33, 34
Uso da Inteligência artificial no diagnóstico, avaliação de prognóstico, ou seleção de pacientes com maior benefício para diversas terapias.	IIb	B	**NOVA:** Estudos observacionais apontam para o benefício do uso de *Machine Learning* e Inteligência Artificial no diagnóstico e prognóstico da IC.	Nova	35
As metanálises envolvendo estudos observacionais e randomizados de monitoramento e suporte à distância invasivo ou não invasivo têm mostrado impacto positivo no prognóstico de pacientes com IC.^29^^–^^32^ A redução na mortalidade geral pode variar de 19 a 31% com o telemonitoramento em pacientes com IC, enquanto a redução na frequência de internação hospitalar por IC varia de 27 a 39%, principalmente em pacientes em classe funcional (CF) III/IV, segundo a New York Heart Association (NYHA). A inteligência artificial apresenta aplicações em estudo para IC, seja como diagnóstico, avaliação de prognóstico, telemonitoramento ou ainda para selecionar pacientes com maior benefício para diversas terapias.^33^^,^^34^ Isso pode ser feito, por exemplo, na distinção de fenótipos, alocando pacientes em diferentes perfis de assinatura de doença; na melhor acurácia para o diagnóstico de IC aguda com relação ao médico; e no eventual direcionamento para terapias novas ou já estabelecidas, como análise adicional do ECG basal para identificar paciente melhor respondedor à terapia de ressincronização cardíaca.^35^					

*CF: classe funcional; IC: insuficiência cardíaca.*

## 4. Inovações em Cardiointervenção

### 4.1. Abordagem Percutânea da Insuficiência Mitral Secundária (Tabela 4.1)

**Tabela 4.1:** Recomendações para intervenção percutânea na insuficiência mitral grave secundária

Recomendação	Classe	NE	Comentários	Tabela 2018	Ref.
**Clipagem percutânea da valva mitral**					
**Isquêmica ou dilatada**					
Sintomas refratários (classe funcional ≥ II) ao tratamento clínico convencional e após avaliação do *Heart Team*.	IIa	B	**NOVA:** Estudo randomizado com desfecho de morte respalda esta recomendação.	Item 11.3 (página 467)	36
Recomendamos que a terapia guiada por diretrizes esteja otimizada, incluindo terapia de ressinconização cardíaca e revascularização, quando apropriado, antes da consideração do tratamento percutâneo da insuficiência mitral (IM) para pacientes com ICFEr e IM grave. O estudo *COAPT (Transcatheter Mitral-Valve Repair in Patients with Heart Failure)* que avaliou se o uso do dispositivo *edge-to-edge* poderia beneficiar pacientes com IM secundária moderadamente grave ou grave (EROA maior ou igual a 30 mm^2^ e/ou volume regurgitante maior que 45 mL) com FEVE de 20% a 50%, diâmetro sistólico final do VE menor que 7 cm e sintomas persistentes, apesar da terapia baseada em evidências maximizada, com participação de equipe multidisciplinar experiente na avaliação e tratamento da IC e IM.^36^ A atualização da Diretriz de Valvopatia de 2020 não fez esta diferenciação na seleção dos pacientes. Visando manter a linearidade das diretrizes, optamos por também não incluir esta diferenciação em nossa recomendação.^37^					

*ICFEr: insuficiência cardíaca com fração de ejeção reduzida; IM: insuficiência mitral; VE: ventrículo esquerdo; IC: insuficiência cardíaca; FEVE: fração de ejeção ventricular esquerda. NYHA: New York Heart Association.*

### 4.2. Ablação de Fibrilação Atrial (Tabela 4.2)

**Tabela 4.2:** Recomendações para ablação de fibrilação atrial na insuficiência Cardíaca com Fração de Ejeção reduzida (ICFEr)

Recomendação	Classe	NE	Comentários	Tabela 2018	Ref.
Ablação de FA para restaurar o ritmo sinusal em pacientes sintomáticos, intolerantes ou refratários a fármacos antiarrítmicos para redução de mortalidade e hospitalizações por IC.	IIa	B	Recomendação de 2018 mantida.	Item 10.1 (página 465)	Vide 2018
Ablação de FA como alternativa ao tratamento clínico com fármacos antiarrítmicos para sintomas refratários ou pacientes intolerantes.	I	A	**NOVA:** Estudos randomizados demonstram maior taxa de sucesso de manutenção do ritmo sinusal com ablação da FA, além da ausência dos efeitos colaterais causados pelos fármacos antiarrítmicos.	Item 10.1 (página 465)	38–43
Ablação de FA para promover remodelamento reverso em pacientes com taquicardiomiopatia induzida pela FA, se refratários ao tratamento medicamentoso ou na preferência do paciente pela ablação, independentemente dos sintomas.	I	B	**NOVA:** Estudo randomizado demonstrou a capacidade da ablação da FA em promover remodelamento reverso em pacientes com IC por provável taquimiocardiopatia.	Item 10.1 (página 465)	39,44
A ablação de fibrilação atrial (FA) em pacientes com IC tem um benefício maior do que o uso de fármacos antiarrítmicos pela maior taxa de manutenção de ritmo sinusal, melhora de capacidade funcional e qualidade de vida, CF, distância no teste de caminhada de 6 minutos, VO_2_ máximo e redução de biomarcadores (BNP). Ela pode ser considerada uma terapia alternativa para controle de sintomas em pacientes com FA e que são intolerantes ou refratários ao tratamento com antiarrítmicos ou mesmo como terapia inicial.^38^^–^^43^ Remodelamento reverso foi observado em diversos estudos com ablação de FA, gerando incremento de FEVE.^38^^–^^42^^,^^44^ Quando a etiologia da IC é desconhecida e considera-se taquicardiomiopatia induzida pela FA como etiologia possível, o incremento esperado de FEVE com a ablação é ainda mais significativo.^39^^,^^44^ Estudos também demostraram redução de 45% de hospitalização por IC, 47-56% mortalidade por qualquer causa e de 38% de morte ou hospitalização por IC.^41^^,^^42^^,^^44^ Entretanto, a taxa de sucesso varia em torno de 60 a 80% no primeiro ano e a doença cardíaca estrutural é um fator de risco para recorrência.^45^ O isolamento das veias pulmonares pode ser feito por radiofrequência ou crioablação e essas técnicas podem ser combinadas com ablação de outros substratos.					

*CF: classe funcional; FA: fibrilação atrial; IC: insuficiência cardíaca. BNP: peptídeo natriurético do tipo B; FEVE: fração de ejeção ventricular esquerda.*

## 5. COVID-19 e a Insuficiência Cardíaca (Tabela 5.1)

**Tabela 5.1:** Recomendações relacionadas com o manejo da COVID-19 em pacientes com insuficiência cardíaca

Recomendação	Classe	NE	Comentários	Tabela 2018	Ref.
Testagem com RT-PCR para SARS-CoV-2 em indivíduos com IC crônica e manifestações respiratórias agudas.	I	C	**NOVA:** Editoriais e recomendações de sociedades (publicação on-line).	Nova	46,47
Manutenção de iECA, BRA ou INRA em indivíduos com IC crônica que desenvolvam COVID-19, na ausência de hipotensão ou sinais de comprometimento hemodinâmico.	I	C	**NOVA:** Estudos observacionais controlados com número grande de pacientes, mas com percentual menor de pacientes com IC.	Nova	48–50
Acompanhamento ambulatorial de pacientes com IC por meio de visitas virtuais e telemonitoramento durante o período de pandemia por COVID-19.	I	C	**NOVA:** Recomendações de *experts* e sociedades.	Nova	51,52
Considerando que sintomas de COVID-19 podem mimetizar descompensação de IC, a testagem para SARS-CoV-2 com RT-PCR é recomendável tanto no cenário de pacientes atendidos em sala de emergência como em casos ambulatoriais.^46^^,^^47^ Não há evidências que contraindiquem a manutenção de iECA, BRA ou INRA em pacientes com IC que apresentem quadro de COVID-19. Durante o período infeccioso, a manutenção ou não destas medicações deve ser guiada pelo quadro clínico e/ou hemodinâmico, individualmente.^48^^–^^50^ Ferramentas virtuais e/ou remotas (telefonemas, telemonitoramento, consultas on-line, videochamadas, entre outras) podem ser utilizadas para manter o cuidado de pacientes com IC durante a pandemia por COVID-19. Estas medidas, que reduzem a circulação de pessoas e a exposição dos pacientes ao vírus, têm-se mostrado eficientes no cuidado e tendem a consolidar-se no período pós-pandêmico. No caso de pacientes em período de maior instabilidade clínica (pós-alta de episódio de descompensação ou IC de início recente) e de candidatos a terapias avançadas para IC (transplante ou dispositivos de assistência ventricular), é recomendável manter ao menos uma visita presencial em intervalos variáveis e intercalados com visitas virtuais, especialmente considerando que durante a pandemia há uma tendência à redução do número de transplantes realizados, prorrogando o tempo de espera em fila.^51^^,^^52^					

*IC: insuficiência cardíaca; iECA: inibidores da enzima de conversão de angiotensina II; BRA: bloqueadores dos receptores de angiotensina II; INRA: inibidores de neprilisina e antagonistas dos receptores de angiotensina II.*

## 6. Inovações em Insuficiência Cardíaca Avançada

### 6.1. Definição de Insuficiência Cardíaca Avançada

A história natural da IC caracteriza-se pela piora progressiva da função cardíaca e dos sintomas de IC. Apesar dos avanços no tratamento farmacológico e do impacto prognóstico dos dispositivos implantáveis como terapia de ressincronização cardíaca, pacientes com insuficiência cardíaca podem progredir para uma condição clínica denominada IC avançada, em que o tratamento tradicional não é efetivo e as terapias avançadas, tais como transplante cardíaco, suporte com dispositivo de assistência circulatória mecânica (DACM) ou cuidados paliativos são necessárias.

Embora o termo IC avançada já venha sendo usado desde 2007, atualizações recentes foram descritas visando incluir situações clínicas que também podem requerer terapias avançadas e que não foram contempladas na primeira classificação, como pacientes com ICFEp com quadro restritivo grave e não limitando apenas aos pacientes com IC com fração de ejeção gravemente reduzida.^1^^–^^3^^,^^4^ Neste cenário, a disfunção ventricular direita grave isolada e as doenças valvares graves inoperáveis, bem como anormalidades congênitas também podem ser consideradas causas de disfunção cardíaca grave (Tabela 6.1).^53^^–^^68^

**Tabela 6.1:** Critérios para a definição de insuficiência cardíaca avançada

**Critérios para a definição de IC avançada**
1. Sintomas de IC persistentes e graves (NYHA III ou IV).
2. Disfunção ventricular grave definida por: FEVE ≤ 30% ou IC direita isolada ou Alterações valvares graves não operáveis ou Anormalidades congênitas Valores de BNP ou NT-proBNP persistentemente elevados e dados mostrando disfunção diastólica grave ou anormalidades estruturais do VE, de acordo com critérios de definição de ICFEp ou ICFElr.
3. Episódios de congestão pulmonar ou sistêmica que requerem altas doses de diurético endovenoso (ou combinação de diuréticos) ou episódios de baixo débito que requerem uso de inotrópicos ou fármacos vasoativos ou arritmias malignas que causem mais que uma visita não planejada à emergência ou hospitalização nos últimos 12 meses.
4. Capacidade para o exercício gravemente reduzida, com inabilidade para o exercício ou baixa capacidade no teste de caminhada de 6 minutos (TC6min < 300m) ou VO_2_ pico (<12-14 mL.kg^-1^.min^-1^), estimado de origem cardíaca.

*Adaptado de Metra M et al.*^*65*^*Eur J Heart Fail. 2007; 9(6-7): 684-94; Metra M et al.*^*66*^*Cardiac Fail Rev. 2019; Crespo-Leiro MG et al.*^*67*^*Eur J Heart Fail. 2018; 20(11): 505-35; Trusby LK et al.,*^*68*^*JACC Heart Fail. 2020; 8(7): 523-36.*

*IC: insuficiência cardíaca; NYHA: New York Heart Association; FEVE: fração de ejeção ventricular esquerda; BNP: peptídeo natriurético do tipo B; NTproBNP: fração N-terminal do peptídeo natriurético do tipo B; ICFEp: insuficiência cardíaca com fração de ejeção preservada; ICFElr: insuficiência cardíaca com fração de ejeção levemente reduzida; TC6m: teste de caminhada de 6 minutos; VE: ventrículo esquerdo; VO_2_: consumo de oxigênio.*

Estes critérios variam de acordo com as diferentes sociedades de cardiologia, porém a presença de sintomas graves persistentes; a capacidade reduzida ao exercício; e episódios recorrentes de congestão pulmonar ou sistêmica que necessitem hospitalizações estão presentes em todas elas como descrito na Tabela 6.2.

**Tabela 6.2:** Critérios propostos por diferentes sociedades de cardiologia para identificação de pacientes com IC avançada

Critério	SBC	ACC/AHA	ESC	HFSA
Sintomas graves e persistentes apesar de terapia otimizada	✓	✓	✓	✓
Limitação funcional importante (classe funcional NYHA III ou IV)	✓	✓	✓	✓
Dispneia persistente com atividades da vida diária		✓		
Hospitalizações recorrentes	✓	✓	✓	✓
Visitas frequentes não planejadas à emergência	✓		✓	✓
Intolerância a otimização terapêutica máxima	✓	✓		✓
Lesão em órgão-alvo	✓	✓		✓
Hiponatremia persistente	✓	✓		✓
Congestão pulmonar ou sistêmica refratária a diureticoterapia	✓	✓		✓
Choques de cardiodesfibrilador implantável frequentes	✓	✓		✓
Caquexia cardíaca	✓	✓		✓
Pressão arterial sistólica frequentemente ≤ 90mmHg		✓		
Valores de BNP ou NT-proBNP persistentemente elevados	✓		✓	
Disfunção grave de VE com fração de ejeção reduzida (FEVE < 30%)	✓		✓	✓
Disfunção grave de VE com padrão ecocardiográfico pseudonormal ou restritivo	✓		✓	
Pressões de enchimento elevadas (PCP >16mmHg +/- PVC > 12mmHg)			✓	
Baixa capacidade no TC6m (< 300 m) ou VO_2_ pico < 12-14 mL.kg^-1^.min^-1^	✓		✓	✓
Dependência de inotrópicos intravenosos	✓			✓
Disfunção progressiva de VD e HP secundária	✓			✓

*Adaptado de Metra M et al.*^*65*^*Eur J Heart Fail. 2007; 9(6-7): 684-94; Metra M et al.*^*66*^*Cardiac Fail Rev 2019; Crespo-Leiro MG et al.*^*67*^*Eur J Heart Fail. 2018; 20(11): 505-35; Trusby LK et al.*^*68*^*JACC Heart Fail. 2020; 8(7): 523-36.*

*ACC/AHA: American College of Cardiology/American Heart Association CDI: cardiodesfibrilador implantável; IC: insuficiência cardíaca; BNP: peptídeo natriurético do tipo B; ESC: European Society of Cardiology; HFSA: Heart Failure Society of America; HP: hipertensão pulmonar; NYHA: New York Heart Association; NT-proBNP: fração N-terminal do peptídeo natriurético do tipo B; PCP: pressão capilar pulmonar; PVC: pressão venosa central; TC6m: teste de caminhada de 6 minutos; VD: ventrículo direito; VE: ventrículo esquerdo; VO_2_: consumo de oxigênio.*

É importante ressaltar que o reconhecimento precoce de um paciente com IC avançada é fator determinante para o seu prognóstico, uma vez que isto permitirá o encaminhamento para um centro especializado que disponha das terapias avançadas necessárias para a condução do caso.

Uma técnica mnemônica particularmente útil e que pode ajudar a identificar pacientes que precisam de encaminhamento a um especialista em IC é o *I-NEED-HELP*, que integra a história clínica, as hospitalizações e a intolerância medicamentosa, além de sintomas e a disfunção de órgão-alvo. (Tabela 6.3)

**Tabela 6.3:** Sinais de alerta no paciente com IC avançada

I	Inotrópico dependente/intolerância à terapia otimizada
N	NYHA III/IV persistente
E	Ejeção (fração) menor que 20%
E	Edema persistente, refratário a doses progressivas de diuréticos
D	Desfibrilador (choque apropriado recorrente)
H	Hospitalizações e visitas à emergência recorrentes nos últimos 12 meses
E	Elevação persistente de peptídeos natriuréticos
L	Lesão em órgão-alvo
P	Pressão arterial sistólica persistentemente menor que 90 mmHg

### 6.2. Papel do Especialista em Insuficiência Cardíaca Avançada

Com a delimitação cada vez mais clara de um perfil específico de pacientes enquadrados na definição mais contemporânea de IC avançada, cresce também a necessidade de definir a importância do papel do especialista em IC avançada nos centros especializados. Este profissional deverá ser familiarizado (e treinado) para atender potenciais cadidatos ao transplante cardíaco (e posterior seguimento) e pacientes em choque cardiogênico (CC). Ele assume a liderança na coordenação dos trabalhos envolvendo o time de choque e, portanto, está familiarizado com as diversas (e crescentes) opções de uso de assistência circulatória. Por fim, este profissional deve ser capaz de compreender o momento e as implicações de discutir cuidados paliativos e diretivas antecipadas de vontade para pacientes não elegíveis para transplante cardíaco, bem como o uso de dispositivos de longa permanência.

### 6.3. Abordagem do Paciente com Insuficiência Cardíaca Avançada (Figura 6.1)

**Figura 6.1:**
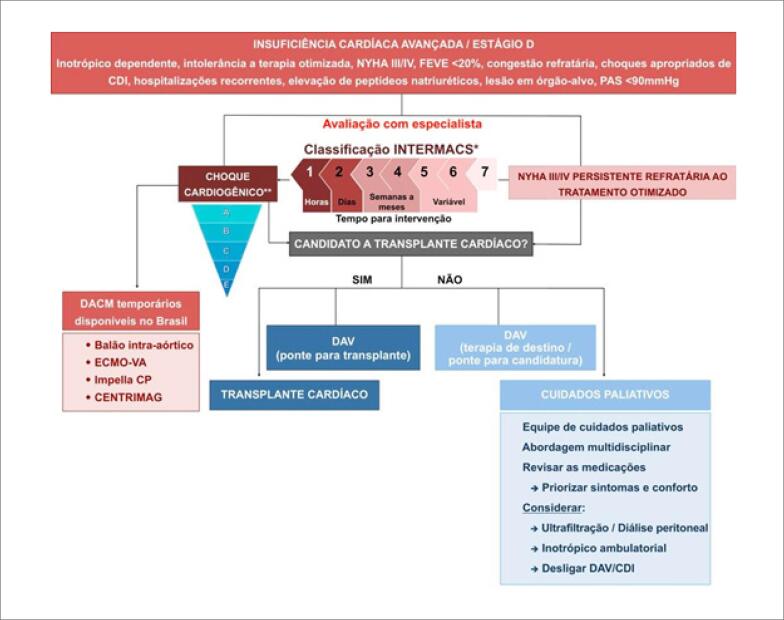
*Algoritmo de tratamento do paciente com insuficiência cardíaca avançada*.

*Classificação clínica de pacientes com insuficiência cardíaca avançada da Interagency Registry for Mechanically Assisted Circulatory Support (Intermacs), vide Diretriz Brasileira de Insuficiência Cardíaca Crônica e Aguda.^*15*^Arq Bras Cardiol. 2018; Quadro 4.6 (página 505).

**Classificação de choque cardiogênico proposta pela Society for Cardiovascular Angiography and Interventions (SCAI). Estágio A: sob risco de choque; Estágio B: início do choque; Estágio C: choque clássico; Estágio D: choque em deterioração; Estágio E: choque extremo. Adaptado de Baran DA et al.^*58*^*Catheter Cardiovasc Interv. 2019; 94(1): 29-37.*

*CDI: cardiodesfibrilador implantável; CF: classe funcional; DACM: dispositivos de assistência circulatória mecânica; DAV: dispositivo de assistência ventricular implantável; ECMO-VA: circuito de oxigenação por membrana extracorpórea venoarterial; IC: insuficiência cardíaca; NYHA: New York Heart Association.*

### 6.4. Inovações sobre o Manejo da Congestão em Pacientes com Insuficiência Cardíaca Avançada (Tabela 6.4)

**Tabela 6.4:** Monitorização ambulatorial da congestão na insuficiência cardíaca

Orientações	Classe	NE	Comentário	Tabela 2018	Ref.
Monitorização invasiva remota da congestão, através de dispositivo implantável na artéria pulmonar para reduzir hospitalizações e mortalidade em pacientes com ICFEr ambulatoriais.	IIa	B	**NOVA:** A orientação atual reflete dados de estudos randomizados pequenos e estudos de vida real, com impacto em redução de hospitalizações e mortalidade.	Nova	30,53–57
Embora poucas inovações tenham sido observadas nos últimos anos com relação ao manejo da congestão na IC avançada, algumas evidências surgiram com relação à monitorização da congestão, o que impactou no prognóstico de pacientes com IC. Neste campo, existem dados recentes sobre a monitorização não invasiva através de telemonitoramento, mostrando impacto em redução de dias perdidos com admissão hospitalar e morte por todas as causas.^30^ Também foram encontrados dados sobre monitorização invasiva através de dispositivo implantável na artéria pulmonar, CardioMEMs, que permite a transmissão de medidas de pressão arterial pulmonar (PAP) para uma central e, por conseguinte, ao médico do pacien*te. O impacto desta monitorização invasiva foi testado no estudo CHAMPION (CardioMEMS Heart Sensor Allows Monitoring of Pressure to Improve Outcomes in NYHA Class III Heart Failure Patients trial)*, que envolveu pacientes ambulatoriais com IC (NYHA III) e mostrou redução de 28% nas hospitalizações por IC. Nos pacientes que receberam ao menos duas medicações da terapia padrão para IC, a monitorização invasiva associou-se a 57% de redução de mortalidade.^53^ Esta estratégia mostrou-se eficaz e segura em estudos de “vida real”,^54^ além de custo-efetiva.^55^ Estes dados foram confirmados recentemente em estudo conduzido por centros na Europa^56^ e em estudo prospectivo multicêntrico aberto que envolveu 1.200 pacientes em CF III, que demonstrou redução significativa de hospitalização por IC com baixas taxas de complicações relacionadas com o implante do sensor no seguimento de um ano.^57^ Trata-se de uma estratégia promissora com potencial a ser acrescentada à prática clínica.					

*CF: classe funcional; IC: insuficiência cardíaca; NYHA: New York Heart Association; PAP: pressão de artéria pulmonar.*

### 6.5. Classificação Atual de Choque Cardiogênico

**Figura 6.2:**
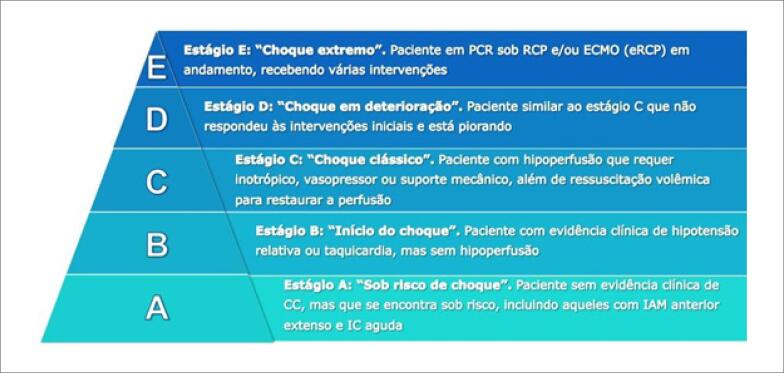
*Classificação da Society for Cardiovascular Angiography and Interventions (SCAI) para o choque cardiogênico*.

Em 2019, a *Society for Cardiovascular Angiography and Interventions (SCAI)* propôs uma nova classificação para o CC, visando facilitar a identificação das diferentes fases de deterioração clínica e a necessidade de intensificação do tratamento.^58^^,^^59^ As cinco fases dessa classificação incorporam achados de hipoperfusão tecidual e os sinais de disfunção orgânica que permitem uma definição hemodinâmica simples e maior discriminação entre os perfis INTERMACS *(Interagency Registry for Mechanically Assisted Circulatory Support)* (Figura 6.2, Tabela 6.5).

*Adaptado de: Baran DA et al.*^*58*^*SCAI clinical expert consensus statement on the classification of cardiogenic shock. Catheter Cardiovasc Interv. 2019; 94(1): 29-37.*

*CC: choque cardiogênico; ECMO: oxigenação por membrana extracorporal; IAM: infarto agudo do miocárdio; IC: insuficiência cardíaca; PCR: parada cardiorrespiratória; RCP: ressuscitação cardiorrespiratória.*

**Tabela 6.5:** Descritores das fases do choque cardiogênico: exame físico, biomarcadores e achados hemodinâmicos

Estágio do CC	Achados clínicos	Biomarcadores	Hemodinâmica
**A (sob risco)**	PVJ normal Ausculta pulmonar limpa Perfil quente e seco Pulsos periféricos cheios Estado mental preservado	Bioquímica normal Função renal normal Lactato normal	PAS ≥ 100 mmHg (ou normal para o paciente) Se CAP: • CI ≥ 2,5L/min/m^2^ • PVC < 10mmHg • SvO_2_ ≥ 65%
**B (início)**	PVJ elevada Estertores pulmonares Perfil quente e seco Pulsos periféricos cheios Estado mental preservado	Lactato normal Disfunção renal mínima BNP elevado	PAS < 90 OU PAM < 60 OU queda > 30 mmHg do basal FC ≥ 100 bpm Se CAP: CI ≥ 2,2 L/min/m^2^ SvO_2_ ≥ 65%
**C (clássico)**	***Pode incluir qualquer um dos seguintes:*** Mal-estar geral Em pânico Palidez e lividez Sobrecarga de volume Estertores pulmonares difusos Classificação de Killip 3 ou 4 BiPap ou ventilação mecânica Extremidades frias e pegajosas Alteração aguda do estado mental Débito urinário < 30 mL/h	Pode incluir qualquer um dos seguintes: Lactato ≥ 2 mmol/L Creatinina dobrando ou queda > 50% na TFG Provas hepáticas alteradas BNP elevado	Pode incluir qualquer um dos seguintes: PAS < 90 OU PAM < 60 OU queda >30 mmHg do basal. Vasopressores e/ou dispositivos para manter a PA CAP: • CI < 2,2L/min/m^2^ • PCP > 15 mmHg • PVC/PCP ≥ 0,8 • PAPi < 1,85 • CPO ≤ 0,6W
**D (deterioração)**	Pode incluir qualquer um dos achados do estágio C	Pode incluir qualquer um dos achados do estágio C, em deterioração	Pode incluir qualquer um dos achados do estágio C e: Múltiplos vasopressores e/ou dispositivos de assistência circulatória mecânica para manter perfusão
**E (extremo)**	Pulsos pouco palpáveis Colapso circulatório Sob ventilação mecânica Desfibrilador em uso	PCR (modificador – A*) pH ≤ 7,2 Lactato ≥ 5 mmol/L	PAS inaudível / PCR TVSP ou TV/FV refratária Hipotensão apesar do suporte máximo

* *O modificador (A) é aplicado para descrever pacientes que tiveram uma parada cardíaca independentemente da duração.*

*Adaptado de Baran DA et al.*^*58*^*SCAI clinical expert consensus statement on the classification of cardiogenic shock. Catheter Cardiovasc Interv. 2019; 94(1):29-37.*

*BiPap: ventilação por dois níveis de pressão positiva; BNP: peptídeo natriurético do tipo-B; CAP: cateter de artéria pulmonar; CC: choque cardiogênico; IC: índice cardíaco; CPO: poder cardíaco, do inglês cardiac power output; FC: frequência cardíaca; FV: fibrilação ventricular; IAM: infarto agudo do miocárdio; IC: insuficiência cardíaca; PAM: pressão arterial média; PAPi: índice de pulsatilidade da artéria pulmonar; PAS: pressão arterial sistólica; PCP: pressão de capilar pulmonar; PVC: pressão venosa central; PVJ: pulso venoso jugular; SvO_2_: saturação venosa mista de oxigênio; TFG: taxa de filtração glomerular; TV: taquicardia ventricular; TVSP: taquicardia ventricular sem pulso.*

O estágio A inclui pacientes sob risco de choque cardiogênico, enquanto os estágios B a E descrevem fases progressivas do choque cardiogênico convencional. A diferença entre os estágios B e C é a presença de hipoperfusão, que está presente nos estágios C e superiores. O estágio D indica que as medidas de manejo inicial do choque cardiogênico não foram suficientes para restaurar a estabilidade hemodinâmica ou a perfusão tecidual após pelo menos 30 minutos de observação, enquanto o estágio E caracteriza casos extremos, no qual os pacientes se apresentam hemodinamicamente instáveis e frequentemente em colapso circulatório. Pacientes em estágios SCAI D e E apresentam maior mortalidade e podem se beneficiar da transferência precoce para centros especializados, capazes de oferecer modalidades avançadas de suporte circulatório.^59^

### 6.6. Aplicabilidade do Cateter de Artéria Pulmonar na Insuficiência Cardíaca Avançada (Tabela 6.6)

**Tabela 6.6:** Recomendações de uso de cateter de artéria pulmonar em pacientes com insuficiência cardíaca avançada

Recomendações	Classe	NE	Comentário	Tabela 2018	Ref.
Em pacientes com IC avançada, candidatos a transplante cardíaco ou suporte circulatório mecânico.	I	B	Recomendação de 2018 mantida.	Item 2.2.6. (página 495)	Vide 2018
Para auxiliar no tratamento e suporte hemodinâmico de pacientes com IC refratária ao tratamento padrão ou em pacientes com choque cardiogênico.	IIa	B	**MODIFICADO:** Novas evidências respaldam a mudança de classe de recomendação.	Item 2.2.6. (página 495)	60–61
O uso do cateter de artéria pulmonar (CAP) na monitorização hemodinâmica de pacientes hospitalizados com IC refratária permanece controverso.^62^^,^^63^ Em 2005, o estudo *ESCAPE (Evaluation Study of Congestive Heart Failure and Pulmonary Artery Catheterization Effectiveness)* não mostrou benefício do uso rotineiro do CAP no manejo de pacientes com IC descompensada sem CC.^64^ Entretanto, avanços recentes no campo dos dispositivos de assistência circulatória mecânica (DACM) têm favorecido a criação de algoritmos para o manejo do CC guiado por parâmetros do CAP. O reconhecimento precoce, a identificação do subtipo de choque e a compreensão do impacto esperado de cada tipo de dispositivo sobre parâmetros hemodinâmicos como débito cardíaco, pressão capilar pulmonar (PCP), pressão venosa central (PVC) e pressão arterial média (PAM) permite a escolha do DACM mais adequado para cada estágio do CC (Figura 6.1). Além disso, as informações obtidas através do CAP auxiliam na caracterização fenotípica do CC em choque predominantemente esquerdo (CPO < 0,6 W, PAPi > 1, PVC < 15 mmHg e PCP > 15 mmHg), direito (CPO < 0,6 W, PAPi <1, PVC >15mmHg e PCP <15 mmHg) ou biventricular (CPO <0,6 W, PAPi <1, PVC >15 mmHg e PCP >15 mmHg).^60^^,^^65^^–^^68^ Recentemente, em um dos primeiros estudos do *Cardiogenic Shock Working Group* (CSWG), Garan et al.^61^ avaliaram a associação entre o manejo do CC guiado por parâmetros do CAP e a mortalidade hospitalar em 1.414 pacientes com CC, a maioria com indicação de DACM e em estágio D da classificação SCAI. O manejo do CC guiado por parâmetros do CAP obtidos antes do implante de DACM foi associado a redução significativa de mortalidade, principalmente nos estágios mais avançados de CC (estágios D ou E da classificação SCAI).^61^ É importante enfatizar que o CAP é uma ferramenta diagnóstica, não terapêutica, e sua efetividade dependerá de decisões clínicas tomadas pelo time de profissionais envolvidos com o manejo do CC.					

*CAP: cateter de artéria pulmonar; CC: choque cardiogênico; CPO: poder cardíaco, do inglês cardiac power output; DACM: dispositivos de assistência circulatória mecânica; IC: insuficiência cardíaca; PAM: pressão arterial média; PAP: índice de pulsatilidade da artéria pulmonar; PCP: pressão de capilar pulmonar; PVC: pressão venosa central.*

### 6.7. Inovações com Relação aos Dispositivos de Assistência Circulatória de Curta Duração na Insuficiência Cardíaca Avançada (Tabela 6.7)

**Tabela 6.7:** Recomendações para descompressão do ventrículo esquerdo em pacientes com oxigenação por membrana extracorpórea (ECMO)

Recomendações	Classe	NE	Comentário	Tabela 2018	Ref.
Considerar estratégias para descompressão do ventrículo esquerdo em pacientes com suporte de assistência circulatória mecânica por ECMO venoarterial periférica e evidência de distensão ventricular associada à hipocontratilidade acentuada e à congestão pulmonar.	IIa	C	**NOVA:** A recomendação atual reflete dados de estudos observacionais e metanálises.	Nova	69–73
O uso de oxigenação por membrana extracorpórea (ECMO) venoarterial periférica é caracterizado pelo aumento na pós-carga do VE causado pelo fluxo sanguíneo da cânula de retorno arterial, que pode agravar a hipocontratilidade cardíaca gerando distensão ventricular e congestão pulmonar. Em muitos casos, a redução no fluxo da ECMO combinada à terapia inotrópica pode ser suficiente para descomprimir o VE.^74^ No entanto, em casos refratários, outros métodos de descompressão podem ser usados, incluindo septostomia atrial; implante cirúrgico de cateter transapical; descompressão percutânea da artéria pulmonar pela veia jugular; e dispositivo de assistência circulatório mecânica (DACM) como balão intra-aórtico (BIA), Impella^®^, ou CentriMag^®^. Em estudos observacionais, a descompressão do VE se associou à redução de mortalidade, à maior recuperação miocárdica e ao menor tempo de desmame da ECMO em pacientes com CC tratados com ECMO venoarterial periférica.^69^^–^^72^ Cada técnica de descompressão apresenta riscos inerentes que devem ser considerados individualmente de acordo com a etiologia da doença de base, as limitações do sítio de acesso, a presença de coagulopatias, a disponibilidade dos DACMs e a experiência de cada centro.^75^ Apesar das limitações conhecidas, o BIA permanece o dispositivo mais utilizado, com uma metanálise recente sugerindo menor risco de complicações como acidente vascular cerebral, isquemia periférica e hemólise, da descompressão por BIA em comparação com outros métodos, às custas do aumento de sangramento.^73^ No entanto, nenhum ensaio clínico randomizado foi realizado até o momento para estabelecer o método ideal de descompressão do VE, e estudos prospectivos são necessários. Também não há consenso se a descompressão do VE deva ser realizada preventivamente ou como medida de resgate. Algumas indicações reconhecidas para descompressão do VE são: pressão capilar pulmonar (PCP) elevada, VE distendido e hipocontrátil ou com evidência ecocardiográfica de estase sanguínea, diminuição da abertura valva aórtica durante o ciclo cardíaco, hipoxemia, edema pulmonar progressivo e arritmia ventricular refratária.					

*CC: choque cardiogênico; DACM: dispositivo de assistência circulatório mecânica; ECMO: oxigenação por membrana extracorpórea; PCP: pressão capilar pulmonar; VE: ventrículo esquerdo.*

### 6.8. Inovações com Relação aos Cuidados Paliativos na Insuficiência Cardíaca Avançada (Tabela 6.8)

**Tabela 6.8:** Uso de inotrópicos intravenosos em regime ambulatorial para pacientes com insuficiência cardíaca avançada, não elegíveis para transplante cardíaco ou dispositivos de assistência circulatória mecânica

Recomendações	Classe	NE	Comentário	Tabela 2018	Ref.
Terapia inotrópica ambulatorial intravenosa contínua como tratamento paliativo para o controle de sintomas em pacientes com IC avançada, que não são elegíveis para dispositivos de assistência circulatória mecânica ou transplante cardíaco.	IIb	C	**NOVA:** A recomendação atual reflete dados de estudos com limitações de desenho e execução.	Nova	76–78
Uso intermitente de inotrópico ou inodilatador para melhora dos sintomas em pacientes com IC avançada ou para paliação em pacientes sem outras opções de terapias avançadas.	IIb	B	**NOVA:** Novas evidências de ECR e metanálise de moderada qualidade respaldam a recomendação.	Nova	79
A qualidade da evidência avaliando riscos e benefícios da terapia paliativa com inotrópico intravenoso em regime ambulatorial para pacientes com IC avançada é limitada e composta principalmente por estudos observacionais e sem um grupo controle. Metanálises de pequenos estudos randomizados controlados e estudos observacionais heterogêneos sugerem um potencial benefício clínico da terapia inotrópica ambulatorial contínua ou intermitente em pacientes com IC avançada, que não são elegíveis para DACM ou transplante cardíaco.^76^^–^^78^ Dentre os benefícios, destacam-se o alívio sintomático e a redução nas taxas de readmissão hospitalar. Entretanto, a necessidade de um cateter central para infusão contínua de inotrópicos está associada a maiores cuidados especiais e ao risco de infecções. O ensaio piloto *LION-HEART (Efficacy and safety of intermittent intravenous outpatient administration of levosimendan in patients with advanced heart failure)* randomizou 69 pacientes com IC avançada para receber placebo ou levosimendana intermitente, na dose de 0,2 ¼g/kg/min por 6 horas, a cada 2 semanas e em 12 semanas demonstrou benefício do inotrópico com relação à redução de concentrações plasmáticas de NT-proBNP, escores de qualidade de vida e readmissões hospitalares, sem diferença nas taxas de eventos adversos entre os grupos.^79^ Até o momento, não há estudos de custo-efetividade avaliando o impacto da infusão ambulatorial de inotrópicos como terapia paliativa para pacientes com IC avançada.					

*ECR: estudo controlado randomizado; IC: insuficiência cardíaca; NT: ProBNP-fração N-terminal do peptídeo natriurético do tipo B.*

## 7. Tratamento da Insuficiência cardíaca com fração de ejeção reduzida (ICFEr)

### 7.1. Estratégias Farmacológicas Previamente Consolidadas para Tratamento da Insuficiência Cardíaca com fração de Ejeção Reduzida (ICFEr) (Tabela 7.1)

**Tabela 7.1:** Recomendações para tratamento farmacológico da insuficiência cardíaca com fração de ejeção reduzida (ICFEr) previamente consolidadas em 2018

Recomendações	Classe	NE	Comentário	Tabela 2018	Ref.
Bisoprolol, carvedilol e succinato de metoprolol para disfunção de VE sintomática para reduzir morbidade e mortalidade.	I	A	Recomendação de 2018 mantida.	Item 7.2 (página 457)	Vide 2018
iECA para disfunção de VE sintomática para reduzir morbidade e mortalidade.	I	A	Recomendação de 2018 mantida.	Item 7.1 (página 456)	Vide 2018
BRA para disfunção de VE sintomática (nos intolerantes a iECA por tosse/angioedema) para reduzir morbidade e mortalidade.	I	A	Recomendação de 2018 mantida.	Item 7.1 (página 456)	Vide 2018
Antagonista dos receptores mineralocorticoides para disfunção de VE sintomática, associada ao tratamento padrão com iECA/BRA/INRA e BB, para reduzir morbidade e mortalidade.	I	A	**MODIFICADO:** O uso de antagonista dos receptores mineralocorticoides justifica-se em pacientes em uso iECA/BRA ou INRA.	Item 7.3 (página 457)	80–84
Sacubitril-valsartana, em substituição ao iECA (ou BRA), para disfunção de VE sintomática, já em uso de terapêutica otimizada e com terapia tripla para reduzir morbidade e mortalidade.	I	B	Recomendação de 2018 mantida.	Item 7.4 (página 458)	Vide 2018
Associação de hidralazina e nitrato para disfunção sistólica sintomática em classe funcional II-IV (NYHA) com contraindicação à iECA/BRA (insuficiência renal e/ou hipercalemia) independentemente de raça ou para pacientes negros autodeclarados com disfunção sistólica sintomática em NYHA III-IV, apesar de terapêutica otimizada.	I	B	Recomendação de 2018 mantida.	Item 7.7 (página 459)	Vide 2018
Ivabradina para disfunção de VE sintomática, em paciente com terapêutica otimizada, em ritmo sinusal e com FC maior que 70 bpm para redução de hospitalização, morte cardiovascular e morte por IC.	IIA	B	Recomendação de 2018 mantida.	Item 7.5 (página 458)	Vide 2018
Digoxina para disfunção de VE sintomática, apesar de terapêutica otimizada com terapia tripla, para reduzir sintomas e hospitalizações.	IIA	B	Recomendação de 2018 mantida.	Item 7.6 (página 458)	Vide 2018
Diurético de alça para controle de congestão.	I	C	Recomendação de 2018 mantida.	Item 7.7 (página 459)	Vide 2018
Diurético tiazídico, associado ao diurético de alça para congestão persistente.	I	C	Recomendação de 2018 mantida.	Item 7.7 (página 459)	Vide 2018
Nas últimas décadas, os avanços no tratamento farmacológico e no uso de dispositivos implantáveis trouxeram mudanças no prognóstico de pacientes com ICFEr.^80^^–^^91^ No entanto, ainda existe risco residual e alta morbimortalidade mesmo após tratamento clínico otimizado já ter sido instituido. Tratamento clínico otimizado adicional. Nesta nova era, medicações que agem em diferentes mecanismos fisiopatológicos da IC surgem para complementar a ação exercida sobre o sistema neuro-humoral. É importante ressaltar que os benefícios observados com as novas medicações ocorreram em adição à terapia padrão otimizada, o que reforça a necessidade de manutenção da terapia tripla, que inclui betabloqueadores, bloqueadores do sistema renina-angiotensina-aldosterona (SRAA) e antagonistas mineralocorticoides. Uma vez instituída a terapia tripla e adicionadas as novas terapias que demonstraram benefício em redução de mortalidade cardiovascular, morte geral e hospitalização por IC, podemos também associar medicações que tiveram impacto em morbidade. A escolha destas terapias adicionais deve levar em consideração o perfil de cada paciente.					

*iECA: inibidores da enzima de conversão de angiotensina II; BRA: bloqueadores dos receptores de angiotensina II; INRA: inibidores da neprilisina e antagonista do receptor de angiotensina II; VE: ventrículo esquerdo; FC: frequência cardíaca; ICFEr: insuficiência cardíaca com fração de ejeção reduzida; IC: insuficiência cardíaca; SRAA: sistema renina-angiotensina-aldosterona.*

### 7.2. Sacubitril-Valsartana (Tabela 7.2)

**Tabela 7.2:** Recomendações para o uso de sacubitril-valsartana em pacientes com insuficiência cardíaca com fração de ejeção reduzida (ICFEr)

Recomendações	Classe	NE	Comentário	Tabela 2018	Ref.
Sacubitril-valsartana, em substituição ao iECA/BRA para disfunção de VE sintomática, já em uso de terapêutica otimizada com terapia tripla para reduzir morbidade e mortalidade.	I	B	Recomendação de 2018 mantida.	Item 7.4 (página 458)	Vide 2018
Sacubitril-valsartana, como início de tratamento na IC crônica sintomática, pode ser considerado no lugar de iECA ou BRA.	IIa	C	**NOVA:** Análise de subgrupos de estudos randomizado e não randomizado mostram a segurança de uso em pacientes virgens de uso de iECA/BRA.	Nova	84,92,93
Sacubitril-valsartana, em lugar de iECA/BRA, pode ser considerado em pacientes hospitalizados com IC descompensada.	IIa	B	**NOVA:** Estudo randomizado usando desfecho substituto (redução de biomarcadores) respalda esta nova recomendação.	Nova	84,92,94
O estudo *PARADIGM-HF* (*Prospective Comparison of ARNi with ACE-I to Determine Impact on Global Mortality and Morbidity in Heart Failure*) investigou, em pacientes com ICFEr, os efeitos sobre morbidade e mortalidade da atenuação da ação deletéria da Angiotensina II associada à potencialização do efeito protetor dos peptídeos natriuréticos endógenos pela inibição da neprilisina (enzima que degrada o BNP), utilizando uma nova classe de fármacos, os inibidores da neprilisina e dos receptores da Angiotensina II (INRAs), cuja molécula atualmente disponível é o sacubitril-valsartana, em comparação com enalapril.^83^ Foram investigados 8.442 pacientes com ICFEr sintomática ambulatorial com terapia clínica otimizada e que persistiam com FEVE ≤ 40%, níveis elevados de peptídeos natriuréticos plasmáticos e clearance de creatinina estimado ≥ 30 mL/min/1,73 m^2^. Nesta população, sacubitril-valsartana foi superior ao enalapril, associado à redução de 21% das internações por piora da IC, de 20% na mortalidade cardiovascular, de 20% na morte súbita e 16% na mortalidade geral. Com base nos resultados do *PARADIGM-HF*, recomenda-se a troca deiIECA/BRA para o sacubitril-valsartana nos pacientes com ICFEr que persistem sintomáticos, mesmo após o emprego de doses otimizadas dos bloqueadores neuro-hormonais. Mais recentemente, o estudo *PIONEER-HF (Angiotensin–Neprilysin Inhibition in Acute Decompensated Heart Failure)* comparou sacubitril-valsartana (n = 440) com enalapril (n = 441) em pacientes internados por IC descompensada, tendo como desfecho primário o tempo médio de mudança proporcional na concentração de NT-proBNP desde o início até a 4ᵃ e a 8ᵃ semanas.^84^ Os resultados mostraram uma redução significativa de NT-proBNP em maior grau com sacubitril-valsartana do que com enalapril, sendo esta redução observada já na primeira semana de tratamento e independentemente da história prévia de IC ou do uso prévio de iECA ou BRA.^94^ Os efeitos colaterais, hipercalemia, disfunção renal e hipotensão foram semelhantes em ambos os grupos. Em uma análise aberta, após o término das 8 semanas (*Pioneer-HF extended*) em que todos os pacientes receberam o sacubitril-valsartana por adicionais 4 semanas, observou-se uma queda adicional significante do NT-proBNP no grupo enalapril que passou a usar sacubitril-valsartana.^92^ Outro estudo prospectivo, observacional, o estudo *TRANSITION (Initiation of sacubitril/valsartan in haemodynamically stabilised heart failure patients in hospital or early after discharge)*,^93^ iniciou sacubitril-valsartana em 1.002 pacientes durante a hospitalização por IC descompensada ou logo após a alta hospitalar e mostrou ser seguro e com boa tolerância, com metade dos pacientes atingindo a dose-alvo em 10 semanas, com poucos eventos adversos.^93^ A partir dos resultados desses estudos, que sugerem ser seguro o uso de sacubitril-valsartana em pacientes internados por IC aguda descompensada após estabilização clínica, e a partir da extrapolação dos benefícios demonstrados no estudo *PARADIGM-HF*, o sacubitril- valsartana pode ser considerado, em lugar de iECA/BRA, para tratamento de pacientes hospitalizados com IC descompensada. Os resultados desses estudos recentes também indicam a segurança e tolerabilidade do início de tratamento com sacubitril-valsartana, ao invés de iECAs/BRAs, em pacientes com ICFEr nova, que compuseram 34% da casuística do estudo *Pioneer-HF* e 29% dos pacientes no estudo *TRANSITION*.^84^^,^^92^^,^^93^ Em conjunto, estes dados sugerem que o início de sabubitril/valsartana para pacientes sem tratamento prévio com iECA/BRA e durante episódios de descompensação de IC seja aceitável e seguro. Dados a respeito deste tipo de intervenção em longo prazo e com desfechos, como mortalidade, ainda não estão disponíveis.					

*iECA: inibidores da enzima de conversão de angiotensina II; BRA: bloqueadores dos receptores de angiotensina II; INRA: inibidores da neprilisina e antagonista do receptor de angiotensina II; IC: insuficiência cardíaca; FEVE: fração de ejeção ventricular esquerda; VE: ventrículo esquerdo; FC: frequência cardíaca; ICFEr: insuficiência cardíaca com fração de ejeção reduzida; SRAA: sistema renina-angiotensina-aldosterona.*

### 7.3. Inibidores de SGLT2 (Tabela 7.3)

**Tabela 7.3:** Recomendações para o uso de Inibidores de SGLT2 no tratamento de pacientes com Insuficiência cardíaca com fração de ejeção reduzida (ICFEr)

Recomendações	Classe	NE	Comentário	Tabela 2018	Ref.
Inibidores de SGLT2 (dapagliflozina ou empagliflozina) em pacientes com ICFEr sintomáticos diabéticos ou não já com dose máxima otimizada tolerada de betabloqueador, antagonista da aldosterona, iECA/BRA ou INRA para reduzir desfechos cardiovasculares e progressão da disfunção renal.	I	A	**NOVA:** Os iSGLT2 são úteis para redução de morte cardiovascular, e hospitalização por insuficiência cardíaca.	Nova	95–98
No *DAPA-HF* (*Dapagliflozin and Prevention of Adverse Outcomes in Heart Failure*), 4.744 pacientes com ICFEr foram randomizados para receber dapagliflozina ou placebo além da terapia-padrão, sendo 41,8% com DM2.^95^ O desfecho primário de morte cardiovascular ou agravamento da IC foi significativamente menor no grupo dapagliflozina (26% de redução). Quando analisados separadamente, houve redução significativa tanto na morte cardiovascular (18% de redução) quanto na piora da IC (30% de redução), independentemente de DM2. Tais resultados revelam uma nova terapia para IC, já aprovada para essa finalidade. O *EMPEROR-Reduced* (*Empagliflozin Outcome Trial in Patients with Chronic Heart Failure and a Reduced Ejection Fraction*) avaliou a empagliflozina *versus* placebo, além da terapia padrão, em 3.730 pacientes com ICFEr, sendo 50,2% com DM2.^96^ Os pacientes apresentavam IC mais grave do que aqueles no *Dapa-HF*, com média de FEVE de 27% contra 31%, sendo que mais de 70% dos pacientes tinham FEVE menor que 30%, além de maior nível mediano de NT-proBNP (1.907 *versus* 1.437 pg/mL). Houve redução de 25% no desfecho primário de morte cardiovascular ou hospitalização por insuficiência cardíaca (HIC) em favor da empagliflozina. Quando analisados separadamente, não houve redução de morte cardiovascular, resultado diferente daquele observado no *Dapa-HF*. Novamente, o benefício foi visto independentemente da presença de DM2. Esses dados confirmam os resultados do *DAPA-HF* e reforçam a justificativa para o uso de inibidores do cotransportador de sódio e glicose 2 (iSGLT2) em pacientes com ICFEr para redução dos sintomas, melhora da qualidade de vida, redução do risco de hospitalização e morte cardiovascular. A metanálise utilizando os resultados do *DAPA-HF* e do *EMPEROR-Reduced*, envolvendo 8.474 pacientes, mostrou redução de 13% na morte por todas as causas (HR combinado 0,87, IC de 95% 0,77-0,98; p = 0,018) e redução de 14% nas mortes por doenças cardiovasculares (0,86, IC de 95% 0,76 - 0,98; p = 0,027).(94) O uso dos iSGLT2 foi acompanhado por uma redução relativa de 26% no risco combinado de morte cardiovascular ou primeira hospitalização por IC (0,74,0,68–0, 82; p < 0,0001), e por uma redução de 25% no composto de hospitalizações recorrentes por IC ou morte cardiovascular (0,75, 0,68–0,84; p < 0,0001).O risco do desfecho renal composto também foi reduzido (0,62, 0,43–0,90; p = 0,013). A subanálise do *DAPA-HF* avaliou a eficácia e segurança da dapagliflozina em pacientes com ICFEr, de acordo com a taxa de filtração glomerular (TFG) basal, bem como os efeitos da dapagliflozina na TFG após a randomização. O efeito da dapagliflozina nos desfechos primários (morte CV ou piora da IC) e secundários não foi alterado pela TFG (<60 e ≥ 60 mL/min/1,73m^2^). Desfecho composto renal pré-especificado (redução sustentada >50% da TFG, doença renal terminal ou morte renal) também foi analisado, juntamente com a piora da TFG ao longo do estudo. Embora dapagliflozina não tenha reduzido o desfecho renal composto (RR = 0,71, IC 95% 0,44-1,16, p = 0,17), a taxa de piora da TFG foi menor com a dapagliflozina (-1,09) *versus* placebo (-2,87), p < 0,001, em pacientes com ou sem DM2 (p de interação = 0,92).^95^ No estudo *EMPEROR-Reduced*, a taxa anual de redução da TFG foi menor com a empagliflozina do que com o placebo (-0,55 *versus* −2,28 mL/min/1,73 m^2^ por ano, p < 0,001) e os pacientes tratados com empagliflozina tiveram menor risco de desfechos renais sérios, independentemente da presença ou ausência de DM2.^96^ Os dados da subanálise do *DAPA-HF* e do *EMPEROR-Reduced* sugerem que o uso dos inibidores de SGLT2 é seguro em pacientes com ICFEr e pacientes com alteração da TFG, independentemente da presença de DM2.					

*BRA: bloqueadores dos receptores de angiotensina II; DM2: diabetes tipo 2; FEVE: fração de ejeção ventricular esquerda; IC: insuficiência cardíaca; ICFEr: insuficiência cardíaca com fração de ejeção reduzida; iECA: inibidores da enzima de conversão de angiotensina II; INRA: inibidores da neprilisina e antagonista do receptor de angiotensina II; iSGLT2: inibidores do cotransportador de sódio e glicose 2; TFG: taxa de filtração glomerular.*

### 7.4. Tratamento de Comorbidades na Insuficiência Cardíaca com fração de Ejeção Reduzida

#### 7.4.1. Diabetes Tipo 2 (Tabela 7.4)

**Tabela 7.4:** Recomendações para o uso de inibidores de SGLT2 na prevenção de hospitalização por insuficiência cardíaca em pacientes diabéticos tipo 2

Recomendações	Classe	NE	Comentário	Tabela 2018	Ref.
Inibidores de SGLT2 (canagliflozina, dapagliflozina ou empagliflozina) para prevenção de hospitalização por IC em pacientes com diabetes tipo 2 e que apresentam fatores de risco cardiovasculares para aterosclerose ou doença cardiovascular aterosclerótica estabelecida.	I	A	**NOVA:** Os ISGLT2 são úteis para reduzir a hospitalização por insuficiência cardíaca em pacientes DM2.	Item 5.2 (página 451)	99–101
Inibidores de SGLT2 (dapagliflozina ou empagliflozina) como medicação antidiabética inicial associada ou não a metformina em pacientes com ICFEr.	I	A	**NOVA:** Os iSGLT2 são úteis para tratamento do diabetes e na redução de evento cardiovascular e renal.	Nova	102
Os benefícios dos iSGLT2 em pacientes diabéticos tipo 2 (DM2) foram descritos pela primeira vez no estudo *EMPA-REG OUTCOME (Empagliflozin, Cardiovascular Outcomes, and Mortality in Type 2 Diabetes)*, publicado em 2015, que avaliou a empagliflozina em pacientes com DM2, doença cardiovascular estabelecida e recebendo tratamento usual.^99^ Entre os que receberam o fármaco, houve redução significativa dos principais eventos cardiovasculares adversos (MACE = morte CV, IM não fatal ou AVC não fatal) (HR: 0,86 (IC] 95%: 0,74-0,99), e uma surpreendente redução na hospitalização por insuficiência cardíaca (HIC) (HR: 0,65 (IC 95%: 0,50-0,85). O *CANVAS (Canagliflozin and Cardiovascular and Renal Events in Type 2 Diabetes)*, publicado em 2017, avaliou a canagliflozina em pacientes com DM2 com alto risco para eventos cardiovasculares e recebendo tratamento usual. Houve redução no desfecho primário combinado (MACE = morte CV, IM não fatal ou AVC não fatal) e redução de HIC de 33 % (HR = 0,67, IC 95%: 0,52-0,87) bem como dos eventos renais combinados.^100^ O estudo *DECLARE–TIMI 58 (Dapagliflozin and Cardiovascular Outcomes in Type 2 Diabetes)* avaliou a dapagliflozina em pacientes com DM2 e doença aterosclerótica estabelecida ou múltiplos fatores de risco para doença aterosclerótica e recebendo tratamento usual. Não houve redução no desfecho primário combinado (MACE = morte CV, infarto do miocárdio ou AVC). Observou-se a redução no desfecho combinado de mortalidade cardiovascular e HIC de 17%, e de 27% (HR: 0,73 (IC 95%: 0,61-0,88) para HIC.^101^ Recentemente, o estudo *VERTIS VC (Cardiovascular Outcomes with Ertugliflozin in Type 2 Diabetes)* avaliou a ertugliflozina (ainda não comercializada no Brasil) em pacientes com DM2, doença cardiovascular estabelecida e recebendo tratamento usual. Não houve redução no desfecho primário combinado (MACE = morte CV, infarto do miocárdio ou AVC). Foi observado, no entanto, redução de 30% na HIC.^100^ Tomados em conjunto, os dados disponíveis demonstram a eficácia dos iSGLT2 na redução da incidência de hospitalização por IC em grupos de pacientes com DM2.^102^					

*AVC: acidente vascular cerebral; DM2: diabetes tipo 2; HIC: hospitalização por insuficiência cardíaca; IM: infarto do miocárdio; iSGLT2: inibidores do cotransportador de sódio e glicose 2.*

#### 7.4.2. Disfunção Renal (Tabela 7.5)

**Tabela 7.5:** Recomendações para o uso de inibidores de SGLT2 na prevenção de piora da função renal em pacientes com insuficiência cardíaca com fração de ejeção reduzida (ICFEr)

Recomendação	Classe	NE	Comentários	Tabela 2018	Ref.
Inibidores de SGLT2 (dapagliflozin ou empagliflozin) em pacientes com ICFEr para a prevenção da redução da função renal em pacientes com e sem diabetes, com TGF ≥ 20 mL/min/1,73 m^2^.	IIa	A	**NOVA:** Os iSGLT2 são úteis para reduzir a piora progressiva da função renal na ICFEr.	Nova	95, 96, 98, 104
No estudo *EMPEROR-Reduced* a taxa anual de redução da TFG foi menor com a empagliflozina do que com o placebo (-0,55 *versus* −2,28 mL/min/1,73 m^2^ por ano, p < 0,001) e os pacientes tratados com empagliflozina tiveram menor risco de desfechos renais sérios, independentemente da presença ou ausência de DM2.^96^ Subanálise do *DAPA-HF* avaliou a eficácia e segurança da dapagliflozina em pacientes com ICFEr, de acordo com a taxa de filtração glomerular (TFG) basal, bem como os efeitos da dapagliflozina na TFG após a randomização.^98^ No estudo *DAPA-HF*, a dapagliflozina não reduziu o desfecho renal composto (RR = 0,71, IC 95% 0,44-1,16, p = 0,17).^95^ Entretanto, em uma subanálise, a taxa de piora da TFG foi menor com a dapagliflozina (-1,09) *versus* placebo (-2,87), p < 0,001, em pacientes com ou sem DM2. O estudo *DAPA-CKD* (*Dapagliflozin in Patients with Chronic Kidney Disease*) randomizou 4.304 pacientes com doença renal crônica, com TFG de 25-75 mL/min/73 m^2^ e relação albumina-creatinina urinária de 200-5.000. A dapagliflozina reduziu o desfecho primário (composto por redução sustentada da TFG em pelo menos 50%, doença renal terminal ou morte por causas renais ou CV) (9,2% com dapagliflozina x 14,5% com placebo; [RR = 0,61, IC = 9%, 0,51-0,72; p < 0,001]. Morte ocorreu em 101 (4,5%) do grupo dapagliflozina x 146 (6,8%) grupo placebo (RR = 0,69, IC = 95% 0,53-0,88, p = 0,004). A dapagliflozina reduziu a morte cardiovascular ou a hospitalização por insuficiência cardíaca (0,67, 0,40-1,13 *versus* 0,70, 0,52-0,94, respectivamente, p de interação = 0,88). Os resultados foram consistentes, em pacientes com ou sem DM2.^104^ Os dados do *EMPEROR-Reduced*, *DAPA-CKD* e da subanálise do *DAPA-HF* sugerem que o uso dos inibidores de SGLT2 é seguro com ICFEr e com alteração da TFG, independentemente da presença de DM2. Também demonstram que os ISGLT2 podem reduzir a piora da função renal em pacientes com ICFEr.					

*DM2: diabetes tipo 2; ICFEr: insuficiência cardíaca com fração de ejeção reduzida; iSGLT2: inibidores do cotransportador de sódio e glicose 2; TFG: taxa de filtração glomerular.*

#### 7.4.3. Deficiência de Ferro (Tabela 7.6)

**Tabela 7.6:** Recomendações para uso de ferro intravenoso em pacientes com insuficiência cardíaca com fração de ejeção reduzida (ICFEr)

Recomendação	Classe	NE	Comentários	Tabela 2018	Ref.
Reposição intravenosa de carboximaltose férrica em pacientes com ICFEr e deficiência de ferro (nível ferritina sérica menor que 100 ng/mL ou entre 100-299 ng/mL com saturação de transferrina menor que 20%), mesmo na ausência de anemia para aumentar capacidade para o exercício, qualidade de vida e reduzir a hospitalização.	IIa	A	Recomendação de 2018 mantida.	Item 11.11 (página 470)	Vide 2018
Reposição intravenosa de carboximaltose férrica em pacientes com ICFEr, admitidos por IC descompensada com deficiência de ferro (ferritina sérica menor que 100 ng/mL ou entre 100-299 ng/mL, associada à saturação de transferrina menor que 20%) após a estabilização clínica para reduzir readmissão hospitalar.	IIa	B	**NOVA:** Estudo randomizado multicêntrico respalda esta recomendação	Nova	105
Em pacientes com IC crônica e deficiência de ferro, o uso de carboximaltose férrica intravenosa demonstrou melhora dos sintomas, da qualidade de vida e redução de hospitalizações em estudos randomizados e metanálises prévias.^106^^–^^108^ Recentemente, o estudo *AFFIRM-AHF (Ferric carboxymaltose for iron deficiency at discharge after acute heart failure)*, randomizado, placebo-controlado e multicêntrico avaliou o efeito da carboximaltose férrica intravenosa em 1.132 com ICFEr e deficiência de ferro (estáveis após episódio de descompensação da IC e com deficiência de ferro - ferritina < 100 ng/mL ou ferritina sérica entre 109-299 ng/mL associada a saturação de transferrina < 20%) e revelou ser seguro, reduzir hospitalização por IC (217 x 294 hospitalizações; RR = 0,74; 95% CI 0,58-0,94, p = 0,013), embora sem impacto direto em redução de morte cardiovascular.^105^^,^^109^					

*IC: insuficiência cardíaca; ICFEr: insuficiência cardíaca com fração de ejeção reduzida.*

### 7.5. Algoritmo de Tratamento da Insuficiência Cardíaca com Fração de Ejeção Reduzida (Figura 7.1)

**Figura 7.1:**
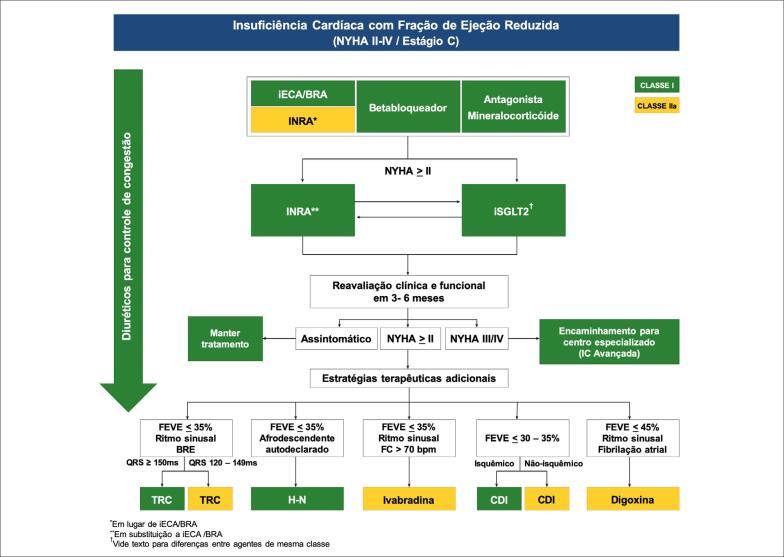
Algoritmo de tratamento da insuficiência cardíaca de fração de ejeção reduzida

*BRA: bloqueadores dos receptores da angiotensina II; BRE: bloqueio de ramo esquerdo; CDI: cardiodesfibrilador implantável; FEVE: fração de ejeção do ventrículo esquerdo; H-N: combinação de hidralazina e nitrato; IC: insuficiência cardíaca; iECA: inibidores da enzima conversora da angiotensina; INRA: inibidor da neprilisina e do receptor de angiotensina; iSGLT2: inibidores do cotransportador de sódio e glicose 2; NYHA: New York Heart Association; TRC: terapia de ressincronização cardíaca.*

## 8. Inovações em Outros Temas Relacionados à Insuficiência Cardíaca

### 8.1. Biomarcadores na Insuficiência Cardíaca com fração de Ejeção Reduzida (Tabela 8.1)

**Tabela 8.1:** Recomendações do uso de biomarcadores em pacientes com insuficiência cardíaca com fração de ejeção reduzida (ICFEr)

Recomendações	Classe	NE	Comentário	Tabela 2018	Ref.
Dosagem do BNP ou NT-proBNP quando há dúvida no diagnóstico da IC e como exame de triagem diagnóstica na atenção primária.	I	A	Recomendação de 2018 mantida.	Item 4.3 (página 451)	Vide 2018
Dosagem de BNP ou NT-proBNP para estratificação prognóstica em pacientes com IC.	I	A	Recomendação de 2018 mantida.	Item 4.3 (página 451)	Vide 2018
Medidas de BNP ou NT-proBNP como complemento ao exame físico para avaliar a resposta ao tratamento em pacientes com IC, em caso de dúvida quanto ao *status* clínico.	IIa	B	**MODIFICADO:** Dois estudos recentes, um randomizado e um observacional respaldam essa indicação.	Item 4.3 (página 451)	84, 110
Medidas seriadas de BNP ou NT-proBNP para guiar tratamento, com alvo do biomarcador a ser atingido.	IIb	B	**MODIFICADO:** Metanálise recente, que inclui dados do estudo *GUIDE-IT*, respaldam a indicação.	Item 4.3 (página 451)	111, 112
Peptídeos natriuréticos podem ser usados para avaliar a resposta a um determinado tratamento. No que diz respeito a esta estratégia, o tratamento é guiado clinicamente e o biomarcador é dosado antes e depois, sem um alvo específico. Novos estudos surgiram, confirmando o que já havia sido mostrado em subanálise do estudo *PARADIGM-HF*, no qual pacientes que reduziram NT-proBNP abaixo de 1.000 pg/mL, após a introdução de enalapril ou sacubitril-valsartana, apresentaram menos eventos de morte ou hospitalização por IC.^113^ No estudo *PIONEER-HF*, em pacientes hospitalizados por IC, acompanhados após a alta hospitalar, após 4 semanas se observou uma maior queda de NT-proBNP com sacubitril-valsartana do que com enalapril (46,7% *versus* 25,3%), e observou-se um menor número de eventos com sacubitril-valsartana.^84^ No estudo *PROVE-HF (Association of Change in N-Terminal Pro–B-Type Natriuretic Peptide Following Initiation of Sacubitril-Valsartan Treatment With Cardiac Structure and Function in Patients With Heart Failure With Reduced Ejection Fraction)*, em que pacientes com IC crônica utilizaram sacubitril-valsartana, houve queda significativa de NT-proBNP aos 14 dias de uso da medicação. A queda de NT-proBNP se associou a remodelagem reversa nos 12 meses de seguimento e mostrou menor taxa de eventos.^110^ A utilização de peptídeos para guiar o tratamento (com alvo de peptídeo natriurético a ser atingido), diferentemente da situação anterior, é controversa. Apesar dessa estratégia não ter sido superior em comparação ao manuseio convencional no estudo *GUIDE-IT (Effect of Natriuretic Peptide–Guided Therapy on Hospitalization or Cardiovascular Mortality in High-Risk Patients With Heart Failure and Reduced Ejection Fraction)*, existem outros levantamentos prévios que a demonstram.^112^ O estudo *PROTECT*^114^ mostrou redução nos eventos cardiovasculares ao se guiar por biomarcadores. Os estudos *TIME-CHF (BNP-guided vs symptom-guided heart failure therapy)*^115^ e Battlescarred^116^ mostraram redução de mortalidade com essa estratégia em pacientes abaixo de 75 anos de idade. Além disso, uma metanálise recente, feita com 4.554 pacientes e incorporando os pacientes do estudo *GUIDE-IT*, mostrou redução de hospitalizações e mortalidade por todas as causas com o tratamento guiado pelos peptídeos natriuréticos.^111^					

*IC: insuficiência cardíaca; NT: proBNP-fração N-terminal do peptídeo natriurético atrial do tipo B.*

### 8.2. Imunizações na Insuficiência Cardíaca (Tabela 8.2)

**Tabela 8.2:** Recomendações sobre imunização em pacientes com insuficiência cardíaca com fração de ejeção reduzida (ICFEr)

Recomendações	Classe	NE	Comentário	Tabela 2018	Ref.
Vacina contra influenza, para prevenção de fatores agravantes na IC e para redução de mortalidade na IC.	I	B	**MODIFICADA:** Novos estudos retrospectivos mostram benefício em mortalidade.	Item 6.7 (página 454)	117–120
Vacina contra pneumococos para prevenção de fatores agravantes na IC.	I	C	Recomendação de 2018 mantida.	Item 6.7 (página 454)	Vide 2018
Mais recentemente, não existiam dados sobre o impacto da *influenza* sobre os desfechos em pacientes com IC. No entanto, estudos populacionais recentes demonstraram a relação entre sazonalidade e maior número de hospitalizações por IC, evidenciado em quatro periodos consecutivos.^117^ Em uma subanálise do estudo *PARADIGM-HF*, 21% dos participantes receberam vacinação contra *influenza*, o que acarretou numa redução de morte total de 19% após o ajuste de propensão.^118^ Um estudo de corte dinamarquês feito com 134.048 pacientes com IC, que receberam > 1 vacinação entre 2005 e 2013, resultou em redução de 18% na mortalidade por todas as causas; e mais do que 3 vacinações associou-se a redução de 28% no risco de morte total e 29% de morte cardiovascular.^119^ Um estudo com banco de dados de 6.435 pacientes com IC, sendo 695 vacinados antes ou durante o inverno de 2017/2018, mostrou redução de 22% na morte total e de 17% na morte cardiovascular ou internação por IC. O benefício da vacinação sobre a morte total foi maior em pacientes com mais de 70 anos, com redução de mais de 25%.^120^ Não há estudos sobre o impacto da vacinação para pneumococos. Diversos estudos prospectivos estão em fase de inclusão de pacientes.					

*IC: insuficiência cardíaca.*

### 8.3. Indicação de Avaliação Genética nas Cardiomiopatias e na Insuficiência Cardíaca (Tabela 8.3)

**Tabela 8.3:** Recomendações sobre avaliação genética em pacientes com cardiomiopatias e IC

Recomendações	Classe	NE	Comentário	Tabela 2018	Ref.
Aconselhamento genético para pacientes e familiares com miocardiopatias hereditárias e mutação já identificada.	I	C	**NOVA:** Avanços nas técnicas de avaliação molecular genética permitem o reconhecimento precoce de miocardiopatias hereditárias, favorecendo a subclassificação de síndromes clínicas e seu tratamento individualizado.	Nova	121–125
Rastreamento de familiares em 1º grau de pacientes com miocardiopatias hereditárias.	I	C		Nova	121–125
Sequenciamento do gene da transtirretina em pacientes com diagnóstico de amiloidose cardíaca por deposição de transtirretina.	I	C		Nova	121–125
Avaliação molecular genética direcionada para avaliação etiológica e prognóstica de pacientes com fenótipo de miocardiopatia hereditária.	IIa	C		Nova	121–125
Avaliação molecular genética de rotina para pacientes com IC.	III	C		Nova	121–125
A incorporação do sequenciamento de última geração têm aumentado a sensibilidade dos testes genéticos, possibilitando o diagnóstico precoce com perspectiva de intervenção.^121^ Consequentemente, a avaliação molecular passa a fazer parte da rotina de avaliação de pacientes com miocardiopatias hereditárias, tais como: miocardiopatias hipertróficas, arritmogênica dilatada e/ou restritiva e miocárdio não compactado, em função do seu potencial de fornecer aconselhamento mais individualizado e preciso aos pacientes e familiares com estas doenças.^122^ Um exemplo claro desta necessidade é a diferenciação entre a miocardiopatia amiloidótica por transtirretina (ATTR) do “tipo selvagem” e a familiar: nas formas familiares, é imperativo o rastreamento em cascata de membros da família. É importante ressaltar que, atualmente, terapias utilizadas na ATTR apresentam particular benefício quando iniciadas em fase precoce da doença, conforme descrito no item 2, Tabela 2.4.^123^ Avanços na avaliação prognóstica, envolvendo genes com alto potencial arritmogênico também já foram descritos nas miocardiopatias dilatadas e arritmogênicas.^124^^,^^125^ Portanto, fica clara a necessidade de buscar formas mais eficientes da utilização da genética, especialmente no aconselhamento familiar, o que traz resultados seguros e sustentáveis na gestão do cuidado destes pacientes e de suas famílias.					

*IC: insuficiência cardíaca.*

## 9. Perspectivas na insuficiência cardíaca – Novas Moléculas

### 9.1. Estimuladores da Guanilato Ciclase (Tabela 9.1)

**Tabela 9.1:** Estimuladores da guanilato ciclase no tratamento de pacientes com insuficiência cardíaca com fração de ejeção reduzida (ICFEr)

Observações	Comentário	Tabela 2018	Ref.
Vericiguat em pacientes com FEVE menor que 45%, NYHA II – IV para reduzir morbidade, especialmente em pacientes com hospitalizações frequentes a despeito da terapia clínica otimizada.	**POTENCIAL:** A observação aqui descrita reflete dados de estudos recentes com esta nova classe de fármacos, porém ainda não aprovada pela Anvisa para uso no Brasil.	Nova	126, 127
Vericiguat age suprindo o déficit relativo de produção de GMP cíclico que ocorre em pacientes com IC^126^ e foi avaliado em um ensaio clínico randomizado, multicêntrico, controlado por placebo, duplo-cego em pacientes com ICFEr, o estudo *VICTORIA (Vericiguat in Patients with Heart Failure and Reduced Ejection Fraction).* Este estudo alocou 5.050 pacientes com ICFEr, com FEVE menor que 45%, em classe II-IV da NYHA, para receber Vericiguat 10 mg/dia, via oral, ou placebo, em adição a todo tratamento clínico. O desfecho primário foi morte cardiovascular ou primeira hospitalização por IC. Em um período de 11 meses, o desfecho primário ocorreu em 35,5% dos pacientes com vericiguat e em 38,5% dos pacientes tratados com placebo, sendo que 24 pacientes foram necessários para tratar (NNT) e salvar uma vida no período de 11 meses. O benefício do desfecho composto deveu-se prioritariamente à redução de hospitalizações, não havendo benefício estatisticamente significativo em morte cardiovascular ou mortalidade total.^127^ Esta medicação tem potencial de integrar o grupo de medicações com efeito sobre sintomas e re-hospitalizações em pacientes com ICFEr, sendo uma opção adicional em pacientes com hospitalizações frequentes a despeito de terapia otimizada e função renal ruim, já que o estudo considerava inclusão de pacientes com TFG maior que 15% ou com intolerância a outros fármacos. Devemos salientar que esta classe de fármacos é contraindicada em concomitância com nitratos.					

*FEVE: fração de ejeção do ventrículo esquerdo; IC: insuficiência cardíaca; ICFEr: insuficiência cardíaca com fração de ejeção reduzida; NYHA: New York Heart Association; TFG: taxa de filtração glomerular.*

### 9.2. Ativador Seletivo da Miosina Cardíaca (Tabela 9.2)

**Tabela 9.2:** Omecamtiv mecarbil no tratamento de pacientes com insuficiência cardíaca com fração de ejeção reduzida (ICFEr)

Observações	Comentário	Tabela 2018	Ref.
Omecamtiv mecarbil em pacientes com ICFEr aguda ou crônica.	**POTENCIAL:** A observação aqui descrita reflete dados de estudos recentes com esta nova classe de fármacos, porém ainda não aprovada pela Anvisa para uso no Brasil.	Nova	128–131
Omecamtiv mercabil é um ativador seletivo da miosina cardíaca e sua ação resulta na ativação e aumento da taxa de hidrólise de ATP, melhorando a contração ventricular em casos de ICFEr. Seu mecanismo de ação é diferente dos tratamentos atuais que bloqueiam a elevada estimulação neuro-hormonal. Os estudos mecanísticos, como o *ATOMIC-AHF* (*Acute Treatment with Omecamtiv Mecarbil to Increase Contractility in Acute Heart Failure*)^128^ e o *COSMIC-HF* (*Chronic Oral Study of Myosin Activation to Increase Contractility in Heart Failure*),^129^ mostraram que o fármaco melhora a contratilidade, melhorando a fração de ejeção, o volume ejetado e o débito cardíaco, além de outros parâmetros que indicam melhora da função cardíaca. Os estudos mostraram que promove redução dos níveis de NT-proBNP. Identificou-se também elevação dos níveis de troponina, sem alterações clínicas nos estudos realizados. O estudo *ATOMIC-AHF*, entretanto, em pacientes com IC aguda, não documentou redução da dispneia nos pacientes tratados. No ensaio clínico randomizado *GALACTIC-HF*, publicado recentemente, pacientes com ICFEr que receberam omecamtiv mecarbil apresentaram menor risco de desfecho composto por evento de IC (definido como hospitalização ou visitas não planejadas por piora da IC) ou morte cardiovascular do que aqueles que receberam placebo.^130^^,^^131^ No entanto, quando avaliados individualmente, não houve diferença nos desfechos secundários de morte por todas as causas, morte cardiovascular, primeira hospitalização por IC ou mudança no escore de qualidade de vida *Kansas City Cardiomyopathy Questionnaire*.					

*IC: insuficiência cardíaca; IC: insuficiência cardíaca; ICFEr: insuficiência cardíaca com fração de ejeção reduzida.*

## Appendix

**Table:**

Atualização de Tópicos Emergentes da Diretriz Brasileira de Insuficiência Cardíaca – 2021	
O relatório abaixo lista as declarações de interesse conforme relatadas à SBC pelos especialistas durante o período de desenvolvimento desta atualização, 2020.	
Especialista	Tipo de relacionamento com a indústria
Aguinaldo F. Freitas Jr.	Declaração financeira A - Pagamento de qualquer espécie e desde que economicamente apreciáveis, feitos a (i) você, (ii) ao seu cônjuge/companheiro ou a qualquer outro membro que resida com você, (iii) a qualquer pessoa jurídica em que qualquer destes seja controlador, sócio, acionista ou participante, de forma direta ou indireta, recebimento por palestras, aulas, atuação como proctor de treinamentos, remunerações, honorários pagos por participações em conselhos consultivos, de investigadores, ou outros comitês, etc. Provenientes da indústria farmacêutica, de órteses, próteses, equipamentos e implantes, brasileiras ou estrangeiras: - Novartis: Insuficiência cardíaca; Servier: Insuficiência cardíaca; Bayer: Anticoagulação.
Andréia Biolo	Nada a ser declarado
Antonio Carlos Pereira Barretto	Outros relacionamentos Financiamento de atividades de educação médica continuada, incluindo viagens, hospedagens e inscrições para congressos e cursos, provenientes da indústria farmacêutica, de órteses, próteses, equipamentos e implantes, brasileiras ou estrangeiras: - Novartis: sacubitril valsartana; Astrazeneca: dapagliflozina, succinato de metoprolol, candesartana; Servier: ivabradina.
Antônio José Lagoeiro Jorge	Outros relacionamentos Financiamento de atividades de educação médica continuada, incluindo viagens, hospedagens e inscrições para congressos e cursos, provenientes da indústria farmacêutica, de órteses, próteses, equipamentos e implantes, brasileiras ou estrangeiras: - Novartis: Entresto.
Bruno Biselli	Nada a ser declarado
Carlos Eduardo Lucena Montenegro	Declaração financeira A - Pagamento de qualquer espécie e desde que economicamente apreciáveis, feitos a (i) você, (ii) ao seu cônjuge/companheiro ou a qualquer outro membro que resida com você, (iii) a qualquer pessoa jurídica em que qualquer destes seja controlador, sócio, acionista ou participante, de forma direta ou indireta, recebimento por palestras, aulas, atuação como proctor de treinamentos, remunerações, honorários pagos por participações em conselhos consultivos, de investigadores, ou outros comitês, etc. Provenientes da indústria farmacêutica, de órteses, próteses, equipamentos e implantes, brasileiras ou estrangeiras: - Astrazeneca: palestras; Novartis: palestras; Servier: palestras; Merck: palestras. Outros relacionamentos Financiamento de atividades de educação médica continuada, incluindo viagens, hospedagens e inscrições para congressos e cursos, provenientes da indústria farmacêutica, de órteses, próteses, equipamentos e implantes, brasileiras ou estrangeiras: - Pfizer: Viagem para Congresso; AstraZeneca: Viagem para Simpósio; Novartis: Viagem para Simpósio.
Denilson Campos de Albuquerque	Declaração financeira B - Financiamento de pesquisas sob sua responsabilidade direta/pessoal (direcionado ao departamento ou instituição) provenientes da indústria farmacêutica, de órteses, próteses, equipamentos e implantes, brasileiras ou estrangeiras: - FAPERJ: Pesquisa Clínica; Boehringer: Participante de Pesquisa Clínica Multicêntrica Internacional.
Dirceu Rodrigues de Almeida	Nada a ser declarado
Edimar Alcides Bocchi	Declaração financeira A - Pagamento de qualquer espécie e desde que economicamente apreciáveis, feitos a (i) você, (ii) ao seu cônjuge/companheiro ou a qualquer outro membro que resida com você, (iii) a qualquer pessoa jurídica em que qualquer destes seja controlador, sócio, acionista ou participante, de forma direta ou indireta, recebimento por palestras, aulas, atuação como proctor de treinamentos, remunerações, honorários pagos por participações em conselhos consultivos, de investigadores, ou outros comitês, etc. Provenientes da indústria farmacêutica, de órteses, próteses, equipamentos e implantes, brasileiras ou estrangeiras: - AstraZeneca: ISGLT2; Bayer: ISGLT2, Vericiguat; Boehringer: ISGLT2.
Edval Gomes dos Santos Júnior	Nada a ser declarado
Estêvão Lanna Figueiredo	Declaração financeira B - Financiamento de pesquisas sob sua responsabilidade direta/pessoal (direcionado ao departamento ou instituição) provenientes da indústria farmacêutica, de órteses, próteses, equipamentos e implantes, brasileiras ou estrangeiras: - AstraZeneca: Dapagliflozina; Boehringer: Empagliflozina; Pfizer: Apixabana; Novartis.
Evandro Tinoco Mesquita	Outros relacionamentos Vínculo empregatício com a indústria farmacêutica, de órteses, próteses, Equipamentos e implantes, brasileiras ou estrangeiras, assim como se tem Relação vínculo empregatício com operadoras de planos de saúde ou em Auditorias médicas (incluindo meio período) durante o ano para o qual você está declarando: - UnitedHealth Group.
Fabiana G. Marcondes-Braga	Declaração financeira A - Pagamento de qualquer espécie e desde que economicamente apreciáveis, feitos a (i) você, (ii) ao seu cônjuge/companheiro ou a qualquer outro membro que resida com você, (iii) a qualquer pessoa jurídica em que qualquer destes seja controlador, sócio, acionista ou participante, de forma direta ou indireta, recebimento por palestras, aulas, atuação como proctor de treinamentos, remunerações, honorários pagos por participações em conselhos consultivos, de investigadores, ou outros comitês, etc. Provenientes da indústria farmacêutica, de órteses, próteses, equipamentos e implantes, brasileiras ou estrangeiras: - Novartis: Palestras; AstraZeneca: Palestras e Conselho Consultivo; Boehringer: Conselho Consultivo.
Fábio Fernandes	Declaração financeira A - Pagamento de qualquer espécie e desde que economicamente apreciáveis, feitos a (i) você, (ii) ao seu cônjuge/companheiro ou a qualquer outro membro que resida com você, (iii) a qualquer pessoa jurídica em que qualquer destes seja controlador, sócio, acionista ou participante, de forma direta ou indireta, recebimento por palestras, aulas, atuação como proctor de treinamentos, remunerações, honorários pagos por participações em conselhos consultivos, de investigadores, ou outros comitês, etc. Provenientes da indústria farmacêutica, de órteses, próteses, equipamentos e implantes, brasileiras ou estrangeiras: - Pfizer: Tafamidis; Alnylan: Patisiran.
Fabio Serra Silveira	Declaração financeira A - Pagamento de qualquer espécie e desde que economicamente apreciáveis, feitos a (i) você, (ii) ao seu cônjuge/companheiro ou a qualquer outro membro que resida com você, (iii) a qualquer pessoa jurídica em que qualquer destes seja controlador, sócio, acionista ou participante, de forma direta ou indireta, recebimento por palestras, aulas, atuação como proctor de treinamentos, remunerações, honorários pagos por participações em conselhos consultivos, de investigadores, ou outros comitês, etc. Provenientes da indústria farmacêutica, de órteses, próteses, equipamentos e implantes, brasileiras ou estrangeiras: - Novartis: Entresto; AstraZeneca: Forxiga; Servier: Procoralan. B - financiamento de pesquisas sob sua responsabilidade direta/pessoal (direcionado ao departamento ou instituição) provenientes da indústria farmacêutica, de órteses, próteses, equipamentos e implantes, brasileiras ou estrangeiras: - Amgen: Omecantiv mecarbil Outros relacionamentos Financiamento de atividades de educação médica continuada, incluindo viagens, hospedagens e inscrições para congressos e cursos, provenientes da indústria farmacêutica, de órteses, próteses, equipamentos e implantes, brasileiras ou estrangeiras: - Ache: Hipertensão
Felix José Alvarez Ramires	Declaração financeira A - Pagamento de qualquer espécie e desde que economicamente apreciáveis, feitos a (i) você, (ii) ao seu cônjuge/companheiro ou a qualquer outro membro que resida com você, (iii) a qualquer pessoa jurídica em que qualquer destes seja controlador, sócio, acionista ou participante, de forma direta ou indireta, recebimento por palestras, aulas, atuação como proctor de treinamentos, remunerações, honorários pagos por participações em conselhos consultivos, de investigadores, ou outros comitês, etc. Provenientes da indústria farmacêutica, de órteses, próteses, equipamentos e implantes, brasileiras ou estrangeiras: - Novartis: Sacubitril/Valsartana; Pfizer: Patisiran; Merck: Vericiquat; Amgen.
Fernando Antibas Atik	Nada a ser declarado
Fernando Bacal	Nada a ser declarado
Flávio de Souza Brito	Declaração financeira A - Pagamento de qualquer espécie e desde que economicamente apreciáveis, feitos a (i) você, (ii) ao seu cônjuge/companheiro ou a qualquer outro membro que resida com você, (iii) a qualquer pessoa jurídica em que qualquer destes seja controlador, sócio, acionista ou participante, de forma direta ou indireta, recebimento por palestras, aulas, atuação como proctor de treinamentos, remunerações, honorários pagos por participações em conselhos consultivos, de investigadores, ou outros comitês, etc. Provenientes da indústria farmacêutica, de órteses, próteses, equipamentos e implantes, brasileiras ou estrangeiras: - Novartis: Entresto; Servier: Procoralan; Merck: Concor.
Germano Emílio Conceição Souza	Declaração financeira A - Pagamento de qualquer espécie e desde que economicamente apreciáveis, feitos a (i) você, (ii) ao seu cônjuge/companheiro ou a qualquer outro membro que resida com você, (iii) a qualquer pessoa jurídica em que qualquer destes seja controlador, sócio, acionista ou participante, de forma direta ou indireta, recebimento por palestras, aulas, atuação como proctor de treinamentos, remunerações, honorários pagos por participações em conselhos consultivos, de investigadores, ou outros comitês, etc. Provenientes da indústria farmacêutica, de órteses, próteses, equipamentos e implantes, brasileiras ou estrangeiras: - Novartis: Insuficiência Cardíaca; Merck: Insuficiência Cardíaca. B - Financiamento de pesquisas sob sua responsabilidade direta/pessoal (direcionado ao departamento ou instituição) provenientes da indústria farmacêutica, de órteses, próteses, equipamentos e implantes, brasileiras ou estrangeiras: - Bayer: Rivaroxabana; Outros relacionamentos Participação societária de qualquer natureza e qualquer valor economicamente apreciável de empresas na área de saúde, de ensino ou em empresas concorrentes ou fornecedoras da SBC: - Área da saúde: Prestação de serviços PJ médica, área assistencial - Ensino: Empresa de ensino técnico para aulas e cursos na área de saúde
Gustavo Calado de Aguiar Ribeiro	Nada a ser declarado
Humberto Villacorta	Declaração financeira A - Pagamento de qualquer espécie e desde que economicamente apreciáveis, feitos a (i) você, (ii) ao seu cônjuge/companheiro ou a qualquer outro membro que resida com você, (iii) a qualquer pessoa jurídica em que qualquer destes seja controlador, sócio, acionista ou participante, de forma direta ou indireta, recebimento por palestras, aulas, atuação como proctor de treinamentos, remunerações, honorários pagos por participações em conselhos consultivos, de investigadores, ou outros comitês, etc. Provenientes da indústria farmacêutica, de órteses, próteses, equipamentos e implantes, brasileiras ou estrangeiras: - Novartis: Insuficiência Cardíaca; Roche: Biomarcadores; Servier: Insuficiência Cardíaca. C - Financiamento de pesquisa (pessoal), cujas receitas tenham sido provenientes da indústria farmacêutica, de órteses, próteses, equipamentos e implantes, brasileiras ou estrangeiras: - Roche: GDF-15.
Jefferson Luis Vieira	Nada a ser declarado
João David de Souza Neto	Nada a ser declarado
João Manoel Rossi Neto	Declaração financeira A - Pagamento de qualquer espécie e desde que economicamente apreciáveis, feitos a (i) você, (ii) ao seu cônjuge/companheiro ou a qualquer outro membro que resida com você, (iii) a qualquer pessoa jurídica em que qualquer destes seja controlador, sócio, acionista ou participante, de forma direta ou indireta, recebimento por palestras, aulas, atuação como proctor de treinamentos, remunerações, honorários pagos por participações em conselhos consultivos, de investigadores, ou outros comitês, etc. Provenientes da indústria farmacêutica, de órteses, próteses, equipamentos e implantes, brasileiras ou estrangeiras: - Novartis: Aulas; AstraZeneca: Aulas.
José Albuquerque de Figueiredo Neto	Declaração financeira A - Pagamento de qualquer espécie e desde que economicamente apreciáveis, feitos a (i) você, (ii) ao seu cônjuge/companheiro ou a qualquer outro membro que resida com você, (iii) a qualquer pessoa jurídica em que qualquer destes seja controlador, sócio, acionista ou participante, de forma direta ou indireta, recebimento por palestras, aulas, atuação como proctor de treinamentos, remunerações, honorários pagos por participações em conselhos consultivos, de investigadores, ou outros comitês, etc. Provenientes da indústria farmacêutica, de órteses, próteses, equipamentos e implantes, brasileiras ou estrangeiras: - Novartis: Insuficiência Cardíaca.
Lídia Ana Zytynski Moura	Declaração financeira A - Pagamento de qualquer espécie e desde que economicamente apreciáveis, feitos a (i) você, (ii) ao seu cônjuge/companheiro ou a qualquer outro membro que resida com você, (iii) a qualquer pessoa jurídica em que qualquer destes seja controlador, sócio, acionista ou participante, de forma direta ou indireta, recebimento por palestras, aulas, atuação como proctor de treinamentos, remunerações, honorários pagos por participações em conselhos consultivos, de investigadores, ou outros comitês, etc. Provenientes da indústria farmacêutica, de órteses, próteses, equipamentos e implantes, brasileiras ou estrangeiras: - Novartis: Entresto; AstraZeneca: Forxiga. B - Financiamento de pesquisas sob sua responsabilidade direta/pessoal (direcionado ao departamento ou instituição) provenientes da indústria farmacêutica, de órteses, próteses, equipamentos e implantes, brasileiras ou estrangeiras: - AstraZeneca: Forxiga.
Livia Adams Goldraich	Nada a ser declarado
Luís Beck-da-Silva	Declaração financeira A - Pagamento de qualquer espécie e desde que economicamente apreciáveis, feitos a (i) você, (ii) ao seu cônjuge/companheiro ou a qualquer outro membro que resida com você, (iii) a qualquer pessoa jurídica em que qualquer destes seja controlador, sócio, acionista ou participante, de forma direta ou indireta, recebimento por palestras, aulas, atuação como proctor de treinamentos, remunerações, honorários pagos por participações em conselhos consultivos, de investigadores, ou outros comitês, etc. Provenientes da indústria farmacêutica, de órteses, próteses, equipamentos e implantes, brasileiras ou estrangeiras: - Novartis: Insuficiência Cardíaca; AstraZeneca: Insuficiência Cardíaca. B - Financiamento de pesquisas sob sua responsabilidade direta/pessoal (direcionado ao departamento ou instituição) provenientes da indústria farmacêutica, de órteses, próteses, equipamentos e implantes, brasileiras ou estrangeiras: - Amgen: Insuficiência Cardíaca
Luis Eduardo Rohde	Declaração financeira A - Pagamento de qualquer espécie e desde que economicamente apreciáveis, feitos a (i) você, (ii) ao seu cônjuge/companheiro ou a qualquer outro membro que resida com você, (iii) a qualquer pessoa jurídica em que qualquer destes seja controlador, sócio, acionista ou participante, de forma direta ou indireta, recebimento por palestras, aulas, atuação como proctor de treinamentos, remunerações, honorários pagos por participações em conselhos consultivos, de investigadores, ou outros comitês, etc. Provenientes da indústria farmacêutica, de órteses, próteses, equipamentos e implantes, brasileiras ou estrangeiras: - AstraZeneca: Dapaglifozina; Novartis: Sacubitril-Valsartana; Amgen: Omecamtiv Mecarbil; Merck; Bayer.
Luiz Claudio Danzmann	Declaração financeira A - Pagamento de qualquer espécie e desde que economicamente apreciáveis, feitos a (i) você, (ii) ao seu cônjuge/companheiro ou a qualquer outro membro que resida com você, (iii) a qualquer pessoa jurídica em que qualquer destes seja controlador, sócio, acionista ou participante, de forma direta ou indireta, recebimento por palestras, aulas, atuação como proctor de treinamentos, remunerações, honorários pagos por participações em conselhos consultivos, de investigadores, ou outros comitês, etc. Provenientes da indústria farmacêutica, de órteses, próteses, equipamentos e implantes, brasileiras ou estrangeiras: - Novartis: Entresto; AstraZeneca: Forxiga; Servier: Procoralan.
Manoel Fernandes Canesin	Nada a ser declarado
Marcelo Imbroinise Bittencourt	Declaração financeira A - Pagamento de qualquer espécie e desde que economicamente apreciáveis, feitos a (i) você, (ii) ao seu cônjuge/companheiro ou a qualquer outro membro que resida com você, (iii) a qualquer pessoa jurídica em que qualquer destes seja controlador, sócio, acionista ou participante, de forma direta ou indireta, recebimento por palestras, aulas, atuação como proctor de treinamentos, remunerações, honorários pagos por participações em conselhos consultivos, de investigadores, ou outros comitês, etc. Provenientes da indústria farmacêutica, de órteses, próteses, equipamentos e implantes, brasileiras ou estrangeiras: - Geneone - dasa: testes genéticos; Sanofi: Terapia de reposição enzimática; AstraZeneca: Forxiga.
Marcelo Westerlund Montera	Nada a ser declarado
Marcely Gimenes Bonatto	Declaração financeira A - Pagamento de qualquer espécie e desde que economicamente apreciáveis, feitos a (i) você, (ii) ao seu cônjuge/companheiro ou a qualquer outro membro que resida com você, (iii) a qualquer pessoa jurídica em que qualquer destes seja controlador, sócio, acionista ou participante, de forma direta ou indireta, recebimento por palestras, aulas, atuação como proctor de treinamentos, remunerações, honorários pagos por participações em conselhos consultivos, de investigadores, ou outros comitês, etc. Provenientes da indústria farmacêutica, de órteses, próteses, equipamentos e implantes, brasileiras ou estrangeiras: - Novartis: Sacubitril/Valsartana; AstraZeneca: Forxiga.
Marcus Vinícius Simões	Declaração financeira A - Pagamento de qualquer espécie e desde que economicamente apreciáveis, feitos a (i) você, (ii) ao seu cônjuge/companheiro ou a qualquer outro membro que resida com você, (iii) a qualquer pessoa jurídica em que qualquer destes seja controlador, sócio, acionista ou participante, de forma direta ou indireta, recebimento por palestras, aulas, atuação como proctor de treinamentos, remunerações, honorários pagos por participações em conselhos consultivos, de investigadores, ou outros comitês, etc. Provenientes da indústria farmacêutica, de órteses, próteses, equipamentos e implantes, brasileiras ou estrangeiras: - Novartis: entresto; AstraZeneca: Dapagliflozina. B - Financiamento de pesquisas sob sua responsabilidade direta/pessoal (direcionado ao departamento ou instituição) provenientes da indústria farmacêutica, de órteses, próteses, equipamentos e implantes, brasileiras ou estrangeiras: - Amgen: Omecamtiv/Mecarbil; Beringher Ingelheim: Empagliflozina.
Maria da Consolação Vieira Moreira	Nada a ser declarado
Miguel Morita Fernandes-Silva	Declaração financeira A - Pagamento de qualquer espécie e desde que economicamente apreciáveis, feitos a (i) você, (ii) ao seu cônjuge/companheiro ou a qualquer outro membro que resida com você, (iii) a qualquer pessoa jurídica em que qualquer destes seja controlador, sócio, acionista ou participante, de forma direta ou indireta, recebimento por palestras, aulas, atuação como proctor de treinamentos, remunerações, honorários pagos por participações em conselhos consultivos, de investigadores, ou outros comitês, etc. Provenientes da indústria farmacêutica, de órteses, próteses, equipamentos e implantes, brasileiras ou estrangeiras: - Novartis: Insuficiência Cardíaca C - Financiamento de pesquisa (pessoal), cujas receitas tenham sido provenientes da indústria farmacêutica, de órteses, próteses, equipamentos e implantes, brasileiras ou estrangeiras: - Amgen: Omecamtiv/Insuficiência Cardíaca; Beringher Ingelheim: Empagliflozina.
Mônica Samuel Avila	Nada a ser declarado
Mucio Tavares de Oliveira Junior	Declaração financeira B - Financiamento de pesquisas sob sua responsabilidade direta/pessoal (direcionado ao departamento ou instituição) provenientes da indústria farmacêutica, de órteses, próteses, equipamentos e implantes, brasileiras ou estrangeiras: - Torrent do Brasil: Droga em desenvolvimento; Sanofi Pasteur: Vacina para gripe.
Nadine Clausell	Nada a ser declarado
Odilson Marcos Silvestre	Nada a ser declarado
Otavio Rizzi Coelho Filho	Declaração financeira A - Pagamento de qualquer espécie e desde que economicamente apreciáveis, feitos a (i) você, (ii) ao seu cônjuge/companheiro ou a qualquer outro membro que resida com você, (iii) a qualquer pessoa jurídica em que qualquer destes seja controlador, sócio, acionista ou participante, de forma direta ou indireta, recebimento por palestras, aulas, atuação como proctor de treinamentos, remunerações, honorários pagos por participações em conselhos consultivos, de investigadores, ou outros comitês, etc. Provenientes da indústria farmacêutica, de órteses, próteses, equipamentos e implantes, brasileiras ou estrangeiras: - Pfizer: Amiloidose cardíaca; Alnylam: Amiloidose cardíaca; AstraZeneca: Insuficiência Cardíaca; Novartis. B - Financiamento de pesquisas sob sua responsabilidade direta/pessoal (direcionado ao departamento ou instituição) provenientes da indústria farmacêutica, de órteses, próteses, equipamentos e implantes, brasileiras ou estrangeiras: - Pfizer: Amiloidose cardíaca. Outros relacionamentos Financiamento de atividades de educação médica continuada, incluindo viagens, hospedagens e inscrições para congressos e cursos, provenientes da indústria farmacêutica, de órteses, próteses, equipamentos e implantes, brasileiras ou estrangeiras: - AstraZeneca: Insuficiência Cardíaca; Pfizer: Insuficiência Cardíaca.
Pedro Vellosa Schwartzmann	Declaração financeira A - Pagamento de qualquer espécie e desde que economicamente apreciáveis, feitos a (i) você, (ii) ao seu cônjuge/companheiro ou a qualquer outro membro que resida com você, (iii) a qualquer pessoa jurídica em que qualquer destes seja controlador, sócio, acionista ou participante, de forma direta ou indireta, recebimento por palestras, aulas, atuação como proctor de treinamentos, remunerações, honorários pagos por participações em conselhos consultivos, de investigadores, ou outros comitês, etc. Provenientes da indústria farmacêutica, de órteses, próteses, equipamentos e implantes, brasileiras ou estrangeiras: - Novartis: Entresto; Servier: Ivabradina; AstraZeneca: Dapagliflozina; Merck: Serono. B - Financiamento de pesquisas sob sua responsabilidade direta/pessoal (direcionado ao departamento ou instituição) provenientes da indústria farmacêutica, de órteses, próteses, equipamentos e implantes, brasileiras ou estrangeiras: - Novartis: investigacional; Eidos: AG10. Outros relacionamentos Financiamento de atividades de educação médica continuada, incluindo viagens, hospedagens e inscrições para congressos e cursos, provenientes da indústria farmacêutica, de órteses, próteses, equipamentos e implantes, brasileiras ou estrangeiras: - Bayer: Rivaroxaban; AstraZeneca: Dapagliflozina.
Reinaldo Bulgarelli Bestetti	Nada a ser declarado
Ricardo Mourilhe-Rocha	Declaração financeira A - Pagamento de qualquer espécie e desde que economicamente apreciáveis, feitos a (i) você, (ii) ao seu cônjuge/companheiro ou a qualquer outro membro que resida com você, (iii) a qualquer pessoa jurídica em que qualquer destes seja controlador, sócio, acionista ou participante, de forma direta ou indireta, recebimento por palestras, aulas, atuação como proctor de treinamentos, remunerações, honorários pagos por participações em conselhos consultivos, de investigadores, ou outros comitês, etc. Provenientes da indústria farmacêutica, de órteses, próteses, equipamentos e implantes, brasileiras ou estrangeiras: - AstraZeneca: Dapagliflozina; Boehringer: Empagliflozina; Novartis: Sacubitril/Valsartana. B - Financiamento de pesquisas sob sua responsabilidade direta/pessoal (direcionado ao departamento ou instituição) provenientes da indústria farmacêutica, de órteses, próteses, equipamentos e implantes, brasileiras ou estrangeiras: - PROADI/SUS: Telemedicina; Boehringer: Empagliflozina.
Sabrina Bernadez-Pereira	Nada a ser declarado
Salvador Rassi	Declaração financeira A - Pagamento de qualquer espécie e desde que economicamente apreciáveis, feitos a (i) você, (ii) ao seu cônjuge/companheiro ou a qualquer outro membro que resida com você, (iii) a qualquer pessoa jurídica em que qualquer destes seja controlador, sócio, acionista ou participante, de forma direta ou indireta, recebimento por palestras, aulas, atuação como proctor de treinamentos, remunerações, honorários pagos por participações em conselhos consultivos, de investigadores, ou outros comitês, etc. Provenientes da indústria farmacêutica, de órteses, próteses, equipamentos e implantes, brasileiras ou estrangeiras: - Novartis: Entresto; Servier: Procoralan. B - Financiamento de pesquisas sob sua responsabilidade direta/pessoal (direcionado ao departamento ou instituição) provenientes da indústria farmacêutica, de órteses, próteses, equipamentos e implantes, brasileiras ou estrangeiras: - Novartis: Entresto; Servier: Procoralan; Boehringer Ingelheim: Jardiance. Outros relacionamentos Financiamento de atividades de educação médica continuada, incluindo viagens, hospedagens e inscrições para congressos e cursos, provenientes da indústria farmacêutica, de órteses, próteses, equipamentos e implantes, brasileiras ou estrangeiras: - Novartis: Entresto; Servier: Procoralan.
Sandrigo Mangini	Declaração financeira A - Pagamento de qualquer espécie e desde que economicamente apreciáveis, feitos a (i) você, (ii) ao seu cônjuge/companheiro ou a qualquer outro membro que resida com você, (iii) a qualquer pessoa jurídica em que qualquer destes seja controlador, sócio, acionista ou participante, de forma direta ou indireta, recebimento por palestras, aulas, atuação como proctor de treinamentos, remunerações, honorários pagos por participações em conselhos consultivos, de investigadores, ou outros comitês, etc. Provenientes da indústria farmacêutica, de órteses, próteses, equipamentos e implantes, brasileiras ou estrangeiras: - Novartis: Sacubitril/Valsartan; Pfizer: Doenças raras Outros relacionamentos Financiamento de atividades de educação médica continuada, incluindo viagens, hospedagens e inscrições para congressos e cursos, provenientes da indústria farmacêutica, de órteses, próteses, equipamentos e implantes, brasileiras ou estrangeiras: - Pfizer: Doenças raras
Silvia Marinho Martins Alves	Nada a ser declarado
Silvia Moreira Ayub Ferreira	Declaração financeira A - Pagamento de qualquer espécie e desde que economicamente apreciáveis, feitos a (i) você, (ii) ao seu cônjuge/companheiro ou a qualquer outro membro que resida com você, (iii) a qualquer pessoa jurídica em que qualquer destes seja controlador, sócio, acionista ou participante, de forma direta ou indireta, recebimento por palestras, aulas, atuação como proctor de treinamentos, remunerações, honorários pagos por participações em conselhos consultivos, de investigadores, ou outros comitês, etc. Provenientes da indústria farmacêutica, de órteses, próteses, equipamentos e implantes, brasileiras ou estrangeiras: - Abbott: Mitraclip; Novartis: Entresto. Outros relacionamentos Financiamento de atividades de educação médica continuada, incluindo viagens, hospedagens e inscrições para congressos e cursos, provenientes da indústria farmacêutica, de órteses, próteses, equipamentos e implantes, brasileiras ou estrangeiras: - Abbott: Heartmate II e HeartMate 3.
Victor Sarli Issa	Nada a ser declarado
