# Supplementary material for: Atualização de Tópicos Emergentes da Diretriz Brasileira de Insuficiência Cardíaca – 2021
Source: Arq Bras Cardiol. 2021 Jun 8;116(6):1174–212. [Article in Portuguese] doi: 10.36660/abc.20210367 (PMC8288520; doi:10.36660/abc.20210367)
Supplement: Translation

# Translation: Emerging Topics Update of the Brazilian Heart Failure Guideline – 2021

**Authors:** Fabiana G. Marcondes-Braga, Lídia Ana Zytynski Moura, Victor Sarli Issa, Jefferson Luis Vieira, Luis Eduardo Rohde, Marcus Vinícius Simões, Miguel Morita Fernandes-Silva, Salvador Rassi, Silvia Marinho Martins Alves, Denilson Campos de Albuquerque, Dirceu Rodrigues de Almeida, Edimar Alcides Bocchi, Felix José Alvarez Ramires, Fernando Bacal, João Manoel Rossi, Luiz Claudio Danzmann, Marcelo Westerlund Montera, Mucio Tavares de Oliveira, Nadine Clausell, Odilson Marcos Silvestre, Reinaldo Bulgarelli Bestetti, Sabrina Bernadez-Pereira, Aguinaldo F. Freitas, Andréia Biolo, Antonio Carlos Pereira Barretto, Antônio José Lagoeiro Jorge, Bruno Biselli, Carlos Eduardo Lucena Montenegro, Edval Gomes dos Santos, Estêvão Lanna Figueiredo, Fábio Fernandes, Fabio Serra Silveira, Fernando Antibas Atik, Flávio de Souza Brito, Germano Emílio Conceição Souza, Gustavo Calado de Aguiar Ribeiro, Humberto Villacorta, João David de Souza, Livia Adams Goldraich, Luís Beck-da-Silva, Manoel Fernandes Canesin, Marcelo Imbroinise Bittencourt, Marcely Gimenes Bonatto, Maria da Consolação Vieira Moreira, Mônica Samuel Avila, Otavio Rizzi Coelho, Pedro Vellosa Schwartzmann, Ricardo Mourilhe-Rocha, Sandrigo Mangini, Silvia Moreira Ayub Ferreira, José Albuquerque de Figueiredo, Evandro Tinoco Mesquita

**Affiliations:** 1 São PauloSP Brazil Instituto do Coração (InCor) do Hospital das Clínicas da Faculdade de Medicina da Universidade de São Paulo (HCFMUSP), São Paulo, SP – Brazil.; 2 CuritibaPR Brazil Pontifícia Universidade Católica de Curitiba, Curitiba, PR – Brazil.; 3 Belgium Universidade da Antuérpia, – Belgium; 4 FortalezaCE Brazil Hospital do Coração de Messejana Dr. Carlos Alberto Studart Gomes, Fortaleza, CE – Brazil.; 5 Porto AlegeRS Brazil Hospital de Clínicas de Porto Alegre, Porto Alege, RS – Brazil.; 6 Porto AlegreRS Brazil Hospital Moinhos de Vento, Porto Alegre, RS – Brazil.; 7 Porto AlegreRS Brazil Universidade Federal do Rio Grande do Sul (UFRGS), Porto Alegre, RS – Brazil.; 8 São PauloSP Brazil Faculdade de Medicina de Ribeirão Preto da Universidade de São Paulo, São Paulo, SP – Brazil.; 9 CuritibaPR Brazil Universidade Federal do Paraná (UFPR), Curitiba, PR – Brazil.; 10 CuritibaPR Brazil Quanta Diagnóstico por Imagem, Curitiba, PR – Brazil.; 11 GoiâniaGO Brazil Hospital das Clínicas da Universidade Federal de Goiás (UFGO), Goiânia, GO – Brazil.; 12 RecifePE Brazil Pronto Socorro Cardiológico de Pernambuco (PROCAPE), Recife, PE – Brazil.; 13 RecifePE Brazil Universidade de Pernambuco (UPE), Recife, PE – Brazil.; 14 Rio de JaneiroRJ Brazil Universidade do Estado do Rio de Janeiro (UERJ), Rio de Janeiro, RJ – Brazil.; 15 São PauloSP Brazil Universidade Federal de São Paulo (UNIFESP), São Paulo, SP – Brazil.; 16 São PauloSP Brazil Hospital de Coração (HCor), São Paulo, SP – Brazil.; 17 São PauloSP Brazil Hospital Israelita Albert Einstein, São Paulo, SP – Brazil.; 18 São PauloSP Brazil Instituto Dante Pazzanese de Cardiologia, São Paulo, SP – Brazil.; 19 CanoasRS Brazil Universidade Luterana do Brazil, Canoas, RS – Brazil.; 20 Porto AlegreRS Brazil Hospital São Lucas da PUC-RS, Porto Alegre, RS – Brazil.; 21 Rio de JaneiroRJ Brazil Hospital Pró-Cardíaco, Rio de Janeiro, RJ – Brazil.; 22 Rio BrancoAC Brazil Universidade Federal do Acre, Rio Branco, AC – Brazil.; 23 Ribeirão PretoSP Brazil Departamento de Medicina da Universidade de Ribeirão Preto (UNAERP), Ribeirão Preto, SP – Brazil.; 24 NiteróiRJ Brazil Faculdade de Medicina da Universidade Federal Fluminense (UFF), Niterói, RJ – Brazil.; 25 Feira de SantanaBA Brazil Universidade Estadual de Feira de Santana, Feira de Santana, BA – Brazil.; 26 Feira de SantanaBA Brazil Santa Casa de Misericórdia de Feira de Santana, Feira de Santana, BA – Brazil.; 27 Belo HorizonteMG Brazil Instituto Orizonti, Belo Horizonte, MG – Brazil.; 28 Belo HorizonteMG Brazil Hospital Vera Cruz, Belo Horizonte, MG – Brazil.; 29 AracajuSE Brazil Fundação Beneficência Hospital de Cirurgia (FBHC-Ebserh), Aracaju, SE – Brazil.; 30 AracajuSE Brazil Centro de Pesquisa Clínica do Coração, Aracaju, SE – Brazil.; 31 BrasíliaDF Brazil Universidade de Brasília (UnB), Brasília, DF – Brazil.; 32 São PauloSP Brazil Universidade Estadual Paulista Júlio de Mesquita Filho (UNESP), São Paulo, SP – Brazil.; 33 São PauloSP Brazil Hospital Alemão Oswaldo Cruz, São Paulo, SP – Brazil.; 34 São PauloSP Brazil Hospital Regional de São José dos Campos, São Paulo, SP – Brazil.; 35 CampinasSP Brazil Pontifícia Universidade Católica de Campinas (PUCC), Campinas, SP – Brazil.; 36 LondrinaPR Brazil Hospital Universitário da Universidade Estadual de Londrina, Londrina, PR – Brazil.; 37 Rio de JaneiroRJ Brazil Hospital Universitário Pedro Ernesto, Rio de Janeiro, RJ – Brazil.; 38 CuritibaPR Brazil Hospital Santa Casa de Misericórdia de Curitiba, Curitiba, PR – Brazil.; 39 Belo HorizonteMG Brazil Universidade Federal de Minas Gerais (UFMG), Belo Horizonte, MG – Brazil.; 40 CampinasSP Brazil Faculdade de Ciências Médicas da Universidade Estadual de Campinas (UNICAMP), Campinas, SP – Brazil.; 41 Ribeirão PretoSP Brazil Hospital Unimed Ribeirão Preto, Ribeirão Preto, SP – Brazil.; 42 Ribeirão PretoSP Brazil Centro Avançado de Pesquisa, Ensino e Diagnóstico (CAPED), Ribeirão Preto, SP – Brazil.; 43 São LuísMA Brazil Universidade Federal do Maranhão (UFMA), São Luís, MA – Brazil.; 44 Rio de JaneiroRJ Brazil Centro de Ensino e Treinamento Edson de Godoy Bueno / UHG, Rio de Janeiro, RJ – Brazil.

## Introduction

The latest Heart Failure Guidelines by the Department of Heart Failure of the Brazilian Society of Cardiology (DEIC/SBC) were finalized on March 2018. Since then, a significant number of therapeutic interventions and diagnostic approaches has arisen or consolidated their position in international clinical practice and in clinical research. In addition, the COVID-19 pandemic has taught us much about the pathophysiological model of myocardial damage and raised many questions about the continuity and safety of medication use in patients with chronic HF suffering from an acute manifestation of this new and complex clinical entity.

In the last few months, we have been working quickly and collaboratively, and for the first time in 20 years, DEIC used digital platforms to discuss, deliberate, and draft this important document, opting for a focused update instead of a full-text guideline.

We were inspired by the 2020 Canadian Heart Failure Guidelines,^1^ but had the benefit of observing the impact on clinical practice and the consolidation of this new knowledge, in addition to new results from clinical trials published over the last 12 months. In order to report on these developments, we hosted a pioneering scientific conference on September 19, 2020, the I Heart Failure Summit Brazil 2020 (digital), with approximately 900 participants, many of them DEIC associates.

The leadership of the Science Board was key in organizing the various working groups and developing a secure and practical method for discussions and votes. With social distancing and the use of digital technology, the conference enabled wide-ranging debates from various perspectives, based on the best available scientific evidence.

In this document, DEIC/SBC provides reviews and detailed updates to its Chronic Heart Failure Guidelines. The work started in July 2020, with the choice of the Editorial Board, which established priorities, divided the 52 participants into working groups, and developed a schedule of activities. These working groups, each consisting of five to seven participants, began intense online discussions that led to the elaboration of preliminary tables, widely circulated before their subsequent review by the 11-member Review Board. The final discussions took place during a virtual plenary session on December 4, 2020, with all collaborators, who had the opportunity to vote on the main recommendations. Decisions regarding classes of recommendation required a three-quarters supermajority vote.

Class of Recommendation and Level of Evidence follow the same definitions used in the last guideline, as established by SBC/CONDir. See below.

The therapeutic recommendations proposed in this document are based on the latest available scientific evidence, considering not only the aspects of clinical efficacy from large clinical trials. We have sought to summarize the primary recommendations in flowcharts and algorithms that are easy to understand and to apply in clinical practice, proposing approaches for the diagnosis and treatment of heart failure.

Our commitment to the scientific community, linked to research and assistance to heart failure patients, public and private managers, and policy-makers, will certainly have the benefit of a document that sought to present scientific interventions in an accessible format, facilitating its implementation in the various spheres where heart failure patients receive care.

**Table:** 

**Classes of Recommendation**
**Class I**
Conditions for which there is conclusive evidence or, if not, there is general agreement that the procedure is safe and useful/effective.
**Class II**
Conditions for which there is conflicting evidence and/or divergence of opinion about the safety and usefulness/efficacy of the procedure.
**Class IIA**
Weight or evidence/opinion in favor of the intervention. Most approve.
**Class IIB**
Less well-established safety and usefulness/efficacy, without predominance.
**Class III**
Conditions for which there is evidence and/or general agreement that the procedure is not useful/effective, and in some cases may be harmful.
**Levels of Evidence**
**Level A**
Data derived from multiple consistent randomized controlled clinical trials, and/or a robust meta-analysis of randomized clinical trials.
**Level B**
Data derived from a less robust meta-analysis, one single randomized trial or non-randomized trials (observational).
**Level C**
Data derived from consensual expert opinion.

**Dr. Evandro Tinoco Mesquita**

## 1. Innovations in Heart Failure with Preserved (HFpEF), Mildly Reduced (HFmrEF) and Improved (HFimpEF) Ejection Fraction

### 1.1. Diagnosis of Heart Failure with Preserved Ejection Fraction (HFpEF)

In patients with unexplained fatigue or dyspnea, assessing the pretest probability of heart failure (HF) should be based on clinical, electrocardiography, echocardiography, and laboratory data. Next, two scoring systems have been developed to check that diagnosis; both the H_2_FPEF (Table 1.1) and the HFA-PEFF (Table 1.2) scores may be used. In these models, high- and low-probability patients can be classified as having or not heart failure with preserved ejection fraction (HFpEF), respectively. In patients with intermediary probability for HFpEF, assessing diastolic function during stress, which can be based on a diastolic stress echocardiogram or invasive hemodynamic monitoring, can help the diagnosis. In patients with low probability of HFpEF, investigating other causes of dyspnea and fatigue is recommended^2^ (Figure 1.1 and Table 1.3).

**Figure 1.1:** Diagnostic flowchart for heart failure with preserved ejection fraction (HFpEF)

*Adapted from Borlaug BA.2 Nat Rev Cardiol. 2020; 17:559-573. ECG: electrocardiogram; HF: heart failure; LVEF: left ventricular ejection fraction.*

**Tabela 1.1:** H_2_FPEF score for HFpEF diagnosis

	Clinical Variable	Characteristics	Points
H_2_	***H****eavy*	BMI > 30 Kg/m^2^	2
	**H**ypertension	2 or more anti-hypertensive drugs	1
F	Atrial **F**ibrillation	Paroxysmal or Persistent	3
P	**P**ulmonary Hypertension	PASP > 35 mmHg	1
E	**E**lderly	Age > 60 years	1
F	**F**illing Pressures	E/e´> 9	1

*Adapted from Reddy YNV et al.*^*5*^*Circulation. 2018; 138:861-870. BMI: body mass index; PASP: pulmonary artery systolic pressure.*

**Table 1.2:** HFA PEFF score HFpEF diagnosis

DOMAIN	MAJOR CRITERIA (2 points)	MINOR CRITERIA (1 point)
FUNCTIONAL	e’ septal < 7 or e’ lateral < 10 or E/e’ > 15 or RT velocity > 2.8 m/s (PSAP > 35 mmHg)	E/e’: 9-14 or GLS < 16%
MORPHOLOGIC	LA Vol index > 34 mL/m^2^ or LVMI > 149/122 g/m^2^ (H/M) and RWT > 0.42	LA Vol index: 29 - 34 mL/m^2^ or LVMI > 115/95 g/m^2^ (H/M) or RWT > 0.42 or left ventricular wall thickness ≥ 12 mm
BIOMARKER (sinus rhythm) BIOMARKER (atrial fibrilation)	NT-proBNP > 220 pg/mL or BNP > 80 pg/mL NT-proBNP > 660 pg/mL or BNP > 240 pg/mL	NT-proBNP: 125 - 220 pg/mL or BNP 35 - 80 pg/mL NT-proBNP: 365 - 660 pg/mL or BNP: 105 - 240 pg/mL

*Adapted from Pieske B et al.*^*7*^*Heart Failure Association (HFA) of the European Society of Cardiology (ESC). Eur J Heart Fail. 2020; 22:391-412.*

*BNP: B-type natriuretic peptide; GLS: global longitudinal systolic strain; LAVI: left atrial volume index; LV: left ventricular; m: men / w: women; NT-proBNP: N-terminal B-type natriuretic peptide; PW: posterior wall; RWT: relative wall thickness; TR velocity: tricuspid regurgitation velocity. BNP: B-type natriuretic peptide; GLS: global longitudinal systolic strain; LAVI: left atrial volume index; LV: left ventricular; m: men / w: women; NT-proBNP: N-terminal B-type natriuretic peptide; PW: posterior wall; RWT: relative wall thickness; TR velocity: tricuspid regurgitation velocity.*

**Table 1.3:** HFpEF diagnosis recommendations

Recommendations	Class	LE	Comments	Table 2018	Ref.
Natriuretic peptides for HFpEF screening.	I	B	**NEW:** The wide variation in serum levels of natriuretic peptides in this population and the conditions that modify its accuracy, such as atrial fibrillation and obesity, should be considered.	New	3, 4
Comprehensive echocardiogram for diagnosis confirmation.	I	B	**NEW:** Echocardiogram with presentation of Doppler indices to estimate pulmonary and diastolic pressures, as well as cardiac mass and volume indices indexed to body surface.	New	4, 5
H_2_FPF or HFA PEFF diagnostic scores to improve diagnostic accuracy for suspected cases of HFpEF.	IIa	B	**NEW:** Scores validated using retrospective cohorts.	New	6–8
Assessment of diastolic function during stress by echocardiogram or invasive hemodynamic monitoring in cases of intermediate probability in the H2FPF or HFA PEFF scores.	IIb	B	**NEW:** Scores validated using retrospective cohorts.	New	9,10
The initial strategy for HFpEF diagnosis is to determine the pretest probability of HF through the use of clinical findings associated with supplementary tests, such as electrocardiograms, chest X-rays, echocardiograms, and natriuretic peptides, if available. When interpreting natriuretic peptide test results, it is important to consider there is a wide range of serum levels in this population and that, in the presence of atrial fibrillation (AF), higher thresholds need to be considered.^3^^,^^4^ If HF is a plausible diagnosis, it is reasonable to apply H_2_FPEF^5^^,^^6^ and HFA PEFF^7^ scores (the first with clinical and echocardiography data, the latter with comprehensive echocardiography and natriuretic peptide data), which have been validated in international populations^6^^,^^8^ to determine high, intermediate and low probability. In patients with low probability of HFpEF, the objective pursuit of other etiologies for dyspnea is suggested. In individuals with intermediate probability, recent studies have shown that data on diastolic function during stress may identify patients with abnormal responses, representing a noninvasive (echocardiographic diastolic assessment)^9^ or invasive (pulmonary artery catheter) diagnostic strategy.^10^ The scoring systems above require comprehensive echocardiograms; in other words, the examination should provide information on diameters, left atrial volume, flow Doppler, tissue Doppler (septal and/or lateral e’), and, if possible, myocardial strain and strain rate data·5					

*AF: atrial fibrillation; HF: heart failure; HFpEF: heart failure with preserved ejection fraction*

### 1.2. Treatment for Heart Failure with Preserved Ejection Fraction (HFpEF)

To date, there is no specific intervention to reduce cardiovascular events in patients with HFpEF. Clinical trials assessing the use of angiotensin-converting enzyme II inhibitors (ACEIs), angiotensin II receptor blockers (ARBs), angiotensin II receptor-neprilysin inhibitors (ARNIs), and spironolactone were neutral in terms of risk reduction compared to placebo for patients with HFpEF.^11^–^14^ Post-hoc analysis according to LV ejection fraction (LVEF) has consistently shown lack of benefit among subgroups with higher LVEF (above 50%). A meta-analysis of randomized controlled trials of beta-blockers provides similar findings.^13^ Therefore, the 2018 guideline recommendations for pharmacological treatment of HFpEF stand, including the use of diuretics for congestion and the treatment of comorbidities such as myocardial ischemia, atrial fibrillation, and hypertension, to reduce symptoms and potentially reduce the progression of HFpEF.^15^ Hence, it is essential to investigate potentially reversible conditions associated with ‘secondary’ HFpEF, such as infiltrative and restrictive cardiomyopathies, in addition to considering alternative causes of exercise intolerance.

### 1.3. Treatment for Heart Failure with mildly reduced Ejection Fraction (HFmrEF) (Table 1.4)

**Table 1.4:** HFmrEF treatment recommendations

Recommendations	Class	LE	Comments	Table 2018	Ref.
Bisoprolol, carvedilol or metoprolol succinate for HFmrEF patients in sinus rhythm to reduce morbidity and mortality.	IIa	A	**NEW:** Currently available data indicate that the response of patients with HFmrEF to the treatment for HF is similar to that of patients with HFrEF.	New	13
ACEI or ARB to reduce morbidity and mortality	IIa	B		New	11
Spironolactone to reduce morbidity and mortality	IIa	B		New	12
Sacubitril-valsartan, instead of ACEI (or ARB), for symptomatic patients using guideline-directed medical therapy (GDMT) including triple therapy to reduce hospitalization.	IIa	B		New	14
Despite the absence of studies assessing therapeutic interventions directed specifically to patients with heart failure with mildly reduced ejection fraction (HFmrEF), secondary analyses of clinical trials with patients with HFrEF and HFpEF indicate HFmrEF patients (LVEF 41-49%) may benefit from interventions currently indicated for HFrEF patients (LVEF ≤ 40%). A meta-analysis of 11 randomized controlled trials found beta-blockers are associated with lower mortality in patients with HFmrEF and in sinus rhythm.^13^ A subanalysis of the TOPCAT trial identified the beneficial effect of spironolactone for cardiovascular mortality in patients with LVEF ranging between 44 and 50 percent;^12^ a subanalysis of the CHARM trial found benefits from candesartan on combined endpoints of cardiovascular mortality and hospitalization for patients with LVEF from 40 to 49%.^11^ The combined analysis of the *PARAGON-HF (Angiotensin–Neprilysin Inhibition in Heart Failure with Preserved Ejection Fraction)* and *PARADIGM-HF (Prospective Comparison of ARNi with ACE-I to Determine Impact on Global Mortality and Morbidity in Heart Failure)* trials suggest that sacubitril-valsartan is associated with lower hospitalizations for mildly reduced LVEF levels, and that the effect is more intense for female patients with higher LVEF levels.^14^					

*ACEI: angiotensin-converting enzyme inhibitors; ARB: angiotensin recepter blockers; HFpEF: heart failure with preserved ejection fraction;*^*15*^*HFmrEF: heart failure with mildly reduced ejection fraction; HFrEF: heart failure with reduced ejection fraction; LVEF: left ventricular ejection fraction.*

### 1.4 Treatment for Heart Failure with Improved Ejection Fraction (HFimpEF) (Table 1.5)

**Table 1.5:** HFimpEF treatment recommendations

Recommendations	Class	LE	Comments	Table 2018	Ref.
Continuing disease-modifying drug therapy used in treating HFrEF in improved dilated cardiomyopathy.	I	B	**NEW:** Indication supported by a randomized multicenter trial with limited sample and surrogate endpoints.	New	16
Advancements in the treatment of HF with reduced ejection fraction (HFrEF) has led to improved left ventricular ejection fraction (LVEF) and a reduction in left ventricle size of about 40 percent in patients, depending on etiology.^17^ In that setting, the 2013 ACC/AHA guideline for the management of HF created the term “HF with improved or recovered LVEF,” establishing a new classification for patients with prior HFrEF who improved their LVEF at rates above 40%.^18^ More recently, Halliday BP et al.^16^tested the safety of withdrawing HF medication in a small group of patients with recovered dilated cardiomyopathy in an unblinded but randomized and multicenter pilot trial. The inclusion criteria were: prior diagnosis of dilated cardiomyopathy with LVEF 40 percent or lower; absence of heart failure symptoms; treatment with loop diuretics and disease-modifying drug therapy; current LVEF of 50% or greater; left ventricular end diastolic volume indexed to normal body surface and NT-proBNP below 250 pg/mL. Patients were randomly assigned to the medication withdrawal group for 6 months and the primary endpoint was a combination of a reduction in LVEF, LV dilation, and return of HF symptoms. After 6 months of follow-up, 44% of patients assigned to the treatment withdrawal group met some of the criteria of the primary endpoint, compared to no members of the treatment continuation group, recording a 45.7% estimated event rate (95% CI 28.5–67.2; p = 0.0001). Despite a small sample size and a suboptimal design, this is the best evidence available in the HFimpEF population, suggesting that continuation of drugs in this context is the best strategy, at least until the publication of a more robust study.					

*This guideline uses the denominations and definitions according to the new universal classification of heart failure.*^*18*^*HFrEF: heart failure with reduced ejection fraction; LVEF: left ventricular ejection fraction; NT-proBNP: N-terminal portion of B-type natriuretic peptide.*

## 2. Innovations in Cardiac Amyloidosis

We have recently seen great advances in cardiac amyloidosis, with a profound transformation of its clinical and epidemiological significance and the development of specific treatments. Evidence suggests that cardiac amyloidosis is not a rare disease, but rather a largely underdiagnosed condition, now considered a relatively common and treatable cause of HFpEF, particularly transthyretin amyloidosis (ATTR) in its wild type (ATTR-wt), of which diagnosis has increased expressively.^19^–^22^

It is a multisystemic disease caused by the tissue deposition of insoluble fibrillary proteins that lose their conformation, leading to organ dysfunction, including the heart. Over 30 types of amyloidogenic proteins have been described,^23^ with two of them responsible for 95% of all cases of cardiac involvement: light-chain amyloidosis (AL), related to monoclonal production of immunoglobulins due to plasma cell dyscrasias; and transthyretin amyloidosis (ATTR), caused by misfolded transthyretin, a plasma protein that transports thyroxine and retinol and is secreted mainly by the liver. ATTR can be secondary to an abnormal (mutant or variant) protein (ATTRm) or to the wild-type form (ATTRwt), caused by post-transcriptional modification or by chaperone-related mechanisms, both associated with aging.

AL incidence ranges from 6 to 10 million people/year and, until recently, was considered the primary cause of cardiac amyloidosis.^24^ However, with the development of noninvasive diagnosis techniques and effective treatment, the diagnosis of ATTR, especially of ATTRwt, has increased significantly.^19^ Studies demonstrate ATTR in up to 13% of patients with HFpEF and left ventricular wall thickening greater than 12 mm,^20^ with up to 25% of necropsies of very elderly people showing TTR in the heart.^22^ ATTRm is an autosomal dominant condition, with more than 130 mutations described and several phenotypes of neurological and cardiac impairment.

### 2.1. When to Suspect Amyloidosis

Considering that ATTR, particularly ATTRwt, is more prevalent than previously expected, it is important to suspect it in the presence of clinical clues for further diagnostic investigation (Table 2.1). ATTR commonly manifests as infiltrative restrictive cardiomyopathy, with ventricular wall thickening, diastolic dysfunction, and conduction disorders. In certain clinical contexts, a differential diagnosis with hypertrophic cardiomyopathy, HFpEF^25^, advanced atrioventricular blocks and atrial arrhythmias with no apparent cause are necessary. The simultaneous finding of ATTRwt and calcific aortic stenosis may cause severe ventricular hypertrophy and can present as low-flow, low-gradient aortic stenosis. In addition, some multisystemic manifestations may raise suspicion of ATTR: bilateral carpal tunnel syndrome, biceps tendon rupture, orthostatic hypotension, spinal canal stenosis, digestive problems, and intolerance to antihypertensive medications.^26^ Family history is very important in the hereditary forms of amyloidosis, carrying a worse prognosis than the wild-type form.

**Table 2.1:** Clinical clues for amyloidosis diagnosis

**History and physical examination**
HFpEF, particularly in elderly men (over 65)
Intolerance to ACEI/ARB/ARNI and/or beta-blockers
Bilateral carpal tunnel syndrome
Spinal canal stenosis
Rupture of the biceps tendon
Unexplained peripheral neuropathy, particularly when associated with autonomic dysfunction
Periorbital ecchymosis
Macroglossia
Clues from Imaging Examinations and Laboratory Tests
Grade 2-3 myocardial uptake in Tc-99m pyrophosphate scintigraphy
Infiltrative phenotype on echocardiogram, with biventricular hypertrophy, pericardial effusion, valve thickening, and interatrial septum thickening
Longitudinal strain rate reduction that spares the apical region (apical sparing)
Restrictive abnormality of ventricular filling with right ventricular wall thickening
Cardiac magnetic resonance imaging showing late gadolinium enhancement with diffuse subendocardial or transmural pattern, increased extracellular volume
Proteinuria
Combined Clues
Heart failure with unexplained LV wall thickening and a nondilated ventricular cavity (intraventricular septum larger than 12 mm)
Concentric left ventricular hypertrophy with reduced or non-increased QRS amplitude proportional to degree of LV wall thickening
Reduced longitudinal left ventricular systolic function despite normal LVEF
Aortic stenosis with right ventricular wall thickening, particularly in paradoxical low-flow, low-gradient cases

*ACEI: angiotensin-converting enzyme II inhibitors; ARB: angiotensin II receptor blockers; ARNI: angiotensin II receptor-neprilysin inhibitors; HFpEF: heart failure with preserved ejection fraction; LV: left ventricular; LVEF: left ventricular ejection fraction.*

### 2.2. Cardiac Amyloidosis Diagnosis (Table 2.1)

When suspected, the first step in investigating cardiac amyloidosis is the search for the presence of immunoglobulin light chains for the diagnosis of AL, which requires specific treatment with chemotherapeutic agents and has a worse prognosis with delayed treatment initiation. Confirmation of AL depends on the detection of amyloid protein in the tissues involved (biopsy), but the ATTR form can be confirmed noninvasively, using cardiac scintigraphy with bone-avid radiotracers. In Brazil, Tc-99m pyrophosphate is used in the examination.

### 2.3. Diagnostic Methods

#### 2.3.1. Electrocardiogram

A low-amplitude QRS complex is a frequent finding in AL, but less prevalent in ATTR (around 30% of cases), that more commonly presents discrepancy between the magnitude of the hypertrophy on the echocardiogram and the amplitude of QRS complexes is more frequent. Atrial fibrillation and a “pseudo-infarction” pattern may also be found.

#### 2.3.2. Echocardiogram

Echocardiogram is the most important exam to raise the suspicion of CA. Suggestive findings include left ventricular wall thickening greater than 12 mm, especially in the absence of hypertension, bi-atrial enlargement disproportionate to ventricle size, atrioventricular valve and interatrial septum thickening, and increased myocardial echogenicity with a granular aspect. Myocardial longitudinal systolic strain rates may show the preservation of left ventricular apical contractility as compared to the remaining segments (apical sparing or “cherry on top” pattern) as compared to the reduced contractility in the remaining segments.^27^

#### 2.3.3. Cardiac Scintigraphy with Bone-Avid Radiotracers

Cardiac scintigraphy with bone-avid radiotracers, such as Tc-99m pyrophosphate as used in Brazil, can be used for the differential diagnosis between amyloidosis AL and ATTR, with the latter showing anomalous myocardial uptake, higher than or equivalent to bone uptake. However, cardiac uptake may occur, albeit with milder intensity, in up to 30% of AL cases. The combination of intense cardiac uptake (grades 2 or 3) and the absence of light chains in biochemical exams presents 100% specificity for ATTR, and can obviate a cardiac biopsy for the diagnosis of the disease.^19^

#### 2.3.4. Cardiac Magnetic Resonance Imaging

Cardiac magnetic resonance imaging has high sensitivity and specificity for the diagnosis and discrimination between cardiac amyloidosis and other cardiomyopathies. Amyloid deposits in the myocardium cause an increase in the distribution volume of paramagnetic contrast agent in myocardial regions where cardiomyocytes are replaced or displaced by inflammation or fibrosis, originating a diffuse subendocardial and circumferential late enhancement pattern of the left ventricle; a diffuse transmural pattern can also be found.^27^

### 2.4. Treatment of Cardiac Transthyretin Amyloidosis (ATTR-CA) (Table 2.2)

**Table 2.2:** Recommendations for specific treatment for cardiac transthyretin amyloidosis (ATTR-CA)

Recommendation	Class	LE	Comment	Table 2018	Ref.
Tafamidis 80 mg/day for treatment of patients with cardiac transthyretin amyloidosis patients in order to reduce mortality and cardiovascular hospitalizations.	I	B	**NEW:** A multicenter randomized clinical trial supports the recommendation.	New	28
Several steps of amiloid fibers formation constitute therapeutic targets in transthyretin amyloidosis (ATTR). The first disease-modifying therapy to show any evidence of benefit in patients with amyloid cardiomyopathy is tafamidis, a TTR tetramer stabilizer. Tafamidis was tested in a multicenter, placebo-controlled, randomized trial involving 441 patients with cardiac amyloidosis patients, of which 264 were assigned to receive tafamidis at doses of 20 mg or 80 mg daily (*ATTR-ACT [Tafamidis Treatment for Patients with Transthyretin Amyloid Cardiomyopathy]* study).^28^ The primary results showed that the use of tafamidis was associated with a 30% reduction in all-cause mortality (RR = 0.70 [95% CI: 0.51-0.96]) and 32% reduction of cardiovascular-related hospitalizations (RR = 0.68 [95% CI: 0.56 −0.81]), in addition to reduced worsening of functional capacity and quality of life. Based on these results, tafamidis was approved by ANVISA in Brazil for treatment of CA-ATTR, at a dose of 80 mg / day.^28^					

*ATTR: transthyretin amyloidosis; RR: relative risk*

Given its clinical and epidemiological importance, in addition to new emerging therapies for the condition, a Position Paper on Diagnosis and Treatment of Cardiac Amyloidosis will be published shortly, and should review the different aspects of the disease more broadly.

## 3. Innovations in Telemonitoring for Heart Failure (Table 3.1)

**Table 3.1:** Recommendations for telemonitoring, wearables, artificial intelligence, and machine learning in heart failure

Recommendations	Class	LE	Comment	Table 2018	Ref.
Use of telemonitoring to manage patients with chronic HF.	IIa	A	NEW: Meta-analyses show reduction in mortality rates and in hospitalizations for HF.	New	29–32
Wearables as complementary tools in the diagnosis and treatment of patients with chronic or acute HF.	IIa	B	NEW: Several observational studies show the benefits of wearables use for HF patients.	New	33, 34
Artificial intelligence use in the diagnosis, prognostic assessment, or selection of patients who can most benefit from different therapies.	IIb	B	NEW: Observational studies indicate the benefits of using Machine Learning and Artificial Intelligence in the diagnosis and prognosis of HF.	New	35
Meta-analyses involving observational and randomized trials on invasive and noninvasive distance monitoring and support has found a positive impact on the prognosis for HF patients.^29^^–^^32^ Reductions in all-cause mortality may range from 19 to 31% with telemonitoring for HF patients, while the reduction in frequency of hospitalizations for HF ranges from 27 to 39%, especially for patients in functional class (FC) III/IV, according to the New York Heart Association (NYHA). Artificial intelligence has applications in HF, either for diagnosis, prognostic assessment, telemonitoring or selection of patients who can most benefit from various therapies.^33^^,^^34^ This is possible, for instance, in distinguishing phenotypes, assigning patients in different signature profiles; more accurate diagnosis of acute HF as compared to physicians; and also helping in referral for new or established therapies, such as additional analysis of baseline ECG to identify patients who would better respond to cardiac resynchronization therapy.^35^					

*FC: functional class; HF: heart failure.*

## 4. Innovations in Cardiac Interventions

### 4.1. Percutaneous Intervention in Secondary Mitral Insufficiency (Table 4.1)

**Table 4.1:** Recommendations for percutaneous interventions in severe secondary mitral insufficiency

Recommendations	Class	LE	Comments	Table 2018	Ref.
**Percutaneous mitral valve clipping**					
**Ischemic or dilated**					
Refractory symptoms (NYHA II-IV), despite guideline-directed medical therapy and after Heart Team evaluation.	IIa	B	**NEW:** Recommendation supported by a randomized trial with mortality endpoint.	Item 11.3 (page 467)	36
We recommend optimization of guideline-directed medical therapy (GDMT), including cardiac resynchronization therapy and revascularization, when appropriate, before considering percutaneous mitral insufficiency (MI) treatment for patients with HFrEF and severe MI. The *COAPT (Transcatheter Mitral-Valve Repair in Patients with Heart Failure)* trial assessed whether the edge-to-edge device might benefit patients with moderately severe or severe secondary MI (EROA greater than or equal to 30 mm^2^ and/or regurgitation volume greater than 45 mL) with LVEF 20 to 50%, LV end-systolic diameter smaller than 7 cm and persistent symptoms, despite maximized evidence-based therapy.^36^ The 2020 Valve Disease Guidelines overlooks this distinction when selecting patients. In order to maintain linearity between the Guidelines, it remains as established in the 2020 Valve Disease Guidelines.^37^					

*HF: heart failure; HFrEF: heart failure with reduced ejection fraction; LV: left ventricle; LVEF: left ventricular ejection fraction; MI: mitral insufficiency.*

### 4.2. Atrial Fibrillation Ablation (Table 4.2)

**Table 4.2:** Recommendations for atrial fibrillation ablation in HFrEF

Recommendations	Class	LE	Comments	Table 2018	Ref.
AF ablation to reestablish sinus rhythm in symptomatic patients, intolerant or refractory to antiarrhythmic medications to reduce mortality and hospitalizations for HF.	IIa	B	2018 recommendation remains current.	Item 10.1 (page 465)	See 2018
AF ablation as an alternative to clinical treatment for selected patients with symptomatic persistent AF refractory or intolerant to at least one antiarrhythmic medication.	I	A	**NEW:** Randomized trials have shown a higher rate of success in sustaining a sinus rhythm with AF ablation, without antiarrhythmic medications side effects.	Item 10.1 (page 465)	38–43
AF ablation to promote reverse remodeling in patients with AF-induced tachycardiomyopathy if refractory to pharmacological treatment or if patient chooses ablation, regardless of symptoms.	I	B	**NEW:** A randomized trial showed AF ablation can promote reverse remodeling in patients with tachycardiomyopathy.	Item 10.1 (page 465)	39–44
In patients with HF, AF ablation is superior to medical treatment, as it is associated with improved maintenance of sinus rhythm, functional capacity and quality of life (6-minute walk test, VO_2_ max), in addition to greater reduction in biomarkers (BNP). It can be considered for selected patients with symptomatic persistent AF refractory or intolerant to at least one antiarrhythmic medication or even as initial therapy.^38^^–^^43^ Reverse remodeling was observed in several AF ablation trials, leading to increased LVEF.^38^^–^^42^^,^^44^ When the HF etiology is unknown and AF-induced tachycardiomyopathy is considered as a possible etiology, the expected increase in LVEF after ablation is even more significant.^39^^,^^44^ Studies demonstrated a reduction of 45% in hospitalizations for HF, 47/56% in all-cause mortality and 38% in mortality or hospitalization for HF.^41^^,^^42^^,^^44^ However, AF ablation success rates range from 60 to 80% in the first year and structural heart disease is a major risk factor for recurrence.^45^ Pulmonary vein isolation can be done by radiofrequency or cryoablation, and these techniques may be combined with the ablation of other substrates.					

*AF: atrial fibrillation; FC: functional class; HF: heart failure.*

## 5. COVID-19 and Heart Failure (Table 5.1)

**Table 5.1:** Recommendations for COVID-19 management in heart failure patients

Recommendations	Class	LE	Comments	Table 2018	Ref.
RT-PCR testing for SARS-CoV-2 in individuals with chronic HF and acute respiratory symptoms.	I	C	**NEW:** Editorials and society recommendations (online publication).	New	46,47
ACEI, ARB or ARNI maintenance in HF patients who develop COVID-19, in the absence of hypotension or other signs of hemodynamic impairment.	I	C	**NEW:** Controlled observational studies with large numbers of participants, but a smaller number of HF patients.	New	48–50
Outpatient follow-up of HF through remote appointments (telemedicine and telemonitoring) during the COVID-19 pandemic.	I	C	**NEW:** Experts and Society recommendations	New	51,52
Considering COVID-19 symptoms can simulate decompensated HF, RT-PCR testing for SARS-CoV-2 is recommended for patients seeking medical care in the emergency department or outpatient clinic setting.^46^^,^^47^ There is no evidence for routine discontinuation of ACE inhibitors, ARBs or ARNI in patients with symptomatic HF diagnosed with COVID-19. Decisions to add or remove these medications should be guided by standard clinical practice, and individualized treatment decisions should be made according to each patient's hemodynamic status and clinical presentation.^48^^–^^50^ Online and/or remote tools (phone calls, telemonitoring, online appointments, and video calls, among others) may be used to keep continuous care for HF patients during the COVID-19 pandemic. These actions that are useful to reduce patients' virus exposure, has being effective for usual care, and are expected to endure in the post-pandemic world. For patients with clinical instability (post-discharge for HF decompensation or recent-onset HF) and candidates for advanced HF therapies (transplantation or ventricular assistant devices), we recommend at least one in-person appointment, in between virtual visits, especially considering the pandemic tends to decrease the number of transplants performed, and to increase the waiting-list period of time.^51^^,^^52^					

*ACEI: angiotensin-converting enzyme II inhibitors; ARB: angiotensin II receptor blocker; ARNI: angiotensin II receptor-neprilysin inhibitor; HF: heart failure.*

## 6. Innovations in Advanced Heart Failure

### 6.1. Definition of Advanced Heart Failure

The natural history of HF is characterized by a progressive deterioration of cardiac function and HF symptoms. Despite advances in pharmacological treatment and the prognostic impact of implantable devices such as cardiac resynchronization therapy, HF patients may progress to a clinical condition known as advanced HF, where traditional treatment is not effective and advanced therapies are required, such as heart transplantation, mechanical circulatory support device (MCSD) or palliative care are required.

Although the expression advanced HF has been used since 2007, recent updates were described to include clinical situation that may also require advanced therapies such as HFpEF patients with severe restrictive condition, rather than limiting it to patients with HF with severely reduced ejection fraction.^1^^–^^3^^,^^4^ In this scenario, isolated severe right ventricular dysfunction and severe inoperable valvular disease as well as congenital abnormalities may also be considered causes of severe cardiac dysfunction (Table 6.1).^53^^–^^68^

**Table 6.1:** Criteria for the definition of advanced heart failure

Criteria for advanced HF
1. Persistent and severe HF symptoms (NYHA III or IV).
2. Severe ventricular dysfunction defined by: LVEF ≤ 30% or Isolated right HF or Severe inoperable valvular alterations or Congenital abnormalities Persistently elevated BNP or NT-proBNP levels and data showing severe diastolic dysfunction or structural LV abnormalities, according to the criteria for HFpEF or HFmrEF.
3. Episodes of systemic or pulmonary congestion requiring high doses of intravenous diuretics (or combinations of diuretics) or episodes of low cardiac output requiring the use of inotropes or vasoactive medications or malignant arrhythmias causing more than one unplanned visit to the emergency department or hospitalization in the last 12 months.
4. Severely reduced physical exercise capacity, with inability to perform or low capacity in the 6-minute walk test (6MWT < 300 m) or VO^2^ peak (< 12-14 mL.kg^-1^.min^-1^), of likely cardiac origin.

*Adapted from Metra M et al.*^*65*^*Eur J Heart Fail. 2007; 9(6-7): 684-94; Metra M et al.*^*66*^*Cardiac Fail Rev. 2019; Crespo-Leiro MG et al.*^*67*^*Eur J Heart Fail. 2018; 20(11): 505-35; Trusby LK et al.,*^*68*^*JACC Heart Fail. 2020; 8(7): 523-36.*

*6MWT: 6-minute walk test; BNP: B-type natriuretic peptide; HF: heart failure; HFmrEF: heart failure with mid-range ejection fraction; HFpEF: heart failure with preserved ejection fraction; LV: left ventricle; LVEF: left ventricular ejection fraction; NYHA: New York Heart Association; NTproBNP: N-terminal portion of B-type natriuretic peptide; VO_2_: oxygen consumption.*

Different societies of cardiology adopt different criteria for the condition, but all of them include the presence of persistent severe symptoms, exercise intolerance, and recurrent episodes of systemic or pulmonary congestion requiring hospitalization, as described in Table 6.2.

**Table 6.2:** Criteria proposed by various cardiology societies to identify advanced HF patients

Criterion	SBC	ACC/AHA	ESC	HFSA
Severe and persistent symptoms despite optimized therapy	✓	✓	✓	✓
Major functional limitation (NYHA III or IV functional class)	✓	✓	✓	✓
Persistent dyspnea in daily living activities		✓		
Recurring hospitalizations	✓	✓	✓	✓
Frequent unplanned visits to the emergency department	✓		✓	✓
Intolerance to maximum optimal medical therapy	✓	✓		✓
End-organ dysfunction	✓	✓		✓
Persistent hyponatremia	✓	✓		✓
Pulmonary or systemic congestion refractory to diuretics	✓	✓		✓
Frequent ICD shocks	✓	✓		✓
Cardiac cachexia	✓	✓		✓
Systolic blood pressure frequently ≤ 90 mm Hg		✓		
Persistently elevated BNP or NT-proBNP values	✓		✓	
Severe dysfunction with reduced LV ejection fraction (LVEF < 30%)	✓		✓	✓
Severe LV dysfunction with pseudonormal or restrictive pattern	✓		✓	
Elevated filling pressures (PCWP > 16 mm Hg +/- CVP > 12 mm Hg)			✓	
Low capacity in 6MWT (< 300 m) or VO_2_ peak < 12-14 mL.kg^-1^.min^-1^	✓		✓	✓
Dependence on intravenous inotropes	✓			✓
Progressive RV dysfunction and secondary PH	✓			✓

*Adapted from Metra M et al.*^*65*^*Eur J Heart Fail. 2007; 9(6-7): 684-94; Metra M et al.*^*66*^*Cardiac Fail Rev 2019; Crespo-Leiro MG et al.*^*67*^*Eur J Heart Fail. 2018; 20(11): 505-35; Trusby LK et al.*^*68*^*JACC Heart Fail. 2020; 8(7): 523-36*.

*6MWT: 6-minute walk test; ACC/AHA: American College of Cardiology/American Heart Association; BNP: B-type natriuretic peptide; CVP: central venous pressure; ESC: European Society of Cardiology; HF: heart failure; ICD: implantable cardiac defibrillator; HFSA: Heart Failure Society of America; LV: left ventricle; NT-proBNP: N-terminal portion of B-type natriuretic peptide; NYHA: New York Heart Association; PCWP: pulmonary capillary wedge pressure; PH: pulmonary hypertension; RV: right ventricle; VO_2_: oxygen consumption.*

Early recognition is decisive for the prognosis of patients with advanced HF, since it allows timely referral to a specialized center able to provide the necessary advanced therapies to manage such cases.

A particularly useful mnemonic that may help identify patients requiring referral to a HF specialist is I-NEED-HELP, which combines clinical history, hospitalizations and intolerance to medications, as well as symptoms and end-organ dysfunction. (Table 6.3)

**Table 6.3:** Warning signs in advanced HF patients

I	IV inotrope dependence
N	Persistent NYHA III/IV, persistent elevation in natriuretic peptides
E	End-organ dysfunction
E	Ejection (fraction) below 20%
D	Defibrillator shocks (recurring appropriate shock)
H	Recurring hospitalizations and emergency department visits in the last 12 months
E	Persistent edema, refractory to escalating diuretics
L	Low systolic blood pressure, persistently below 90 mm Hg
P	Progressive intolerance to optimized medical therapy

### 6.2. The Role of the Specialist in Advanced Heart Failure

As the specific profile of patients fitting the current definition of advanced HF becomes increasingly clear, there is also a need to define the importance of the specialist in advanced HF in specialized centers. These professionals must be familiar (and trained) in the care of potential heart transplant candidates and their subsequent follow-up, as well as in patients with CS. They should coordinate the work of the shock team and therefore must be familiar with the diverse and growing options for circulatory support. Finally, the advanced HF specialist should be able to understand the timing and implications of discussing palliative care and advanced directives for patients who are not eligible for heart transplantation, as well as the use of long-term devices.

### 6.3. Approach to the Advanced Heart Failure Patient (Figure 6.1)

**Figure 6.1:** Treatment algorithm for patients with advanced heart failure

*Clinical classification of patients with advanced heart failure from the Interagency Registry for Mechanically Assisted Circulatory Support (Intermacs), see Brazilian Guidelines on Chronic and Acute Heart Failure.15 Arq Bras Cardiol. 2018; Quadro 4.6 (page 505).

**Cardiogenic shock classification proposed by the Society for Cardiovascular Angiography and Interventions (SCAI). Stage A: at risk of shock; Stage B: beginning shock; Stage C: classic shock; Stage D: deteriorating shock; Stage E: extremis. Adapted from Baran DA et al.^*58*^*Catheter Cardiovasc Interv. 2019; 94(1): 29-37.*

*FC: functional class; HF: heart failure; ICD: implantable cardiac defibrillator; IVAD: implantable ventricular assist device; MCSD: mechanical circulatory support device; NYHA: New York Heart Association; VA-ECMO: venoarterial extracorporeal membrane oxygenation.*

### 6.4. Innovations in Managing Congestion in Patients with Advanced Heart Failure (Table 6.4)

**Table 6.4:** Ambulatory monitoring of congestion in heart failure

Recommendations	Class	LE	Comments	Table 2018	Ref.
Invasive remote monitoring of congestion using an implantable, wireless pulmonary artery pressure sensor to reduce hospitalizations and mortality in outpatient HFrEF patients.	IIa	B	**NEW:** The current recommendation reflects data from small randomized trials and real-world studies, with impact in reducing hospitalizations and mortality.	New	30,53–57
While there has been relatively little innovation in the management of congestion in advanced HF, recent evidence suggests a potential benefit of remote monitoring, impacting the prognosis for HF patients. Studies of non-invasive home telemonitoring have shown improvements in hospital length of stay and all-cause mortality.^30^ Similar results were observed with the implantable CardioMEMS™ HF System, which provides direct pulmonary artery pressure monitoring. The impact of invasive monitoring was tested in the *CHAMPION (Wireless pulmonary artery haemodynamic monitoring in chronic heart failure: a randomised controlled trial)* trial, which involved outpatients with HF (FC III, NYHA) and demonstrated a 28% reduction in hospitalizations for HF. Among patients receiving at least two medications from standard HF therapy, invasive monitoring was associated with a 57% reduction in mortality.^53^ The CardioMEMS™ proved to be safe and effective in “real-world”, ^54^ as well as in cost-effectiveness studies.^55^ The data were recently replicated in a study conducted by multiple European centers^56^ and in an open multicenter prospective study of 1200 FC III patients, which found a significant decrease in hospitalizations for HF with low rates of complications associated with the implantable monitor over the one-year follow-up period.^57^ This is a promising strategy, with potential to be translated into clinical practice.					

*FC: functional class; HF: heart failure; NYHA: New York Heart Association; PAP: pulmonary artery pressure.*

### 6.5. Current Classification of Cardiogenic Shock

In 2019, the Society for Cardiovascular Angiography and Interventions (SCAI) proposed a new classification for cardiogenic shock (CS) in order to make it easier to identify the various stages of clinical deterioration as well as the need for more intensive treatment.^58^^,^^59^ The 5-stage classification incorporates signs of tissue hypoperfusion and organic dysfunction, offering a simple hemodynamic definition and granularity to the INTERMACS (Interagency Registry for Mechanically Assisted Circulatory Support) classification (Figure 6.2, Table 6.5).

**Figure 6.2:** *Society for Cardiovascular Angiography and Interventions (SCAI) classification of cardiogenic shock*.

*Adapted from Baran DA et al.*^*58*^*SCAI clinical expert consensus statement on the classification of cardiogenic shock. Catheter Cardiovasc Interv. 2019; 94(1): 29-37.*

*AMI: acute myocardial infarction; CPR: cardiopulmonary resuscitation; CRA: cardiorespiratory arrest; CS: cardiogenic shock; ECMO: extracorporeal membrane oxygenation; HF: heart failure.*

**Table 6.5:** Descriptors of shock stages: physical exam, biochemical markers, and hemodynamics

Stage of CS	Bedside findings	Biomarkers	Hemodynamics
**A (at risk)**	Normal JVP Lung sounds clear Dry-warm profile Strong distal pulses Normal mentation	Normal labs Normal renal function Normal lactate	SBP ≥ 100 mm Hg (or normal for patient) If PAC: • CI ≥ 2.5L/min/m^2^ • CVP < 10 mm Hg • SvO_2_ ≥ 65%
**B (beginning)**	High JVP Rales in lung fields Dry-warm profile Strong distal pulses Normal mentation	Normal lactate Minimal renal dysfunction Elevated BNP	SBP < 90 OR MAP < 60 OR > 30 mm Hg drop from baseline HR ≥ 100 bpm If PAC: CI ≥ 2.2 L/min/m^2^ SvO_2_ ≥ 65%
**C (classic)**	***May include any of:*** Looks unwell Panicked Ashen, mottled, dusky Volume overload Extensive rales Kilip class 3 or 4 BiPap or mechanical ventilation Cold, clammy Acute alteration in mental status Urine output < 30 mL/h	***May include any of***: Lactate ≥ 2 mmol/L Creatinine doubling OR > 50% drop in GFR Altered liver enzymes Elevated BNP	***May include any of:*** SBP < 90 OR MAP < 60 OR >30 mm Hg drop from baseline. Pressor drugs and/or devices used to maintain BP PAC: • CI < 2.2L/min/m^2^ • PCWP > 15 mm Hg • CVP/PCWP ≥ 0.8 • PAPi < 1.85 • CPO ≤ 0.6W
**D (deteriorating)**	**May include any of the findings from stage C**	**Any of stage C and deteriorating.**	**Any of stage C and:** Requiring multiple pressors and/or addition of mechanical circulatory support devices to maintain perfusion
**E (extremis)**	Near pulselessness Circulatory collapse Mechanical ventilation Defibrillator used	CRA (A-modifier*) pH ≤ 7.2 Lactate ≥ 5 mmol/L	Inaudible SBP / CRA VTWP or refractory VT/VF Hypotension despite maximal support

* *The modifier (A) is used to describe patients who have gone into cardiac arrest, regardless of duration.*

*Adapted from Baran DA et al.*^*58*^*SCAI clinical expert consensus statement on the classification of cardiogenic shock. Catheter Cardiovasc Interv. 2019; 94(1):29-37.*

*AMI: acute myocardial infarction; BiPap: bi-level positive airway pressure; BNP: B-type natriuretic peptide; CI: cardiac index; CPO: cardiac power output; CS: cardiogenic shock; CVP: central venous pressure; GFR: glomerular filtration rate; HF: heart failure; HR: heart rate; JVP: jugular venous pressure; MAP: mean arterial pressure; PAC: pulmonary artery catheter; PAPi: pulmonary artery pulsatility index; PCWP: pulmonary capillary wedge pressure; SBP: systolic blood pressure; SvO2: mixed venous oxygen saturation; VF: ventricular fibrillation; VT: ventricular tachycardia; VTWP: ventricular tachycardia without a pulse.*

Stage A includes patients at risk of cardiogenic shock, while stages B through E describe progressive stages of conventional cardiogenic shock. The difference between stages B and C is the presence of hypoperfusion, present in stages C and above. Stage D indicates initial cardiogenic shock management measures were not enough to restore hemodynamic stability or tissue perfusion within at least 30 minutes of observation, while stage E characterizes extreme cases, where patients present as hemodynamically unstable and frequently in circulatory collapse. Patients in SCAI stages D and E have higher mortality rates and may benefit from early referral to specialized centers, where more advanced modes of circulatory support may be available.^59^

### 6.6. Applicability of Pulmonary Artery Catheters in Advanced Heart Failure (Table 6.6)

**Table 6.6:** Recommendations for pulmonary artery catheter use in patients with advanced HF

Recommendations	Class	LE	Comments	Table 2018	Ref.
In patients with advanced HF, heart transplantation candidates or receiving mechanical circulatory support.	I	B	2018 recommendation remains current.	Item 2.2.6. (page 495)	See 2018
To help treatment and hemodynamic support for patients with HF refractory to standard treatment or patients with cardiogenic shock.	IIa	B	**MODIFIED:**New evidence supports the change in class of recommendation.	Item 2.2.6. (page 495)	60–61
The use of a pulmonary artery catheter (PAC) in hemodynamic monitoring for patients hospitalized for refractory HF remains controversial.^62^^,^^63^ In 2005, the *ESCAPE (Evaluation Study of Congestive Heart Failure and Pulmonary Artery Catheterization Effectiveness)* trial found no benefits from the routine use of PACs in managing decompensated HF patients without CS.^64^ However, recent advances in the field of mechanical circulatory support devices (MCSDs) have prompted the development of algorithms to manage CS guided by PAC parameters. Early recognition, identification of the shock subtype and understanding of the expected impact of each type of device on hemodynamic parameters such as cardiac output, pulmonary capillary wedge pressure (PCWP), central venous pressure (CVP), and mean arterial pressure (MAP) allow choosing the most suitable MCSD for each stage of CS (Figure 6.1). In addition, the information obtained via PAC assist the phenotype characterization of CS into predominantly left ventricular shock (CPO < 0.6 W, PAPi > 1, CVP < 15 mm Hg and PCWP > 15 mm Hg), right ventricular shock (CPO < 0.6 W, PAPi <1, CVP > 15 mm Hg and PCWP < 15 mm Hg) or biventricular shock (CPO < 0.6 W, PAPi < 1, CVP > 15 mm Hg and PCWP > 15 mm Hg).^60^^,^^65^^–^^68^ Recently, in one of the first studies by the Cardiogenic Shock Working Group (CSWG), Garan et al.^61^ evaluated the association between CS management guided by CAP parameters and hospital mortality in 1,414 patients with CS, most with indication for MCSD use and in stage D of the SCAI classification. CS management guided by PAC parameters obtained before implanting a MCSD was associated with a significant decrease in mortality, especially in the more advanced stages of CS (stages D or E of the SCAI classification).^61^ It should be emphasized that the PAC is a diagnostic tool, not a therapeutic one, and its effectiveness depends on clinical decisions taken by the team involved in managing the CS.					

*CPO: cardiac power output; CS: cardiogenic shock; CVP: central venous pressure; HF: heart failure; MAP: mean arterial pressure; MCSD: mechanical circulatory support device; PAC: pulmonary artery catheter; PAPi: pulmonary artery pulsatility index; PCWP: pulmonary capillary wedge pressure.*

### 6.7. Innovations in Short-Term Circulatory Support Devices in Advanced Heart Failure (Table 6.7)

**Table 6.7:** Recommendation for left ventricular venting in patients receiving extracorporeal membrane oxygenation (ECMO)

Recommendations	Class	LE	Comments	Table 2018	Ref.
Consider strategies for left ventricular venting in patients receiving mechanical circulatory support via peripheral venoarterial ECMO, evidence of ventricular distension associated with severe hypocontractility and pulmonary congestion.	IIa	C	**NEW:** The current recommendation reflects data from observational studies and meta-analyses.	New	69–73
The use of peripheral venoarterial extracorporeal membrane oxygenation (ECMO) is characterized by an increase in LV afterload caused by blood flow from the arterial return cannula, which can worsen cardiac hypocontractility, causing ventricular distension and pulmonary congestion. In many cases, the reduction in ECMO flow combined with inotropic therapy may be sufficient to decompress the LV.^74^ However, in refractory cases, other methods of venting may be used, including atrial septostomy; surgical implantation of a transapical catheter; percutaneous pulmonary artery venting through the jugular vein; and mechanical circulatory support device (MCSD), such as the intra-aortic balloon pump (IABP), Impella^®^, or CentriMag^®^. In observational studies, LV venting has been associated with reduced mortality, increased myocardial recovery, and shorter weaning time from ECMO in patients with CS treated with peripheral venoarterial ECMO.^69^^–^^72^ Each venting technique presents inherent risks that must be considered individually according to the etiology of the underlying disease, limitations of the access site, presence of coagulopathies, availability of MCSDs and experience of each center.^75^ Despite known limitations, IABPs remain the most commonly used devices, with a recent meta-analysis suggesting lower risk of complications such as stroke, peripheral ischemia, and hemolysis from decompression by IABP as compared to other methods, at the cost of increased bleeding.^73^ However, no randomized clinical trial has been conducted to date to establish the ideal LV venting method, and prospective studies are needed. There is also no consensus on whether LV venting should be performed preventively or as a rescue measure. Known indications for LV venting include elevated PCWP, distended and hypocontractile LV, LV with echocardiographic evidence of blood stasis, decreased aortic valve opening during the cardiac cycle, hypoxemia, progressive pulmonary edema, and refractory ventricular arrhythmia.					

*CS: cardiogenic shock; ECMO: extracorporeal membrane oxygenation; LV: left ventricle; MCSD: mechanical circulatory support device; PCWP: pulmonary capillary wedge pressure.*

### 6.8. Innovations in Palliative Care for Advanced Heart Failure (Table 6.8)

**Table 6.8:** Outpatient use of intravenous inotropes in patients with advanced HF who are not eligible for heart transplantation or mechanical circulatory support devices

Recommendations	Class	LE	Comments	Table 2018	Ref.
Continuous outpatient intravenous inotrope therapy as palliative care for symptom control in advanced HF patients who are not eligible for mechanical circulatory support devices or heart transplantation.	IIb	C	**NEW:** The current recommendation reflects data from studies with limitations in design and execution.	New	76–78
Intermittent use of inotropes or inodilator to improve symptoms in advanced HF patients or palliative care in patients without other advanced therapy options.	IIb	B	**NEW:** New evidence from moderate-quality meta-analysis and RCT support the recommendation.	New	79
The evidence assessing the risks and benefits of palliative care with intravenous inotrope therapy on an outpatient basis for patients with advanced HF is limited, consisting primarily of observational studies without a control group. Meta-analyses of small randomized controlled trials and heterogeneous observational studies suggest a potential clinical benefit of continuous or intermittent outpatient inotrope therapy for patients with advanced HF who are not eligible for an MCSD or heart transplantation.^76^^–^^78^ Benefits include relief of symptoms and lower readmission rates. However, the need for a central catheter for continuous infusion of inotropes is associated with greater special care and risk of infections. The *LION-HEART (Efficacy and safety of intermittent intravenous outpatient administration of levosimendan in patients with advanced heart failure)* pilot trial randomly assigned 69 patients with advanced HF to either placebo or intermittent levosimendan at a dosage of 0.2 ¼g/kg/min for 6 hours every 2 for 12 weeks and demonstrated the benefit of inotropic therapy in relation to lower plasma NT-proBNP levels, higher quality of life scores, and lower readmission rates, with no difference in rates of adverse events between groups.^79^ To date, there are no cost-effectiveness studies evaluating the impact of outpatient inotropic infusion as palliative therapy for patients with advanced HF.					

*HF: heart failure; NT: N-terminal portion of B-type natriuretic peptide; RCT: randomized controlled trial*

## 7. Treatment of Heart Failure with Reduced Ejection Fraction (HFrEF)

### 7.1. Previously Consolidated Pharmacological Strategies for Treatment of Heart Failure with Reduced Ejection Fraction (HFrEF) (Table 7.1)

**Table 7.1:** Recommendations for pharmacological treatment of HFrEF previously consolidated in 2018

Recommendations	Class	LE	Comments	Table 2018	Ref.
Bisoprolol, carvedilol or metoprolol succinate for symptomatic LV dysfunction to reduce morbidity and mortality.	I	A	2018 recommendation remains current.	Item 7.2 (page 457)	See 2018
ACEI for symptomatic LV dysfunction to reduce morbidity and mortality.	I	A	2018 recommendation remains current.	Item 7.1 (page 456)	See 2018
ARB for symptomatic LV dysfunction (for those intolerant to ACEI due to coughing/angioedema) to reduce morbidity and mortality.	I	A	2018 recommendation remains current.	Item 7.1 (page 456)	See 2018
Mineralocorticoid receptor antagonists for symptomatic LV dysfunction, associated with standard treatment with ACEI/ARB/ARNI and BB, to reduce morbidity and mortality.	I	A	**MODIFIED:** The use of mineralocorticoid receptor antagonists is justified for patients using ACEI/ARB as well as ARNI.	Item 7.3 (page 457)	80–84
Sacubitril-valsartan, instead of ACEI (or ARB), for patients with symptomatic LV dysfunction, already receiving optimal medical therapy for HF with triple therapy to reduce morbidity and mortality.	I	B	2018 recommendation remains current.	Item 7.4 (page 458)	See 2018
Hydralazine and nitrate combination for symptomatic systolic dysfunction, NYHA II-IV, with contraindication for ACEI/ARB (renal failure and/or hypercalcemia) regardless of race or for self-declared black patients with symptomatic systolic dysfunction, NYHA III-IV, despite optimized therapy.	I	B	2018 recommendation remains current.	Item 7.7 (page 459)	See 2018
Ivabradine for symptomatic LV dysfunction in patients with optimal medical therapy for HF, sinus rhythm, and HR above 70 bpm to reduce hospitalization, cardiovascular death, and HF death.	IIA	B	2018 recommendation remains current.	Item 7.5 (page 458)	See 2018
Digoxin for symptomatic LV dysfunction despite optimal medical therapy for HF, to reduce symptoms and hospitalizations.	IIA	B	2018 recommendation remains current.	Item 7.6 (page 458)	See 2018
Loop diuretic for congestion control.	I	C	2018 recommendation remains current.	Item 7.7 (page 459)	See 2018
Thiazide diuretic, associated with loop diuretic for persistent congestion.	I	C	2018 recommendation remains current.	Item 7.7 (page 459)	See 2018
In recent decades, advances in pharmacological treatment and in the use of implantable devices have changed the prognosis of HFrEF patients.^80^^–^^91^ However, there is still a high risk of morbidity and mortality, even with the adoption of optimal medical therapy. In this new era, drugs acting on various pathophysiological mechanisms of HF have emerged to supplement the inhibition of the neurohormonal system. It should be noted that the benefits observed with the new drugs add to the optimal medical therapy, highlighting the need to maintain triple therapy, including beta-blockers, renin-angiotensin-aldosterone system (RAAS) blockers, and mineralocorticoid antagonists. Once triple therapy has been initiated and disease-modifying new therapies (with proven benefits in reducing cardiovascular death, all-cause mortality, and hospitalizations for HF) added, we can also include medications impacting morbidity. The choice of additional therapies should take into consideration each patient's profile.					

*ACEI: angiotensin-converting enzyme II inhibitors; ARB: angiotensin II receptor blockers; ARNI: angiotensin II receptor-neprilysin inhibitors; HF: heart failure; HFrEF: heart failure with reduced ejection fraction; HR: heart rate; LV: left ventricle; RAAS: renin-angiotensin-aldosterone system.*

### 7.2. Sacubitril-Valsartan (Table 7.2)

**Table 7.2:** Recommendations for the use of sacubitril-valsartan in HFrEF patients

Recommendations	Class	LE	Comment	Table 2018	Ref.
Sacubitril-valsartan, instead of ACEI/ARB, for symptomatic LV dysfunction, patients with optimal medical therapy for HF with triple therapy to reduce morbidity and mortality.	I	B	2018 recommendation remains current.	Item 7.4 (page 458)	See 2018
Sacubitril-valsartan, as initial treatment for symptomatic chronic HF, may be considered instead of ACEI or ARB.	IIa	C	**NEW:** Analysis of subgroups of randomized and non-randomized trials have found it safe for patients without prior use of ACEI/ARB.	New	84,92,93
Sacubitril-valsartan, instead of ACEI/ARB, may be considered for hospitalized patients with decompensated HF.	IIa	B	**NEW:** Randomized trial using surrogate endpoint (reduction of biomarkers) supports the new recommendation.	New	84,92,94
The *PARADIGM-HF* trial investigated the effects on morbidity and mortality in HFrEF patients of attenuating the deleterious effects of angiotensin II associated with enhancing the protective effect of endogenous natriuretic peptides through the inhibition of neprilysin (an enzyme responsible for the degradation of BNP) using a new medication class, the angiotensin II receptor-neprilysin inhibitor (ARNI), of which the molecule currently available is sacubitril-valsartan, as compared to enalapril.^83^ The trial included 8,442 patients with symptomatic outpatient HFrEF in an optimized clinical therapy regimen with persistent LVEF ≤ 40%, elevated plasma natriuretic peptide levels, and estimated creatinine clearance ≥ 30 mL/min/1.73 m^2^. In this population, sacubitril-valsartan was associated with a 21% decrease in hospitalizations for worsening HF, 20% decrease in cardiovascular death, 20% decrease in sudden death, and 16% decrease in overall mortality when compared to enalapril. Based on the results from the *PARADIGM-HF* trial, we recommend replacing ACEI/ARB with sacubitril-valsartan in HFrEF patients whose symptoms persist even after the use of optimized doses of neurohormonal blockers. More recently, the *PIONEER-HF (Angiotensin–Neprilysin Inhibition in Acute Decompensated Heart Failure)* trial compared sacubitril-valsartan (n = 440) to enalapril (n = 441) in patients hospitalized for decompensated HF, where the primary outcome was the time-averaged proportional change in the NT-proBNP concentration from baseline through weeks 4 and 8.^84^ The results show a significant decrease in NT-proBNP, higher with sacubitril-valsartan than with enalapril, and the reduction was already noticeable after the first week of treatment, regardless of prior HF history and/or use of ACEIs or ARBs.^94^ The side effects were similar for both groups, including hypercalcemia, renal dysfunction, and hypotension. In an open analysis, at the end of 8 weeks (*PIONEER-HF extended*) where all patients received sacubitril-valsartan for an additional 4 weeks, there was a significant decrease in NT-proBNP in the enalapril group after initiating sacubitril-valsartan use.^92^ Another prospective observational study, the *TRANSITION (Initiation of sacubitril/valsartan in haemodynamically stabilised heart failure patients in hospital or early after discharge) Trial*,^93^ initiated sacubitril-valsartan in 1,002 hemodynamically stabilized HF patients in hospital or early after discharge and found it to be safe and well tolerated, with half of patients reaching the target dose within 10 weeks and few adverse events.^93^ These results suggest that the use of sacubitril-valsartan is safe in hemodynamically stabilized patients with acute HF; extrapolating the results of the *PARADIGM-HF* trial, sacubitril-valsartan may be considered for treatment of patients hospitalized for decompensated HF instead of ACEI/ARB. The results from these recent trials also indicate the safety and tolerability of initiating treatment with sacubitril-valsartan instead of ACEIs/ARBs in HFrEF patients, which made up 34% of the sample in the *PIONEER-HF* trial and 29 of patients in the *TRANSITION* trial.^84^^,^^92^^,^^93^ Taken as a whole, these data suggest initiating sacubitril-valsartan for patients with no prior treatment with ACEIs/ARBs and during episodes of HF decompensation is reasonably safe. Long-term and outcome data on this form of intervention, including mortality rates, are not yet available.					

*ACEI: angiotensin-converting enzyme II inhibitors; ARB: angiotensin II receptor blockers; ARNI: angiotensin II receptor-neprilysin inhibitors; HF: heart failure; HFrEF: heart failure with reduced ejection fraction; HR: heart rate; LV: left ventricle; LVEF: left ventricular ejection fraction; RAAS: renin-angiotensin-aldosterone system.*

### 7.3. Sodium-glucose Cotransport 2 (SGLT2) Inhibitors (Table 7.3)

**Table 7.3:** Recommendations for use of SGLT2 inhibitors in the treatment of HFrEF patients

Recommendations	Class	LE	Comments	Table 2018	Ref.
SGLT2 inhibitors (dapagliflozin or empagliflozin) in symptomatic HFrEF patients with diabetes or not, receiving maximum optimized tolerate dose of beta-blocker, aldosterone antagonist, ACEI/ARB or ARNI to lower cardiovascular outcomes and progression of renal dysfunction.	I	A	**NEW:** SGLT2i are useful to reduce cardiovascular death and hospitalization for heart failure.	New	95–98
In *DAPA-HF* (*Dapagliflozin and Prevention of Adverse Outcomes in Heart Failure*), 4,744 HFrEF patients were randomly assigned to dapagliflozin or a placebo in addition to standard therapy, and 41.8% of them had DM2.^95^ The primary endpoint of cardiovascular death or worsening HF was significantly lower in the dapagliflozin group (26% reduction). When analyzed separately, there was a significant reduction in both cardiovascular death (18%) and worsening HF (30%), regardless the presence of DM2. The results reveal a new therapy for HF, already approved for that purpose. The *EMPEROR-Reduced* (*Empagliflozin Outcome Trial in Patients with Chronic Heart Failure and a Reduced Ejection Fraction*) trial assessed empagliflozin vs. a placebo, in addition to standard therapy, in 3,730 patients with HFrEF, 50.2% of which had DM2.^96^ Patients had more severe HF than those in *DAPA-HF*, with average LVEF of 27% vs. 31%, and over 70% of patients had LVEF under 30%, in addition to higher median NT-proBNP levels (1907 *versus* 1437 pg/mL). There was a 25% decrease in the primary endpoints of cardiovascular death or hospitalization for HF in favor of empagliflozin. When analyzed separately, there was no reduction in cardiovascular death, unlike the results from *DAPA-HF*. The benefit was once again observed regardless of the presence of DM2. The data confirm the results from *DAPA-HF* and reinforce the justification for using sodium-glucose cotransporter-2 inhibitors (SGLT2i) in HFrEF patients to reduce symptoms, improve quality of life, and lower the risk of hospitalization and cardiovascular death. The meta-analysis using results from the *DAPA-HF* and *EMPEROR-Reduced* trials, totaling 8,474 patients, found a 13% reduction in all-cause mortality (combined HR 0.87, 95% CI 0.77-0.98; p = 0.018) and a 14% reduction in death from cardiovascular disease (0.86, 95% CI 0.76 - 0.98; p = 0.027).(94) The use of SGLT2i was accompanied by a 26% relative reduction in combined risk for cardiovascular death or first hospitalization for HF (0.74,0.68–0.82; p < 0.0001), and a 25% eduction in the composite outcome of recurring hospitalizations for HF or cardiovascular death (0.75, 0.68–0.84; p < 0.0001). The risk of composite renal outcome was also lowered (0.62, 0.43–0.90; p = 0.013). The DAPA-HF subanalysis assessed the efficacy and safety of dapagliflozin use in HFrEF patients by baseline glomerular filtration rate (GFR) as well as the effects on dapagliflozin after randomization. The effect of dapagliflozin on primary (CV death or worsening HF) and secondary endpoints did not change with GFR (< 60 and ≥ 60 mL/min/1.73m^2^). A prespecified composite renal outcome (sustained > 50% reduction in GFR, terminal kidney disease or renal death) was also analyzed, along with worsening GFR throughout the study. Though dapagliflozin did not lower the composite renal outcome (RR = 0.71, 95% CI 0.44-1.16, p = 0.17), rates of worsening GFR were lower for dapagliflozin (-1.09) as compared to the placebo (-2.87), p < 0.001, for patients with our without DM2 (interaction p = 0.92).^95^ In the *EMPEROR-Reduced* trial, the annual rate of decline in GFR was slower in the empagliflozin group than in the placebo group (-0.55 vs. −2.28 mL/min/1.73 m^2^ per year, p < 0.001), and empagliflozin-treated patients had a lower risk of serious renal outcomes, regardless of the presence or absence of DM2.^96^ Data from a subanalysis of the *DAPA-HF* and *EMPEROR-Reduced* trials suggest the use of SGLT2 inhibitors is safe in patients with HFrEF and those with altered GFR, regardless of the presence or absence of DM2.					

*ACEI: angiotensin-converting enzyme II inhibitors; ARB: angiotensin II receptor blockers; ARNI: angiotensin II receptor-neprilysin inhibitors; DM2: type 2 diabetes mellitus; GFR: glomerular filtration rate; HF: heart failure; HFrEF: heart failure with reduced ejection fraction; LVEF: left ventricular ejection fraction; SGLT2i: sodium-glucose cotransporter-2 inhibitors.*

### 7.4. Treatment of Comorbidities in Heart Failure with Reduced Ejection Fraction

#### 7.4.1. Type 2 Diabetes (Table 7.4)

**Table 7.4:** Recommendations for use of SGLT2 inhibitors in preventing hospitalizations for HF in type 2 diabetes patients

Recommendations	Class	LE	Comments	Table 2018	Ref.
SGLT2 inhibitors (canagliflozin, dapagliflozin or empagliflozin) to prevent hospitalization for HF in patients with type 2 diabetes and cardiovascular risk factors for atherosclerosis or established atherosclerotic cardiovascular disease.	I	A	**NEW:** SGLT2i are useful to reduce hospitalization for heart failure in patients with DM2.	Item 5.2 (page 451)	99–101
SGLT2 inhibitors (dapagliflozin or empagliflozin) as initial antidiabetic medication associated or not with metformin in HFrEF patients.	I	A	**NEW:** SGLT2i are useful for diabetes treatment and reduction of cardiovascular and renal events.	New	102
The benefits of SGLT2i in type 2 diabetes (DM2) patients were first described in the *EMPA-REG OUTCOME (Empagliflozin, Cardiovascular Outcomes, and Mortality in Type 2 Diabetes)* trial, published in 2015, which assessed empagliflozin use in patients with DM2, established cardiovascular disease, and receiving standard treatment.^99^ Among those who received the medication, there was a significant reduction in major adverse cardiovascular events (MACE = CV death, nonfatal MI or nonfatal stroke) (HR: 0.86 (CI] 95%: 0.74-0.99), and a surprising reduction in hospitalization for heart failure (HHF) (HR: 0.65 (95% CI: 0.50-0.85). The *CANVAS-Program (Canagliflozin and Cardiovascular and Renal Events in Type 2 Diabetes)* trial, published in 2017, assessed canagliflozin in patients with DM2 at high risk for cardiovascular events receiving standard treatment. There was a reduction in combined primary outcomes (MACE = CV death, nonfatal MI or nonfatal stroke) and a 33% reduction in HHF (HR = 0.67, 95% CI: 0.52-0.87) as well as combined renal events.^100^ The *DECLARE-TIMI 58 (Dapagliflozin and Cardiovascular Outcomes in Type 2 Diabetes) trial* assessed dapagliflozin in patients with DM2 and atherosclerotic disease or multiple risk factors for atherosclerotic disease receiving standard treatment. There was no reduction in the combined primary endpoint (MACE = CV death, myocardial infarction or stroke). There was a 17 percent reduction in the combined endpoint of cardiovascular death and HHF, and 27 percent (HR: 0.73 (95% CI 0.61-0.88) for HHF.^101^ Recently, the *VERTIS-CV (Cardiovascular Outcomes with Ertugliflozin in Type 2 Diabetes)* trial assessed the use of ertugliflozin (not yet commercially available in Brazil) for patients with DM2, established cardiovascular disease, and receiving standard treatment. There was no reduction in the combined primary endpoint (MACE = CV death, myocardial infarction or stroke). However, a 30% decrease in HHF was observed.^100^ As a whole, the available data show the efficacy of SGLT2i in reducing the incidence of HF in groups of patients with DM2.^102^					

*DM2: type 2 diabetes mellitus; HHF: hospitalization for heart failure; MI: myocardial infarction; SGLT2i: sodium-glucose cotransporter-2 inhibitors.*

#### 7.4.2. Renal Dysfunction (Table 7.5)

**Table 7.5:** Recommendations for use of SGLT2 inhibitors in preventing worsening of renal function in HFrEF patients

Recommendations	Class	LE	Comments	Table 2018	Ref.
SGLT2 inhibitors (dapagliflozin or empagliflozin) in patients with HFrEF to prevent worsening of renal function in patients with and without diabetes, with GFR ≥ 20 mL/min/1.73 m^2^.	IIa	A	**NEW:** SGLT2i are useful to reduce progressive worsening of renal function in HFrEF.	New	95, 96, 98–104
In the *EMPEROR-Reduced* trial, the annual rate of decline in glomerular filtration rate (GFR) was slower in the empagliflozin group than in the placebo group (-0.55 vs. −2.28 mL/min/1,73 m^2^ per year, p < 0.001), and empagliflozin-treated patients had a lower risk of serious renal outcomes, regardless of the presence or absence of DM2.^96^ The *DAPA-HF* subanalysis assessed the efficacy and safety of dapagliflozin use in HFrEF patients by baseline GFR as well as the effects on dapagliflozin after randomization.^98^ In the *DAPA-HF* trial, dapagliflozin did not lead to lower composite renal outcomes (RR = 0.71, 95% CI 0.44-1.16, p = 0.17).^95^ However, in a subanalysis, rates of worsening GFR were lower for dapagliflozin (-1.09) as compared to a placebo (-2.87), p < 0.001, in patients with or without DM2. The *DAPA-CKD* (*Dapagliflozin in Patients with Chronic Kidney Disease*) trial randomized 4,304 patients with chronic kidney disease, GFR 25-75 mL/min/73 m^2^, and urinary albumin-creatinine ratio 200-5,000. Dapagliflozin led to lower rates of primary endpoints (consisting of sustained reduction in GFR of at least 50%, terminal kidney disease or CV or renal death) (9.2% with dapagliflozin vs. 14.5% with a placebo; [RR = 0.61, CI = 9%, 0.51-0.72; p < 0.001]. Death occurred for 101 members (4.5%) of the dapagliflozin group vs. 146 (6.8%) of the placebo group (RR = 0.69, 95% CI = 0.53-0.88, p = 0.004). Dapagliflozin lowered cardiovascular death or hospitalization for HF (0.67, 0.40-1.13 vs. 0.70, 0.52-0.94, respectively, P-interaction = 0,88). The results were consistent, both with and without DM2.^104^ Data from *EMPEROR-Reduced*, *DAPA-CKD* and the subanalysis of *DAPA-HF* suggest the use of SGLT2 inhibitors is safe in HFrEF and GFR alterations, regardless of the presence of DM2. They also show that SGLT2i may decrease renal function impairment in HFrEF patients.					

*DM2: type 2 diabetes mellitus; GFR: glomerular filtration rate; HFrEF: heart failure with reduced ejection fraction; SGLT2i: sodium-glucose cotransporter-2 inhibitors.*

#### 7.4.3. Iron Deficiency (Table 7.6)

**Table 7.6:** Recommendations for use of intravenous iron in HFrEF patients

Recommendation	Class	LE	Comments	Table 2018	Ref.
Intravenous ferric carboxymaltose replacement in patients with HFrEF and iron deficiency (serum ferritin below 100 ng/mL or between 100-299 ng/mL with transferrin saturation below 20%), even in the absence of anemia, to increase physical exercise capacity, improve quality of life, and reduce hospitalization rates.	IIa	A	2018 recommendation remains current.	Item 11.11 (page 470)	See 2018
Intravenous ferric carboxymaltose replacement in patients with HFrEF hospitalized for decompensated HF with iron deficiency (serum ferritin below 100 ng/mL or between 100-299 ng/mL with transferrin saturation below 20 percent) after clinical stabilization to reduce hospital readmission rates.	IIa	B	**NEW:** A multicenter randomized trial supports the recommendation.	New	105
In patients with chronic HF and iron deficiency, the use of intravenous ferric carboxymaltose led to improvements in symptoms, quality of life and hospitalization rates in previous meta-analyses and randomized trials.^106^^–^^108^ More recently, the multicenter, randomized, placebo-controlled *AFFIRM-AHF* trial assessed the effect of intravenous ferric carboxymaltose in 1,132 patients with HFrEF and iron deficiency (stable after an episode of HF decompensation and with iron deficiency — ferritin < 100 ng/mL or serum ferritin between 109 and 299 ng/mL associated with transferrin saturation below 20 percent) and found it to be safe and to reduce hospitalization for HF (217 vs. 294 hospitalizations; RR = 0.74; 95% CI 0.58-0.94, p = 0.013), though it had no direct impact on decreasing cardiovascular mortality.^105^^,^^109^					

*HF: heart failure; HFrEF: heart failure with reduced ejection fraction.*

### 7.5. Treatment Algorithm for Heart Failure with Reduced Ejection Fraction (Table 7.5)

**Figure 7.5:** Treatment algorithm for heart failure with reduced ejection fraction

**Em substituição a iECA /BRA.

*ACEI: angiotensin-converting enzyme inhibitors; ARB: angiotensin II receptor blockers; ARNI: angiotensin receptor-neprilysin inhibitor; CRT: cardiac resynchronization therapy; HF: heart failure; H-N: hydralazine-nitrate; ICD: implantable cardiac defibrillator; LBBB: left bundle branch block; LVEF: left ventricular ejection fraction; NYHA: New York Heart Association; SGLT2i: sodium-glucose cotransporter-2 inhibitors.*

## 8. Innovations in Other Areas Related to Heart Failure

### 8.1. Biomarkers in Heart Failure with Reduced Ejection Fraction (Table 8.1)

**Table 8.1:** Recommendations for the use of biomarkers in HFrEF patients

Recommendations	Class	LE	Comment	Table 2018	Ref.
Measurement of BNP or NT-proBNP when HF diagnosis is in question and as a screening test in primary care.	I	A	2018 recommendation remains current.	Item 4.3 (page 451)	See 2018
Measurement of BNP or NT-proBNP for prognostic stratification in patients with HF.	I	A	2018 recommendation remains current.	Item 4.3 (page 451)	See 2018
Measurement of BNP or NT-proBNP as a complement to physical examination to assess response to treatment in HF patients in case of questions about their clinical status.	IIa	B	**MODIFIED:** Two recent studies, one randomized, the other observational, support that indication.	Item 4.3 (page 451)	84, 110
Serial measurements of BNP or NT-proBNP to guide treatment, with biomarker targets.	IIb	B	**MODIFIED:** A recent meta-analysis, including data from the *Guide-IT* trial, support that indication.	Item 4.3 (page 451)	111, 112
Natriuretic peptides may be used to assess patient response to a given treatment. In terms of strategy, the treatment is clinically directed and the biomarker is measured before and after with no specific target. New studies have come up to confirm what had already been shown by a subanalysis of the *PARADIGM-HF* trial, where patients who had lowered their NT-proBNP to below 1000 pg/mL after the initiation of enalapril or sacubitril-valsartan had lower mortality and fewer hospitalizations for HF.^113^ In the *PIONEER-HF* trial following up on patients hospitalized for HF after discharge, sacubitril-valsartan produced a greater decrease in NT-proBNP than enalapril after 4 weeks (46.7 vs 25.3 percent), leading to a smaller number of events from sacubitril-valsartan use.^84^ In the *Prove-HF* trial, where chronic HF patients used sacubitril-valsartan, there was a significant decrease in NT-proBNP after the medication had been used for 14 days. The NT-proBNP decrease was associated with reverse remodeling during the 12 months of follow-up and had a smaller event rate.^110^ Conversely, the use of peptides to guide treatment (with natriuretic peptide targets) is controversial. Though the strategy was not superior to conventional management in the *Guide-IT* trial, previous surveys have found different results.^112^ Another trial, *Protect,*^114^, NT-proBNP-guided therapy was superior to standard of care, with reduced event rates, improved quality of life, and favorable effects on cardiac remodeling. The *Time-CHF*^115^ and Battlescarred^116^ trials found the strategy led to decreases in mortality in patients under the age of 75. In addition, a recent meta-analysis including 4,554 patients and incorporating patients from the *Guide-IT* trial found lower hospitalization rates and all-cause mortality from natriuretic peptide-guided treatment.^111^					

*HF: heart failure; NT-proBNP: N-terminal portion of B-type natriuretic peptide.*

### 8.2. Immunizations in Heart Failure (Table 8.2)

**Table 8.2:** Recommendations for immunizations for HFrEF patients

Recommendations	Class	LE	Comment	Table 2018	Ref.
Influenza vaccine to prevent influenza-related morbidity and mortality in HF.	I	B	**MODIFIED**: New retrospective studies have shown benefits in reducing mortality rates.	Item 6.7 (page 454)	117–120
Pneumococcal vaccine to prevent pneumococcal-related morbidity and mortality in HF.	I	C	2018 recommendation remains current.	Item 6.7 (page 454)	See 2018
Until recently, there was no data on the impact of influenza on outcomes for patients with HF. However, recent population-based studies have shown the relationship between seasonality and a higher number of hospitalizations for HF, evident on four consecutive periods.^117^ In a subanalysis of the *Paradigm* trial, 21% of participants were vaccinated against influenza, leading to a 19% decrease in overall mortality after adjusting for propensity.^118^ A Danish cohort study of 134,038 HF patients receiving ≥1 vaccinations between 2003 and 2015, resulted in an 18% decrease in all-cause mortality; more importantly, greater cumulative number of vaccinations was associated with an 28% reduced risk in total mortality and a 29% decrease in cardiovascular mortality.^119^ A database study of 6,435 HF patients, out of which 695 had been vaccinated before or during the 2017/2018 winter seasons, found a 22% decrease in total mortality and a 17% decrease in cardiovascular death or hospitalizations for HF. The benefits from vaccination on total mortality were greater for patients over the age of 70, with an over 25% decrease.^120^ There are no studies on the impact of pneumococcal vaccines on outcomes. Several prospective studies are currently recruiting patients					

*HF: heart failure.*

### 8.3. Indications for Genetic Assessment in Cardiomyopathies and Heart Failure (Table 8.3)

**Table 8.3:** Recommendations for genetic assessments for patients with cardiomyopathies and HF

Recommendations	Class	LE	Comment	Table 2018	Ref.
Genetic counseling for patients and family members with inherited cardiomyopathies and previously identified mutations.	I	C	**NEW:** Advances in molecular genetic assessment techniques enable the early identification of inherited cardiomyopathies, supporting the subclassification of clinical syndromes and individualized treatment.	New	121–125
Screening test to 1^st^ degree relatives of patients with inherited cardiomyopathies.	I	C		New	121–125
Sequencing of the transthyretin gene in patients diagnosed with transthyretin cardiac amyloidosis.	I	C		New	121–125
Molecular genetic assessment to investigate the etiology and evaluate the prognosis of patients with inherited cardiomyopathy phenotype.	IIa	C		New	121–125
Routine molecular genetic assessment for HF patients.	III	C		New	121–125
The incorporation of next-generation sequencing has increased the sensitivity of genetic testing, allowing for early early diagnosis for future interventions.^121^ Consequently, molecular assessments have allowed routine genetic testing for patients with inherited cardiomyopathies, such as hypertrophic, restrictive and/or dilated arrhythmogenic cardiomyopathies, and non-compacted myocardium, due to its potential to provide more individualized and precise counseling for patients with these conditions as well as for their family members.^122^ One clear example of this need is the distinction between wild-type and inherited transthyretin cardiac amyloidosis (ATTR) as cascade genetic testing allows at-risk relatives to be definitively identified. It should be highlighted that current therapies for ATTR are particularly beneficial when initiated during the early stages of the disease, as described in item 2, Table 2.4.^123^ Advances in prognostic assessment involving genes with high arrhythmogenic potential have also been described for dilated and arrhythmogenic cardiomyopathies.^124^^,^^125^ Thus, it is important to pursue more efficient uses of genetic information, especially in family counseling, leading to safe and sustainable results in the care of these patients and their family members.					

*HF: heart failure.*

## 9. Perspectives in Heart Failure – New Molecules

### 9.1. Guanylate Cyclase Stimulators (Table 9.1)

**Table 9.1:** Guanylate cyclase stimulators for the treatment of HFrEF patients

Notes	Comment	Table 2018	Ref.
Vericiguat in patients with LVEF lower than 45%, NYHA II – IV to reduce morbidity, especially in patients with frequent hospitalizations despite optimized guideline-directed medical therapy.	**POTENTIAL:** The observations described herein reflect data from recent studies on this new class of drug. However, it has not been approved by Anvisa for use in Brazil yet.	New	126, 127
Vericiguat acts by supplying the relative deficit of cyclic GMP production in HF patients^126^ and was assessed in a multicenter, randomized, double-blind, placebo-controlled trial with HFrEF patients, the *VICTORIA (Vericiguat in Patients with Heart Failure and Reduced Ejection Fraction) trial.* In the *Victoria*, 5050 patients with HFrEF, with LVEF lower than 45%, NYHA II-IV, were randomized to receive vericiguat 10 mg/day orally or placebo, in addition to guideline-directed medical therapy. The primary endpoint was cardiovascular death or first hospitalization for HF. In an 11-month period, the primary endpoint occurred in 35.5% of the vericiguat group and 38.5% of the placebo group, which represents a number needed to treat (NNT) of 24 to save one life over 11 months. The benefit of the composite outcome was primarily attributed to the reduction in hospitalization rates, with no statistically significant impact on cardiovascular or overall mortality.^127^ The drug could potentially join the set of medications acting on symptoms and readmissions for HFrEF patients, representing an additional option: for patients who undergo frequent hospitalizations despite optimized therapy; who have impaired kidney function, since patients eligible for the trial had GFR above 15%; or who are intolerance to other medications. It should be stressed that this medication class is contraindicated in combination with nitrates.					

*GFR: glomerular filtration rate; HF: heart failure; HFrEF: heart failure with reduced ejection fraction; LVEF: left ventricular ejection fraction; NYHA: New York Heart Association.*

### 9.2. Selective Cardiac Myosin Activator (Table 9.2)

**Table 9.2:** Omecamtiv mercabil in the treatment of HFrEF patients

Notes	Comment	Table 2018	Ref.
Omecamtiv mecarbil in patients with acute or chronic HFrEF.	**POTENTIAL:** The observations described herein reflect data from recent studies on this new class of drug. However, it has not been approved by Anvisa for use in Brazil yet.	New	128–131
Omecamtiv mecarbil selectively binds to cardiac myosin resulting in activation and increase in rate of ATP hydrolysis, and the transition of myosin to the strongly actin-bound force-generating state, improving impaired ventricular contraction in cases of HFrEF. Its mechanism of action is different from mechanisms of the current triple therapy, which inhibits neurohormonal stimulation. Mechanistic studies such as the *ATOMIC-AHF* (*Acute Treatment with Omecamtiv Mecarbil to Increase Contractility in Acute Heart Failure*)^128^ and *COSMIC-HF* (*Chronic Oral Study of Myosin Activation to Increase Contractility in Heart Failure*)^129^ trials, showed that the medication improved contractility, ejection fraction, ejected volume and cardiac output, in addition to other parameters of improved cardiac function. Studies show it promotes decreases in NT-proBNP levels. High troponin levels were also identified without clinical changes in the studies. The *Atomic-AHF* trial, however, with acute HF patients, found no reduction in dyspnea among patients in the treatment group. In the recently-published *GALACTIC-HF (Cardiac Myosin Activation with Omecamtiv Mecarbil in Systolic Heart Failure)* randomized controlled trial, patients with HFrEF who received omecantiv mecarbil had lower risk of composite outcomes from an HF event (defined as hospitalization or unplanned visits due to worsening HF) or cardiovascular death than those who received a placebo.^130^^,^^131^ However, when assessed individually, there was no difference in the following secondary outcomes: all-cause mortality, cardiovascular death, first hospitalization for HF, or changes in the *Kansas City Cardiomyopathy Questionnaire* quality of life score.					

*HF: heart failure; HFrEF: heart failure with reduced ejection fraction.*

## Appendix

**Table:**

Emerging Topics Update of the Brazilian Heart Failure Guideline – 2021	
The report below lists declarations of interest as reported to the SBC by the experts during the period of the development of these update, 2020.	
Expert	Type of relationship with industry
Aguinaldo F. Freitas Jr.	Financial declaration A - Economically relevant payments of any kind made to (i) you, (ii) your spouse/partner or any other person living with you, (iii) any legal person in which any of these is either a direct or indirect controlling owner, business partner, shareholder or participant; any payments received for lectures, lessons, training instruction, compensation, fees paid for participation in advisory boards, investigative boards or other committees, etc. From the brazilian or international pharmaceutical, orthosis, prosthesis, equipment and implants industry: - Novartis: Heart failure; Servier: Heart failure; Bayer: Anticoagulation.
Andréia Biolo	Nothing to be declared
Antonio Carlos Pereira Barretto	Other relationships Funding of continuing medical education activities, including travel, accommodation and registration in conferences and courses, from the brazilian or international pharmaceutical, orthosis, prosthesis, equipment and implants industry: - Novartis: Sacubitril Valsartana; AstraZeneca: Dapagliflozina, Succinato de Metoprolol, Candesartana; Servier: Ivabradina.
Antônio José Lagoeiro Jorge	Other relationships Funding of continuing medical education activities, including travel, accommodation and registration in conferences and courses, from the brazilian or international pharmaceutical, orthosis, prosthesis, equipment and implants industry: - Novartis: Entresto.
Bruno Biselli	Nothing to be declared
Carlos Eduardo Lucena Montenegro	Financial declaration A - Economically relevant payments of any kind made to (i) you, (ii) your spouse/partner or any other person living with you, (iii) any legal person in which any of these is either a direct or indirect controlling owner, business partner, shareholder or participant; any payments received for lectures, lessons, training instruction, compensation, fees paid for participation in advisory boards, investigative boards or other committees, etc. From the brazilian or international pharmaceutical, orthosis, prosthesis, equipment and implants industry: - AstraZeneca: Lectures; Novartis: Lectures; Servier: Lectures; Merck: Lectures. Other relationships Funding of continuing medical education activities, including travel, accommodation and registration in conferences and courses, from the brazilian or international pharmaceutical, orthosis, prosthesis, equipment and implants industry: - Pfizer: Travel to Congress; AstraZeneca: Travel to Symposium; Novartis: Travel to Symposium.
Carlos Eduardo Lucena Montenegro	Financial declaration B - Research funding under your direct/personal responsibility (directed to the department or institution) from the brazilian or international pharmaceutical, orthosis, prosthesis, equipment and implants industry: - FAPERJ: Clinical research; Boehringer: International Multicenter Clinical Research Participant.
Dirceu Rodrigues de Almeida	Nothing to be declared
Edimar Alcides Bocchi	Financial declaration A - Economically relevant payments of any kind made to (i) you, (ii) your spouse/partner or any other person living with you, (iii) any legal person in which any of these is either a direct or indirect controlling owner, business partner, shareholder or participant; any payments received for lectures, lessons, training instruction, compensation, fees paid for participation in advisory boards, investigative boards or other committees, etc. From the brazilian or international pharmaceutical, orthosis, prosthesis, equipment and implants industry: - AstraZeneca: ISGLT2; Bayer: ISGLT2, Vericiguat; Boehringer: ISGLT2.
Edval Gomes dos Santos Júnior	Nothing to be declared
Estêvão Lanna Figueiredo	Financial declaration B - Research funding under your direct/personal responsibility (directed to the department or institution) from the brazilian or international pharmaceutical, orthosis, prosthesis, equipment and implants industry: - AstraZeneca: Dapagliflozina; Boehringer: Empagliflozina; Pfizer: Apixabana; Novartis
Evandro Tinoco Mesquita	Other relationships Employment relationship with the brazilian or international pharmaceutical, orthosis, prosthesis, equipment and implants industry, as well as any employment relationship with health insurance companies or medical audit companies (including part-time jobs) in the year to which your declaration refers: - UnitedHealth Group.
Fabiana G. Marcondes-Braga	Financial declaration A - Economically relevant payments of any kind made to (i) you, (ii) your spouse/partner or any other person living with you, (iii) any legal person in which any of these is either a direct or indirect controlling owner, business partner, shareholder or participant; any payments received for lectures, lessons, training instruction, compensation, fees paid for participation in advisory boards, investigative boards or other committees, etc. From the brazilian or international pharmaceutical, orthosis, prosthesis, equipment and implants industry: - Novartis: Lectures; AstraZeneca: Lectures and Advisory Council; Boehringer: Advisory Council.
Fábio Fernandes	Financial declaration A - Economically relevant payments of any kind made to (i) you, (ii) your spouse/partner or any other person living with you, (iii) any legal person in which any of these is either a direct or indirect controlling owner, business partner, shareholder or participant; any payments received for lectures, lessons, training instruction, compensation, fees paid for participation in advisory boards, investigative boards or other committees, etc. From the brazilian or international pharmaceutical, orthosis, prosthesis, equipment and implants industry: - Pfizer: Tafamidis; Alnylan: Patisiran.
Fabio Serra Silveira	Financial declaration A - Economically relevant payments of any kind made to (i) you, (ii) your spouse/partner or any other person living with you, (iii) any legal person in which any of these is either a direct or indirect controlling owner, business partner, shareholder or participant; any payments received for lectures, lessons, training instruction, compensation, fees paid for participation in advisory boards, investigative boards or other committees, etc. From the brazilian or international pharmaceutical, orthosis, prosthesis, equipment and implants industry: - Novartis: Entresto; AstraZeneca: Forxiga; Servier: Procoralan. B - Research funding under your direct/personal responsibility (directed to the department or institution) from the brazilian or international pharmaceutical, orthosis, prosthesis, equipment and implants industry: - Amgen: Omecantiv mecarbil. Other relationships Funding of continuing medical education activities, including travel, accommodation and registration in conferences and courses, from the brazilian or international pharmaceutical, orthosis, prosthesis, equipment and implants industry: - Ache: Hypertension.
Felix José Alvarez Ramires	Financial declaration A - Economically relevant payments of any kind made to (i) you, (ii) your spouse/partner or any other person living with you, (iii) any legal person in which any of these is either a direct or indirect controlling owner, business partner, shareholder or participant; any payments received for lectures, lessons, training instruction, compensation, fees paid for participation in advisory boards, investigative boards or other committees, etc. From the brazilian or international pharmaceutical, orthosis, prosthesis, equipment and implants industry: - Novartis: Sacubitril/Valsartana; Pfizer: Patisiran; Merck: Vericiquat; Amgen.
Fernando Antibas Atik	Nothing to be declared
Fernando Bacal	Nothing to be declared
Flávio de Souza Brito	Financial declaration A - Economically relevant payments of any kind made to (i) you, (ii) your spouse/partner or any other person living with you, (iii) any legal person in which any of these is either a direct or indirect controlling owner, business partner, shareholder or participant; any payments received for lectures, lessons, training instruction, compensation, fees paid for participation in advisory boards, investigative boards or other committees, etc. From the brazilian or international pharmaceutical, orthosis, prosthesis, equipment and implants industry: - Novartis: Entresto; Servier: Procoralan; Merck: Concor.
Germano Emílio Conceição Souza	Financial declaration A - Economically relevant payments of any kind made to (i) you, (ii) your spouse/partner or any other person living with you, (iii) any legal person in which any of these is either a direct or indirect controlling owner, business partner, shareholder or participant; any payments received for lectures, lessons, training instruction, compensation, fees paid for participation in advisory boards, investigative boards or other committees, etc. From the brazilian or international pharmaceutical, orthosis, prosthesis, equipment and implants industry: - Novartis: Heart failure; Merck: Heart failure. B - Research funding under your direct/personal responsibility (directed to the department or institution) from the brazilian or international pharmaceutical, orthosis, prosthesis, equipment and implants industry: - Bayer: Rivaroxabana Other relationships Any economically relevant equity interest in companies in the healthcare or education industry or in any companies competing with or supplying to SBC: - Healthcare: Medical services as a legal person, assistance area - Education: Technical education company for classes and courses in the health area
Gustavo Calado de Aguiar Ribeiro	Nothing to be declared
Humberto Villacorta	Financial declaration A - Economically relevant payments of any kind made to (i) you, (ii) your spouse/partner or any other person living with you, (iii) any legal person in which any of these is either a direct or indirect controlling owner, business partner, shareholder or participant; any payments received for lectures, lessons, training instruction, compensation, fees paid for participation in advisory boards, investigative boards or other committees, etc. From the brazilian or international pharmaceutical, orthosis, prosthesis, equipment and implants industry: - Novartis: Heart failure; Roche: Biomarkers; Servier: Heart failure. C - Personal research funding paid by the brazilian or international pharmaceutical, orthosis, prosthesis, equipment and implants industry: - Roche: GDF-15
Jefferson Luis Vieira	Nothing to be declared
João David de Souza Neto	Nothing to be declared
João Manoel Rossi Neto	Financial declaration A - Economically relevant payments of any kind made to (i) you, (ii) your spouse/partner or any other person living with you, (iii) any legal person in which any of these is either a direct or indirect controlling owner, business partner, shareholder or participant; any payments received for lectures, lessons, training instruction, compensation, fees paid for participation in advisory boards, investigative boards or other committees, etc. From the brazilian or international pharmaceutical, orthosis, prosthesis, equipment and implants industry: - Novartis: Lectures; AstraZeneca: Lectures.
José Albuquerque de Figueiredo Neto	Financial declaration A - Economically relevant payments of any kind made to (i) you, (ii) your spouse/partner or any other person living with you, (iii) any legal person in which any of these is either a direct or indirect controlling owner, business partner, shareholder or participant; any payments received for lectures, lessons, training instruction, compensation, fees paid for participation in advisory boards, investigative boards or other committees, etc. From the brazilian or international pharmaceutical, orthosis, prosthesis, equipment and implants industry: - Novartis: Heart failure.
Lídia Ana Zytynski Moura	Financial declaration A - Economically relevant payments of any kind made to (i) you, (ii) your spouse/partner or any other person living with you, (iii) any legal person in which any of these is either a direct or indirect controlling owner, business partner, shareholder or participant; any payments received for lectures, lessons, training instruction, compensation, fees paid for participation in advisory boards, investigative boards or other committees, etc. From the brazilian or international pharmaceutical, orthosis, prosthesis, equipment and implants industry: - Novartis: Entresto; AstraZeneca: Forxiga. B - Research funding under your direct/personal responsibility (directed to the department or institution) from the brazilian or international pharmaceutical, orthosis, prosthesis, equipment and implants industry: - AstraZeneca: Forxiga.
Livia Adams Goldraich	Nothing to be declared
Luís Beck-da-Silva	Financial declaration A - Economically relevant payments of any kind made to (i) you, (ii) your spouse/partner or any other person living with you, (iii) any legal person in which any of these is either a direct or indirect controlling owner, business partner, shareholder or participant; any payments received for lectures, lessons, training instruction, compensation, fees paid for participation in advisory boards, investigative boards or other committees, etc. From the brazilian or international pharmaceutical, orthosis, prosthesis, equipment and implants industry: - Novartis: Heart failure; AstraZeneca: Heart failure. B - Research funding under your direct/personal responsibility (directed to the department or institution) from the brazilian or international pharmaceutical, orthosis, prosthesis, equipment and implants industry: - Amgen: Heart failure.
Luis Eduardo Rohde	Financial declaration A - Economically relevant payments of any kind made to (i) you, (ii) your spouse/partner or any other person living with you, (iii) any legal person in which any of these is either a direct or indirect controlling owner, business partner, shareholder or participant; any payments received for lectures, lessons, training instruction, compensation, fees paid for participation in advisory boards, investigative boards or other committees, etc. From the brazilian or international pharmaceutical, orthosis, prosthesis, equipment and implants industry: - Astrazeneca: dapaglifozina; Novartis: Sacubitril-Valsartana; Amgen: Omecamtiv Mecarbil; Merck; Bayer.
Luiz Claudio Danzmann	Financial declaration A - Economically relevant payments of any kind made to (i) you, (ii) your spouse/partner or any other person living with you, (iii) any legal person in which any of these is either a direct or indirect controlling owner, business partner, shareholder or participant; any payments received for lectures, lessons, training instruction, compensation, fees paid for participation in advisory boards, investigative boards or other committees, etc. From the brazilian or international pharmaceutical, orthosis, prosthesis, equipment and implants industry: - Novartis: Entresto; AstraZeneca: Forxiga; Servier: Procoralan.
Manoel Fernandes Canesin	Nothing to be declared
Marcelo Imbroinise Bittencourt	Financial declaration A - Economically relevant payments of any kind made to (i) you, (ii) your spouse/partner or any other person living with you, (iii) any legal person in which any of these is either a direct or indirect controlling owner, business partner, shareholder or participant; any payments received for lectures, lessons, training instruction, compensation, fees paid for participation in advisory boards, investigative boards or other committees, etc. From the brazilian or international pharmaceutical, orthosis, prosthesis, equipment and implants industry: - Geneone - Dasa: Genetic testing; Sanofi: Enzyme replacement therapy; AstraZeneca: Forxiga.
Marcelo Westerlund Montera	Nothing to be declared
Marcely Gimenes Bonatto	Financial declaration A - Economically relevant payments of any kind made to (i) you, (ii) your spouse/partner or any other person living with you, (iii) any legal person in which any of these is either a direct or indirect controlling owner, business partner, shareholder or participant; any payments received for lectures, lessons, training instruction, compensation, fees paid for participation in advisory boards, investigative boards or other committees, etc. From the brazilian or international pharmaceutical, orthosis, prosthesis, equipment and implants industry: - Novartis: Sacubitril/Valsartana; AstraZeneca: Forxiga
Marcus Vinícius Simões	Financial declaration A - Economically relevant payments of any kind made to (i) you, (ii) your spouse/partner or any other person living with you, (iii) any legal person in which any of these is either a direct or indirect controlling owner, business partner, shareholder or participant; any payments received for lectures, lessons, training instruction, compensation, fees paid for participation in advisory boards, investigative boards or other committees, etc. From the brazilian or international pharmaceutical, orthosis, prosthesis, equipment and implants industry: - Novartis: Entresto; AstraZeneca: Dapagliflozina. B - Research Funding Under Your Direct/Personal Responsibility (Directed To The Department Or Institution) From The Brazilian Or International Pharmaceutical, Orthosis, Prosthesis, Equipment And Implants Industry: - Amgen: Omecamtiv/Mecarbil; Beringher Ingelheim: Empagliflozina.
Maria da Consolação Vieira Moreira	Nothing to be declared
Miguel Morita Fernandes-Silva	Financial declaration A - Economically relevant payments of any kind made to (i) you, (ii) your spouse/partner or any other person living with you, (iii) any legal person in which any of these is either a direct or indirect controlling owner, business partner, shareholder or participant; any payments received for lectures, lessons, training instruction, compensation, fees paid for participation in advisory boards, investigative boards or other committees, etc. From the brazilian or international pharmaceutical, orthosis, prosthesis, equipment and implants industry: - Novartis: Heart failure C - Personal research funding paid by the brazilian or international pharmaceutical, orthosis, prosthesis, equipment and implants industry: - Amgen: Omecamtiv/Heart failure; Beringher Ingelheim: Empagliflozina.
Mônica Samuel Avila	Nothing to be declared
Mucio Tavares de Oliveira Junior	Financial declaration B - Research funding under your direct/personal responsibility (directed to the department or institution) from the brazilian or international pharmaceutical, orthosis, prosthesis, equipment and implants industry: - Torrent do Brasil: Developing forms of drugs; Sanofi Pasteur: Flu vaccine
Nadine Clausell	Nothing to be declared
Odilson Marcos Silvestre	Nothing to be declared
Otavio Rizzi Coelho Filho	Financial declaration A - Economically relevant payments of any kind made to (i) you, (ii) your spouse/partner or any other person living with you, (iii) any legal person in which any of these is either a direct or indirect controlling owner, business partner, shareholder or participant; any payments received for lectures, lessons, training instruction, compensation, fees paid for participation in advisory boards, investigative boards or other committees, etc. From the brazilian or international pharmaceutical, orthosis, prosthesis, equipment and implants industry: - Pfizer: Cardiac amyloidosis; Alnylam: Cardiac amyloidosis; AstraZeneca: Heart failure; Novartis. B - Research funding under your direct/personal responsibility (directed to the department or institution) from the brazilian or international pharmaceutical, orthosis, prosthesis, equipment and implants industry: - Pfizer: Cardiac amyloidosis. Other relationships Funding of continuing medical education activities, including travel, accommodation and registration in conferences and courses, from the brazilian or international pharmaceutical, orthosis, prosthesis, equipment and implants industry: - AstraZeneca: Heart failure; Pfizer: Heart failure.
Pedro Vellosa Schwartzmann	Financial declaration A - Economically relevant payments of any kind made to (i) you, (ii) your spouse/partner or any other person living with you, (iii) any legal person in which any of these is either a direct or indirect controlling owner, business partner, shareholder or participant; any payments received for lectures, lessons, training instruction, compensation, fees paid for participation in advisory boards, investigative boards or other committees, etc. From the brazilian or international pharmaceutical, orthosis, prosthesis, equipment and implants industry: - Novartis: Entresto; Servier: Ivabradina; AstraZeneca: Dapagliflozina; Merck: Serono. B - Research funding under your direct/personal responsibility (directed to the department or institution) from the brazilian or international pharmaceutical, orthosis, prosthesis, equipment and implants industry: - Novartis: investigacional; Eidos: AG10. Other relationships Funding of continuing medical education activities, including travel, accommodation and registration in conferences and courses, from the brazilian or international pharmaceutical, orthosis, prosthesis, equipment and implants industry: - Bayer: Rivaroxaban; AstraZeneca: Dapagliflozina.
Reinaldo Bulgarelli Bestetti	Nothing to be declared
Ricardo Mourilhe-Rocha	Financial declaration A - Economically relevant payments of any kind made to (i) you, (ii) your spouse/partner or any other person living with you, (iii) any legal person in which any of these is either a direct or indirect controlling owner, business partner, shareholder or participant; any payments received for lectures, lessons, training instruction, compensation, fees paid for participation in advisory boards, investigative boards or other committees, etc. From the brazilian or international pharmaceutical, orthosis, prosthesis, equipment and implants industry: - Boehringer: Empagliflozina; Novartis: Sacubitril/Valsartana. B - Research funding under your direct/personal responsibility (directed to the department or institution) from the brazilian or international pharmaceutical, orthosis, prosthesis, equipment and implants industry: - PROADI/SUS: Telemedicine; Boehringer: Empagliflozina.
Sabrina Bernadez-Pereira	Nothing to be declared
Salvador Rassi	Financial declaration A - Economically relevant payments of any kind made to (i) you, (ii) your spouse/partner or any other person living with you, (iii) any legal person in which any of these is either a direct or indirect controlling owner, business partner, shareholder or participant; any payments received for lectures, lessons, training instruction, compensation, fees paid for participation in advisory boards, investigative boards or other committees, etc. From the brazilian or international pharmaceutical, orthosis, prosthesis, equipment and implants industry: - Novartis: Entresto; Servier: Procoralan. B - Research funding under your direct/personal responsibility (directed to the department or institution) from the brazilian or international pharmaceutical, orthosis, prosthesis, equipment and implants industry: - Novartis: Entresto; Servier: Procoralan; Boehringer Ingelheim: Jardiance. Other relationships Funding of continuing medical education activities, including travel, accommodation and registration in conferences and courses, from the brazilian or international pharmaceutical, orthosis, prosthesis, equipment and implants industry: - Novartis: Entresto; Servier: Procoralan.
Sandrigo Mangini	Financial declaration A - Economically relevant payments of any kind made to (i) you, (ii) your spouse/partner or any other person living with you, (iii) any legal person in which any of these is either a direct or indirect controlling owner, business partner, shareholder or participant; any payments received for lectures, lessons, training instruction, compensation, fees paid for participation in advisory boards, investigative boards or other committees, etc. From the brazilian or international pharmaceutical, orthosis, prosthesis, equipment and implants industry: - Novartis: Sacubitril/Valsartan; Pfizer: Rare diseases. Other relationships Funding of continuing medical education activities, including travel, accommodation and registration in conferences and courses, from the brazilian or international pharmaceutical, orthosis, prosthesis, equipment and implants industry: - Pfizer: Rare diseases.
Silvia Marinho Martins Alves	Nothing to be declared
Silvia Moreira Ayub Ferreira	Financial declaration A - Economically relevant payments of any kind made to (i) you, (ii) your spouse/partner or any other person living with you, (iii) any legal person in which any of these is either a direct or indirect controlling owner, business partner, shareholder or participant; any payments received for lectures, lessons, training instruction, compensation, fees paid for participation in advisory boards, investigative boards or other committees, etc. From the brazilian or international pharmaceutical, orthosis, prosthesis, equipment and implants industry: - Abbott: Mitraclip; Novartis: Entresto. Other relationships Funding of continuing medical education activities, including travel, accommodation and registration in conferences and courses, from the brazilian or international pharmaceutical, orthosis, prosthesis, equipment and implants industry: - Abbott: Heartmate II e HeartMate 3
Victor Sarli Issa	Nothing to be declared
